# A Temporal Proteomic Map of Epstein-Barr Virus Lytic Replication in B Cells

**DOI:** 10.1016/j.celrep.2017.04.062

**Published:** 2017-05-16

**Authors:** Ina Ersing, Luis Nobre, Liang Wei Wang, Lior Soday, Yijie Ma, Joao A. Paulo, Yohei Narita, Camille W. Ashbaugh, Chang Jiang, Nicholas E. Grayson, Elliott Kieff, Steven P. Gygi, Michael P. Weekes, Benjamin E. Gewurz

**Affiliations:** 1 Division of Infectious Disease, Department of Medicine, Brigham & Women’s Hospital, Harvard Medical School, 181 Longwood Avenue, Boston, MA 02115, USA; 2 Institut für Klinische und Molekulare Virologie, Friedrich-Alexander-Universität Erlangen-Nürnberg, 91054 Erlangen, Germany; 3 Cambridge Institute for Medical Research, University of Cambridge, Hills Road, Cambridge CB2 0XY, UK; 4 Department of Cell Biology, Harvard Medical School, 240 Longwood Avenue, Boston, MA 02115, USA; 5 Wellcome Trust Sanger Institute, Hinxton, Cambridge, CB10 1HH, UK; 6 Department of Immunobiology and Microbiology, Harvard Medical School, 77 Avenue Louis Pasteur, Boston, MA 02115, USA; 7 Harvard Virology Program, Harvard Medical School, 77 Avenue Louis Pasteur, Boston, MA 02115, USA

**Keywords:** Epstein-Barr virus, herpesvirus, lytic replication, quantitative proteomics, tandem mass tag, host-pathogen interaction, immune evasion, B cell receptor, complement, viral evasion

## Abstract

Epstein-Barr virus (EBV) replication contributes to multiple human diseases, including infectious mononucleosis, nasopharyngeal carcinoma, B cell lymphomas, and oral hairy leukoplakia. We performed systematic quantitative analyses of temporal changes in host and EBV proteins during lytic replication to gain insights into virus-host interactions, using conditional Burkitt lymphoma models of type I and II EBV infection. We quantified profiles of >8,000 cellular and 69 EBV proteins, including >500 plasma membrane proteins, providing temporal views of the lytic B cell proteome and EBV virome. Our approach revealed EBV-induced remodeling of cell cycle, innate and adaptive immune pathways, including upregulation of the complement cascade and proteasomal degradation of the B cell receptor complex, conserved between EBV types I and II. Cross-comparison with proteomic analyses of human cytomegalovirus infection and of a Kaposi-sarcoma-associated herpesvirus immunoevasin identified host factors targeted by multiple herpesviruses. Our results provide an important resource for studies of EBV replication.

## Highlights

• Unbiased global analysis of host and EBV proteome remodeling in lytic replication
• Temporal profiles of >8,000 host and 69 viral proteins, using type I and II EBV
• Both EBV types target the B cell receptor complex for degradation
• Conserved EBV and HCMV lytic cycle host targets are identified

## Graphical Abstract

**Figure:**
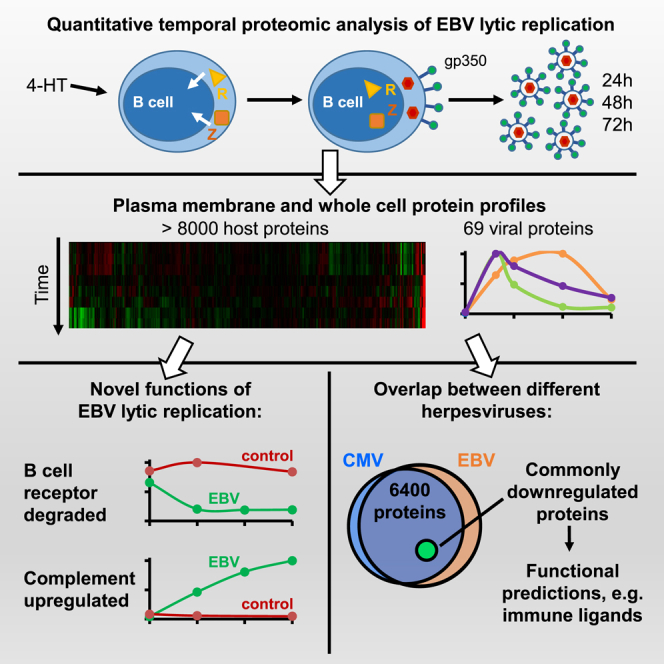

## Teaser

Ersing et al. present a temporal proteomic map of EBV B cell lytic replication. Tandem-mass-tag-based proteomics uncover extensive remodeling of the human proteome by EBV, conserved across the two major EBV strains. Cell-cycle, innate, and adaptive immune pathways are modulated, complement is upregulated, and the B cell receptor is degraded by infection.

## Introduction

Epstein-Barr virus (EBV) is a gamma-herpesvirus that establishes persistent infection in >95% of adults worldwide. Two distinct strains of EBV have been identified, referred to as type I and II (Kieff and Rickinson, 2007). Following salivary transmission, EBV replicates in or translocates through epithelial cells and infects tonsillar B cells to establish lifelong B cell infection (Thorley-Lawson, 2015, Tugizov et al., 2013). Periodic viral reactivation re-infects the tonsillar epithelium, in which further rounds of lytic replication amplify the virus population that is secreted into saliva (Kenney and Mertz, 2014, Laichalk and Thorley-Lawson, 2005). EBV lytic reactivation is central to the virus life cycle and to most EBV-related diseases.

EBV is the etiologic agent of infectious mononucleosis and is closely linked to the pathogenesis of multiple human malignancies, with 200,000 EBV-associated cancers reported annually (Cohen et al., 2011). Lytic viral replication is implicated in the pathogenesis of nasopharyngeal carcinoma and oral hairy leukoplakia (Chien et al., 2001, Tsai et al., 2013) and may contribute to growth of B cell tumors, particularly in immunodeficiency (Arvey et al., 2012, Ma et al., 2011). The incidences of EBV-related Hodgkin lymphoma continue to rise in individuals with HIV infection despite antiretroviral therapy (Powles et al., 2009).

Upon lytic reactivation, EBV genes are sequentially expressed in immediate-early (IE), early (E,) and late (L) phases. The immediate early transcription factors ZTA (encoded by BZLF1) and RTA (encoded by BRLF1) jointly trigger the EBV lytic cycle. EBV early genes are synergistically induced by ZTA and RTA and encode the viral polymerase and replication machinery. Late viral genes encode structural proteins that encapsidate and mediate release of infectious virions (McKenzie and El-Guindy, 2015).

mRNA expression profiling has provided important information on the kinetics of viral gene expression upon lytic cycle induction in Burkitt lymphoma cell lines (Koganti et al., 2015, Yuan et al., 2006). Likewise, RNA sequencing (RNA-seq) of lymphoblastoid cell lines with varying degrees of lytic replication provided insights into B cell and virus transcription patterns induced by EBV reactivation (Arvey et al., 2012). However, post-transcriptional effects may substantially alter the host and EBV proteome, and little is presently known about cell surface remodeling during EBV lytic replication. The comparative effects of type I and II EBV on human proteins are unknown.

We used tandem-mass-tag (TMT)-based MS3 mass spectrometry to perform quantitative temporal proteomic analysis of EBV replication in human Burkitt lymphoma B cells latently infected by type II EBV, prior to and at four time points after induction of lytic replication (Weekes et al., 2014). Selective plasma membrane (PM) protein enrichment enabled quantitation of global cell surface changes, without the need for specific antibodies. We quantified 8,318 host proteins, including 550 PM proteins and 69 EBV proteins, providing an in-depth temporal view of the host and viral proteome during B cell replication. Our analysis identified key host targets of EBV lytic replication, including multiple immune pathways. Unexpectedly, an EBV early factor targets the B cell receptor (BCR) complex for proteasomal degradation. We found that host protein abundance was similarly remodeled by type I EBV lytic replication in Burkitt lymphoma cells, identifying evolutionarily conserved EBV B cell targets. We further highlight host proteins co-targeted by multiple human herpesviruses and provide an in-depth resource for studies of EBV lytic replication.

## Results

### Quantitative Temporal Viromic Analysis of EBV Replication

**Figure 1:**
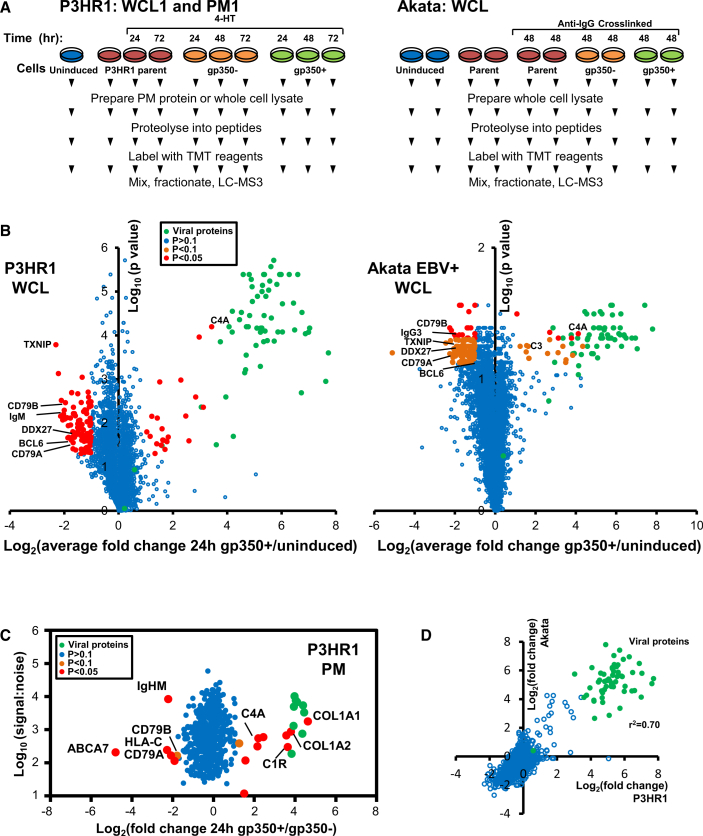
Temporal Proteomic Profiling of EBV B Cell Lytic Replication (A) Workflow of experiments WCL1 and PM1 in P3HR1 cells and a WCL experiment in Akata cells. Parental P3HR1 cells were analyzed at 0, 24, and 72 hr after 4-HT induction. P3HR1-ZHT/RHT cells were analyzed at 0, 24, 48, and 72 hr after 4-HT induction. Parental EBV-negative or EBV-positive Akata cells were analyzed at 0 and 48 hr after induction. Cells were sorted by FACS in order to separate lytic gp350^+^ cells from abortive lytic gp350^–^ cells. (B) Volcano plot of proteins quantified in all three P3HR1 WCL experiments (left panel) or the Akata WCL experiment (right panel). p values were estimated using a two-tailed t test (left panel) or a one-way ANOVA (right panel). In each case, values were corrected for multiple hypothesis testing using the method of Benjamini and Hochberg. Red dots: >2-fold change and p < 0.05. Orange dots: >2-fold change and p < 0.075. Green dots: viral proteins. (C) Scatterplot of PM proteins quantified in experiment PM1. Significance A was used to estimate p values (Cox and Mann, 2008), which were corrected using the method of Benjamini and Hochberg. (D) Correlation between average fold change at 24 hr (gp350^+^/uninduced) in P3HR1 cells (replicates WCL1–3) and average fold change (gp350^+^/uninduced) in Akata cell duplicates, for proteins quantified in all experiments.

We used the well-characterized type II EBV^+^ Burkitt lymphoma cell line P3HR1 to quantify changes in PM and whole cell lysate (WCL) protein expression by 10-plex TMT and MS3 mass spectrometry at three reference time points after induction of lytic EBV replication (experiments WCL1 and PM1, Figure 1A). Although it is difficult to induce viral replication in most latently infected B cell lines, P3HR1 is highly permissive for induced EBV lytic reactivation and exhibits low level spontaneous lytic replication (Balachandran et al., 1986, Verma et al., 2009). To ensure sufficient cells entered the EBV lytic replication cycle for proteomic analysis, the P3HR1 line was engineered to express the EBV immediate early proteins ZTA and RTA fused to a 4-hydroxy tamoxifen (4-HT)-dependent mutant estrogen receptor binding domain (ZHT and RHT, respectively) (Calderwood et al., 2008, Chiu et al., 2013). The addition of 4-HT causes ZHT/RHT nuclear translocation, triggering EBV lytic replication. As is commonly observed, abortive lytic replication occurred in a fraction of P3HR1 cells, in which ZTA/RTA do not trigger the full lytic program and infectious particles are not produced (Chiu and Sugden, 2016, Klein et al., 1974, Lin and Raab-Traub, 1987, Miller et al., 1974). We therefore used flow cytometry to sort 4-HT-induced P3HR1-ZHT/RHT cells into fully lytic and abortive lytic populations, based on expression of the EBV late cycle protein gp350, routinely achieving >40% of cells with PM gp350 expression (Figure 1A). We performed two additional replicate proteomic experiments at 24, 48, and 72 hr after induction of replication, which also enabled assessment of an early time point of infection (15 hr, experiment WCL2), or a full time course of analysis of mock 4-HT-induced parental control cell line, which lacks the ZHT/RHT system (experiment WCL3) (Figure S1A).

We quantified 8,249 human proteins across all three P3HR1 replicates (WCL1–3), including 6,307 proteins in all three replicates. We additionally quantified 69 EBV proteins (63 in all replicates). In experiment PM1, 550 PM proteins were quantified (Figures 1B, 1C, and S1B). We observed a high degree of correlation between biological replicates (Figure S1C).

To validate our results in a second Burkitt lymphoma cell system and to determine the extent to which proteome remodeling is conserved between type I and II EBV replication, we next analyzed Akata cells (Figure 1A). Type I EBV^+^ Akata cells are highly permissive for EBV lytic replication in response to anti-immunoglobulin G (IgG) cross-linking (Takada and Ono, 1989). We examined biological duplicates at 0 and 48 hr after Ig cross-linking and again used flow cytometry to sort induced cells into fully lytic and abortive lytic populations, based on EBV gp350 PM expression at 48 hr. To control for additional effects of anti-IgG stimulation, we performed parallel analyses of unstimulated and mock anti-IgG-induced EBV-negative Akata control cells (Figure 1A). These analyses quantified 7,041 proteins. Biological replicates clustered tightly (Figure S1D), and a comparison of changes in gp350^+^ Akata and P3HR1 cells revealed a high degree of correlation, with an r^2^ value of 0.70 (Figure 1D). All data are shown in Table S1, where the worksheet “Plotter” is interactive, enabling generation of temporal graphs of PM and WCL expression of any of the human and viral proteins quantified. Throughout this manuscript, all analyses are based on the average values from all analyzed replicates.

### Quantitative Temporal Viromics Identifies Down- or Upregulated Pathways

**Figure 2:**
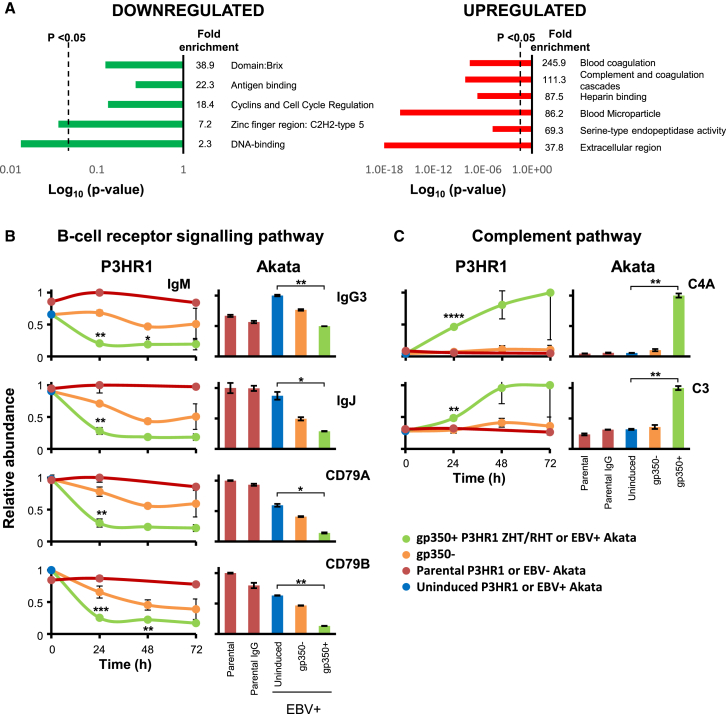
Enrichment Analysis Identifies EBV-Induced Downregulation of the BCR Complex and Upregulation of the Complement Pathway (A) Functional enrichment within all proteins that were significantly (p < 0.05) downregulated an average of >2-fold by lytic EBV replication in P3HR1 replicates WCL1–3 (red dots in Figure 1B, left panel). A background of all quantified proteins was used. A similar analysis was performed for proteins significantly upregulated >2-fold. Dotted lines: p = 0.05. Components of each cluster are shown in Tables S2A and S2B. (B) All BCR components quantified. Temporal plots (left-hand panels) illustrate data averaged over P3HR1 experiments WCL1–3. The parental cell line was measured only in experiments WCL1 and WCL3. Right-hand panels show corresponding results from Akata cells. Error bars ±SEM (triplicates) or range (duplicate). p values were calculated as described in Figure 1B: ^∗∗∗∗^p < 0.0005, ^∗∗∗^p < 0.005, ^∗∗^p < 0.05, ^∗^p < 0.075. (C) Upregulated complement components, illustrated as described in (B).

We used DAVID software (Huang et al., 2009) to identify pathways enriched among proteins significantly down- or upregulated during lytic EBV replication in WCL and PM samples from gp350^+^ P3HR1 or Akata cells. The term “antigen binding” or “B cell receptor signaling pathway” was enriched 14.7- to 22.3-fold among downregulated proteins compared to background (Figures 2A and S2A). Unexpectedly, the most highly downmodulated host factors included multiple BCR components: immunoglobulin M heavy chain (IgM) in P3HR1 and IgG in Akata, signaling chains CD79A and CD79B (also called Igα and Igβ), and the immunoglobulin joining chain (IgJ), which regulates multimerization of secretory IgM and IgA and is necessary for their poly-Ig receptor-mediated transfer across mucosal epithelium (Figures 1B, 1C, and 2B). Ig heavy-chain class switching was unlikely to account for the observed result, as we did not observe an accumulation of a different Ig heavy-chain isotype in either P3HR1 or Akata. Among significantly upregulated proteins, DAVID analysis identified the complement and blood coagulation pathways as highly significantly induced (Figures 2A, 2C, and S2A).

We additionally validated our proteomic findings by immunoblot for key innate immune signaling components AIM2, a cytosolic receptor for double stranded DNA with roles in sensing cytomegalovirus replication (Rathinam et al., 2010) and the transcription factors IRF1, IRF4, and STAT2, which each have important roles in antiviral responses (Figure S2B) (Blaszczyk et al., 2016, Taniguchi et al., 2001).

### An Early-Expressed EBV Protein Targets the BCR Complex for Degradation

**Figure 3:**
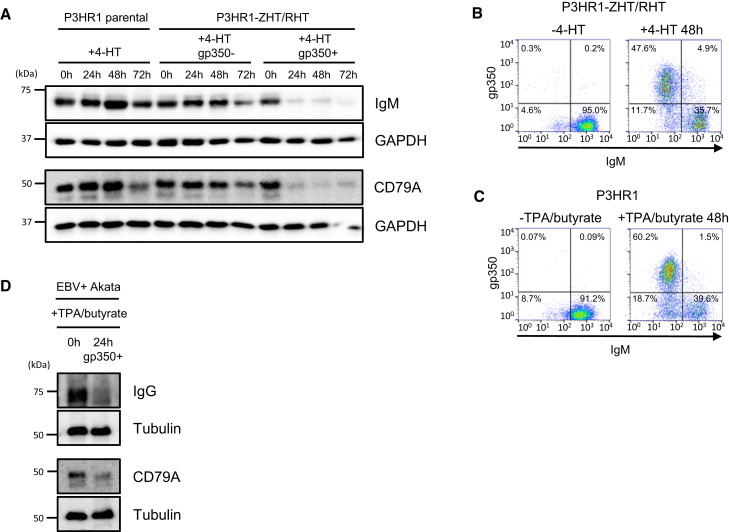
EBV Lytic Replication Targets the BCR Complex for Degradation (A) Immunoblot of whole-cell extracts indicate downregulation of IgM and CD79A in gp350^+^ ZHT/RHT cells, representative of three independent experiments. (B and C) Downregulation of IgM during P3HR1 lytic EBV replication confirmed by flow cytometry. Shown are representative plots for three independent experiments of uninduced (B; left) and 48-hr 4-HT-induced P3HR1 (B; right) or of uninduced (C; left) and 48 hr 12-O-Tetradecanoylphorbol-13-Acetate (TPA)/butyrate-induced P3HR1 (C; right). (D) EBV lytic replication destabilizes the IgG BCR complex. EBV^+^ Akata B cells were induced to undergo lytic replication by TPA/butyrate treatment for 24 hr. WCL from sorted gp350^+^ cells were immunoblotted are as indicated.

IgM, CD79A, CD79B, and J chain were all downregulated by 15 hr of EBV lytic induction in gp350^+^ P3HR1 cells (Table S1). We validated changes in IgM and CD79A by immunoblot. Moderate IgM and CD79A loss were observed in gp350^–^ cells by the 72-hr time point (Figure 3A), which may reflect downregulation by an early-expressed EBV protein during abortive replication in these cells. We additionally validated changes in IgM by flow cytometry, both by 4-HT induction in P3HR1-ZHT/RHT cells and chemical induction in parental P3HR1 cells (Figures 3B and 3C).

**Figure 4:**
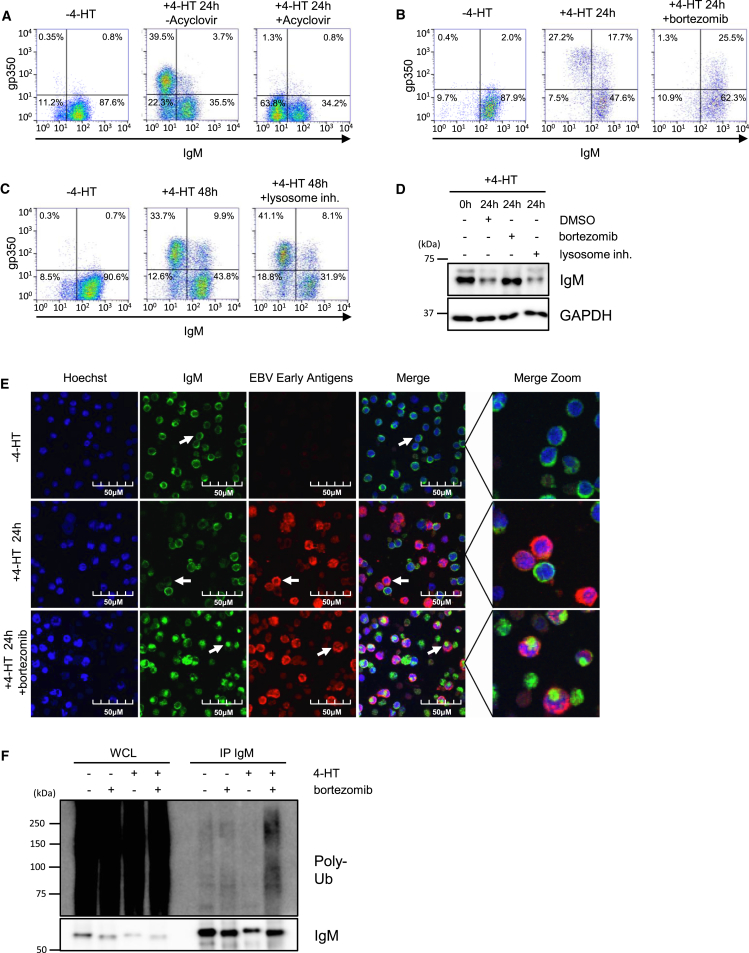
An EBV Early Factor Targets IgM for Proteasomal Degradation (A–C) Flow cytometry of P3HR1-ZHT/RHT cells, treated with acyclovir (A), bortezomib (B), or lysosome inhibitors (C), as indicated. Shown are representative plots for three independent experiments. (D) IgM or GAPDH immunoblot analysis of WCL from P3HR1-ZHT/RHT treated with bortezomib or lysosome inhibitors, representative of three independent experiments. (E) Immunofluorescence analysis of Hoechst stain, IgM, or EBV early antigen expression in P3HR1 ZHT/RHT cells treated with 4-HT and bortezomib, as indicated. Proteasome inhibition results in intracellular accumulation of IgM in early antigen^+^ cells. (F) Immunoblot analysis of poly-ubiquitin chains (poly-Ub) from WCL or IgM complexes immunopurified from P3HR1 ZHT/RHT cells, induced for EBV lytic replication for 24 hr, in the absence or presence of bortezomib, as indicated. Blots are representative of three independent experiments.

EBV lytic reactivation typically occurs in B cells expressing either IgM or class-switched IgG heavy chains. Induction of EBV reactivation in IgG^+^ Akata cells by anti-Ig cross-linking or by treatment with sodium butyrate led to robust downregulation of IgG and CD79A/B (Figures 2 and 3D). Acyclovir inhibits the viral polymerase, preventing EBV late gene expression (Colby et al., 1980, Feighny et al., 1981). Acyclovir treatment of 4-HT-induced P3HR1 cells did not appreciably affect IgM downregulation, even though it prevented expression of the late antigen gp350 (Figure 4A). These results are consistent with a model in which an early-expressed EBV gene, conserved between type I EBV (Akata) and type II EBV (P3HR1) strains, targets the BCR for degradation independently of heavy-chain isotype.

Lysosomal blockade had little effect on lytic replication-induced loss of IgM; however, inhibition of the proteasome by bortezomib stabilized IgM in gp350^+^ cells (Figures 4B–4D). By immunofluorescence, we similarly observed IgM loss in 4-HT-induced P3HR1-ZHT/RHT cells that was rescued by bortezomib, without appreciable effect on early antigen expression (Figure 4E).

IgM distribution appeared distinct in control versus bortezomib-treated 4-HT-induced cells, suggesting accumulation in an intracellular pool, for example a cytoplasmic aggresome (Figure 4E). To further test this possibility, we examined immunopurified P3HR1-ZHT/RHT IgM complexes from uninduced and induced cells. EBV lytic replication in the presence of bortezomib increased levels of poly-ubiquitin conjugates attached to immune-purified IgM complexes (Figure 4F). Taken together, our results suggest that an EBV early gene targets cell-surface and intracellular BCR pools for ubiquitin-mediated proteasomal degradation.

### EBV Lytic Replication Induces B Cell Complement Pathway Expression

Complement proteins C3, C4A, and C7 were substantially upregulated by EBV replication in P3HR1 and Akata cells, in addition to C5 and C9 in P3HR1 (Figure 2C; Table S1). Complement component expression was detectable by 24 hr after induction and peaked at 48–72 hr. Similarly, C1R, and C4A were upregulated at the PM of gp350^+^ but not gp350^–^ P3HR1 cells (Figure 1C). To our knowledge, complement expression has not previously been observed in B cells.

We excluded the possibility of an artifact due to deposition of serum complement proteins from cell culture media via qRT-PCR for C3 and C4A in gp350^+^ cells (Figure S2C). The classical pathway activator mannose activated serine protease 1 (MASP1) was similarly increased by EBV lytic replication (Figure S2C). Furthermore, polymorphisms in C3, C4A, and C5 protein sequences revealed that multiple identified peptides were of human and not of bovine origin (Table S3). These data indicate that EBV replication induces mRNA and protein expression of multiple complement components, and that complement components are deposited on lytic B cell PM. Complement induction may be a host response to EBV infection or may instead play an undefined role in EBV replication or spread, for example, through recently appreciated intracellular roles in host metabolism (Hess and Kemper, 2016). By contrast, lytic replication of the related herpesvirus human cytomegalovirus (HCMV) did not induce complement production in human fibroblasts (Weekes et al., 2014).

### EBV Lytic Program Effects on Host Cell-Cycle Regulators

**Figure 5:**
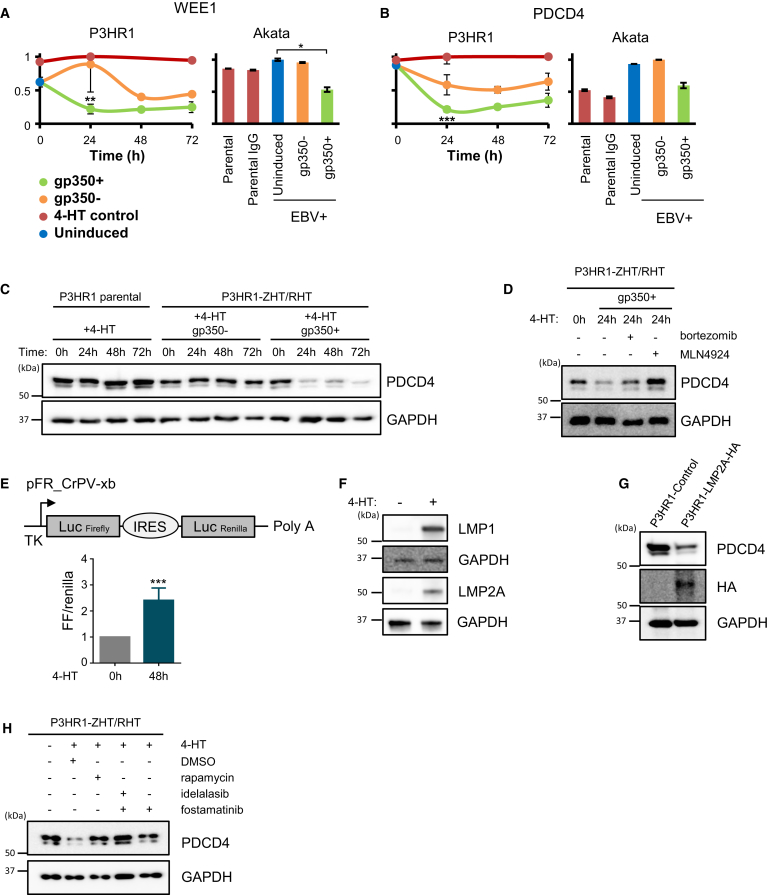
EBV Lytic Replication Suppresses Cell-Cycle Regulators WEE1 and PDCD4 and Increases Cap-Dependent Translation (A and B) Temporal proteomic analysis of WEE1 (A) and PDCD4 (B). ^∗∗∗^p < 0.01, ^∗∗^p < 0.05, ^∗^p = 0.1. (C and D) Immunoblots demonstrate that EBV lytic replication triggers PDCD4 protein loss (C), which is restored by proteasome antagonist bortezomib or neddylation antagonist MLN4924, representative of three independent experiments (D). (E) EBV lytic replication increases cap-dependent translation in P3HR1-ZHT/RHT cells. Cells were transiently transfected with the cap-dependent translation firefly (FF) and cap-independent renilla luciferase reporter vector pFR_CrPV-xb and rested for 24 hr. Cells were then left uninduced or were 4-HT-induced for 48 hr, and FF and renilla activity were quantified by dual luciferase assay. Shown are mean fold changes of renilla-normalized FF luciferase activity. Median and SEM values from three independent experiments. ^∗∗∗^p < 0.001. (F) LMP1 and LMP2A immunoblot of WCL from P3HR1 ZHT/RHT cells treated with 4-HT, as indicated. (G) LMP2A downregulates PDCD4. Immunoblot of WCL from uninduced P3HR1 cells stably expressing control or HA-tagged LMP2A is representative of three independent experiments. (H) Blockade of mTOR (rapamycin), PI3K (idelalasib), or SYK (fostamatinib) rescued cellular PDCD4 during EBV lytic replication. Immunoblot of sorted P3HR1 gp350^+^ cell WCL is as indicated. Blots are representative of three independent experiments.

The EBV lytic cycle promotes a pseudo-late G1/S-phase to support EBV replication, through partially understood mechanisms (Chiu et al., 2013, Hammerschmidt and Sugden, 2013, Kenney, 2007, Paladino et al., 2014). Enrichment analysis suggested that multiple cyclin-dependent kinases (CDK) or CDK regulators were downregulated during lytic replication (Figure 2A; Table S2A), including the cyclin-dependent kinase CDK4, which phosphorylates retinoblastoma family proteins to promote G1/S progression. A comprehensive search of our P3HR1 and Akata data revealed additional downmodulated cell-cycle regulators including tyrosine kinase WEE1 (Figure 5A), which inhibits entry into mitosis (Matheson et al., 2016); the licensing factor CDT1, which is a key component of the pre-replication complex and p15, which binds to CDK4 and inhibits G1/S progression (Havens and Walter, 2011). The observed effects on each of these key cell-cycle regulatory factors may contribute to establishment of the pseudo-late G1/S phase environment.

mTOR promotes cell-cycle progression through S6K and eIF4E pathways, though specific roles in EBV lytic replication remain incompletely characterized (Adamson et al., 2014). Interestingly, we observed the S6K target programmed cell death 4 (PDCD4) to be among the most highly downregulated protein by EBV replication in P3HR1 cells; a similar effect was observed in Akatas (Figures 5B and 5C). Since S6K-mediated phosphorylation triggers PDCD4 ubiquitination by the cullin ubiquitin ligase β-TRCP (Dorrello et al., 2006), the observed PDCD4 loss suggests that EBV lytic replication may activate the S6K pathway. We confirmed that PDCD4 was stabilized in 4-HT-induced P3HR1 cells by small molecule antagonists of proteasome or cullin ubiquitin ligase activity (Figure 5D). Since PDCD4 suppresses cap-dependent translation (Yang et al., 2003), we used cap-dependent firefly and cap-independent renilla luciferase reporters, revealing that EBV lytic induction significantly increased cap-dependent translation (Figure 5E).

EBV-encoded LMP2A is a dedicated activator of mTOR in latent EBV infection (Cen and Longnecker, 2015, Moody et al., 2005) that is also expressed in the viral lytic phase (Yuan et al., 2006), suggesting it may trigger PDCD4 loss. Interestingly, we found that despite the absence of EBNA2 in P3HR1, LMP1, and LMP2A expression were nonetheless robustly induced by 4-HT (Figure 5F). While EBNA2 drives LMP1 expression from the ED-L1 promoter in EBV latency III, EBV lytic replication may instead use the LT-R1 promoter located in the terminal repeats, which mediates LMP1/LMP2A expression in an EBNA2-independent manner (Kieff and Rickinson, 2007). Immunoblot analysis demonstrated that PDCD4 expression was markedly reduced by stable LMP2A expression in P3HR1 cells (Figure 5G). Likewise, inhibition of the LMP2A/mTOR pathway kinases SYK by fostamatinib or PI3K by idelalasib, or blockade of mTOR itself by rapamycin, each stabilized PDCD4 in 4-HT-induced P3HR1 cells (Figure 5H). Taken together, these results indicate that LMP2A supports EBV lytic replication by de-repressing PDCD4 effects on cap-dependent translation. PDCD4 downregulation may be of wider importance to other herpesviruses, as we previously found that HCMV also downregulates this protein (Weekes et al., 2014).

### Effects on B Cell Transcription Factor Networks

The physiological trigger for reactivation of latent EBV in B cells remains elusive; however, EBV replication is thought to initiate upon plasma cell differentiation (Laichalk and Thorley-Lawson, 2005). Interestingly, gene set enrichment analysis (GSEA) (Subramanian et al., 2005) identified enrichment of the plasma cell network in gp350^+^ P3HR1 cells (Figures S3A and S3B). Furthermore, DAVID analysis (Huang et al., 2009) suggested that EBV lytic replication downmodulated multiple B cell transcription factors (TFs) which are known to be suppressed upon germinal center B cell differentiation into plasma cells (Figure S4A; Table S3A). For instance, BCL6, which is critical for the maintenance of germinal center B cell fate, was decreased by ∼85% at 24 hr after induction of replication in P3HR1 and also significantly decreased in gp350^+^ Akata cells (Figure S4A). CRISPR knockout of the ubiquitin ligase FBXO11, which controls BCL6 steady-state levels in uninfected B cells (Duan et al., 2012), did not inhibit EBV-mediated BCL6 loss (Figure S4B). By contrast, BCL6 mRNA levels were significantly decreased by 24 hr after lytic induction by 4-HT (Figure S4C). These results suggest that EBV lytic replication blocks BCL6 expression prior to translation. Similarly, Myc is repressed during plasma cell terminal differentiation, and MYC levels were reduced by nearly 80% in gp350^+^ P3HR1 and Akata cells by 24 hr. ETS1 and ID3 each inhibit plasma cell differentiation (Nutt et al., 2015) and were similarly downregulated in gp350^+^ P3HR1 and Akata cells (Figure S4A). Notably, the deubiquitinase USP1 stabilizes ID3 (Williams et al., 2011), and USP1 was also suppressed by EBV lytic replication (Table S1). A transcription module composed of IRF8 and SPI1/PU.1 limits plasma cell differentiation (Nutt et al., 2015), and each was downmodulated in gp350^+^ P3HR1 and Akata cells by 24 hr (Figure S4A; Table S1).

We observed significant upregulation in gp350^+^ cells of TFs that have key roles in plasma cell development or terminal B cell differentiation including IRF4, the IRF4 target ZBTB20, Fos, and FosB (Table S1; Figure S4B) (Nutt et al., 2015). While plasma cell differentiation stimulates EBV lytic replication, our data suggest that EBV lytic replication in germinal-center-derived Burkitt lymphoma B cells may, in turn, remodel the B cell TF network to mimic key aspects of plasma cell differentiation.

### Identification of PM and Whole-Cell Proteins Commonly Targeted by Diverse Herpesviruses

**Figure 6:**
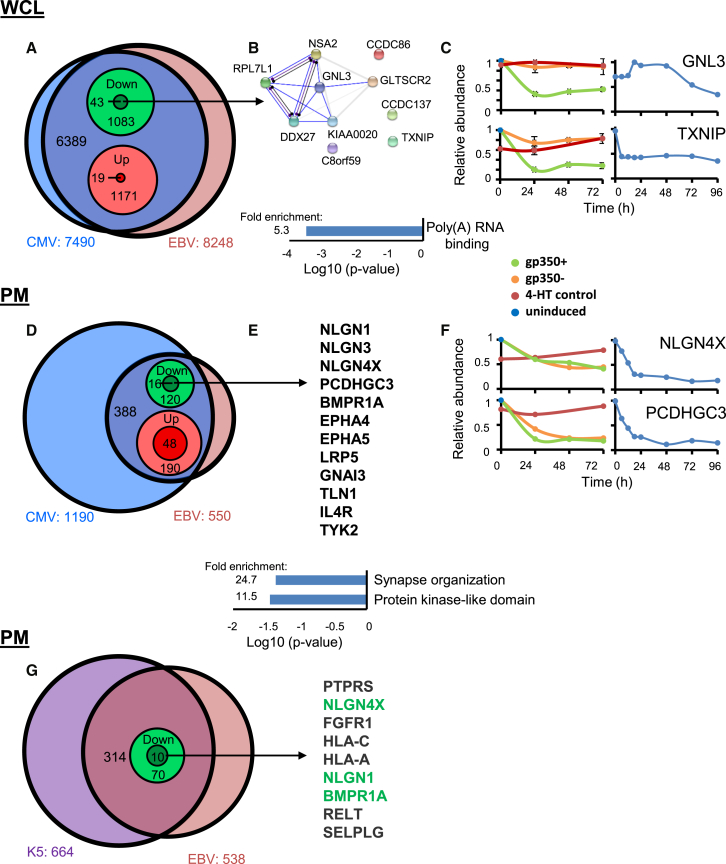
Whole-Cell and PM Proteins Co-regulated by EBV, CMV, and KSHV K5 (A) Overlap between EBV and CMV WCL data. 7,490 proteins were quantified in either CMV experiment WCL1 or WCL2 or both (Weekes et al., 2014). 6,389 of these proteins were additionally quantified in EBV experiments WCL1–3. From this overlap, 1,083 proteins were downregulated either by EBV or CMV, of which 43 were downregulated by both viruses. This initial “non-stringent” filtering included proteins downregulated in (1) all of experiments WCL1–3, (2) any combination of two of three experiments, but not quantified in the third, and (3) any one of three experiments, but not quantified in the other two. Using “stringent” filtering, we identified a subset of ten proteins downregulated in all three EBV and both CMV experiments, that were additionally downregulated at least 2-fold more than the P3HR1 parental control, to exclude non-specific effects of tamoxifen treatment (Figure 2 and Experimental Procedures). Of the 19 proteins upregulated by both viruses, only one (APOE) met our stringent criteria. (B) DAVID analysis identified eight of ten stringently filtered proteins functioning in poly(A) RNA binding (p < 0.001), of which six are known to interact (STRING database). Blue lines represent experimental evidence for physical binding, purple for catalysis, and black for reaction. Gray bars indicate evidence for co-expression. (C) Example plots of two of the ten proteins GNL3 and TXNIP. Left: EBV experiments WCL 1–3. Right: CMV Experiment WCL2. (D) Analysis of co-regulated PM proteins. We used similar non-stringent filter as detailed in (A), comparing EBV experiment PM1 with HCMV experiments PM1 and PM2. Stringent filtering excluded non-specific effects of tamoxifen treatment only, as there was no second temporal replicate for the EBV PM experiment. (E) 12/16 downregulated proteins met our stringent criteria, of which the top four listed functioned in synapse organization (p < 0.05). 46/48 upregulated proteins met our stringent criteria. (F) Temporal proteomic analysis of NLGN4X and PCDHGC3. Left: EBV experiments WCL 1–3. Right: CMV experiment WCL 2. (G) Coregulation of PM proteins by EBV and the KSHV K5 gene. Nine proteins met the stringent filter (D), of which three were additionally downregulated by CMV (green text) (E).

Certain key proteins important in innate antiviral immunity are known to be targeted by more than one, or sometimes multiple viruses (Schoggins et al., 2011, Schreiner and Wodrich, 2013). To identify proteins jointly targeted by EBV and other human herpesviruses, we combined our data with our previous analyses of either temporal HCMV infection or of cell-surface proteins targeted by the Kaposi’s-sarcoma-associated herpesvirus (KSHV) viral ubiquitin E3 ligase K5 (Timms et al., 2013, Weekes et al., 2014). We applied stringent filters to identify proteins that were (1) regulated >2-fold in all experiments by both viruses; (2) regulated by EBV as opposed to the 4-HT added to induce lytic-cycle replication (Figure 6). Of 6,389 whole-cell proteins quantified in both HCMV and EBV analyses, ten downregulated host factors met all criteria, of which eight function in poly(A) RNA binding and six are known to interact (Figures 6A–6C; Table S4).

A similar analysis of PM proteins downregulated by both EBV and HCMV identified 12 proteins, enriched in molecules functioning in synapse organization (Figures 6D–6F; Table S4). We previously identified HCMV-induced downregulation of multiple members of the protocadherin family and provided initial confirmatory evidence that members of this family are activating natural killer (NK) ligands (Weekes et al., 2014). Protocadherin γC3 was also downregulated during EBV infection (Figure 6E, Table S4), in addition to three Neuroligins (NLGN1, 3, and 4X) and Bone Morphogenetic Protein Receptor Type 1A (BMPR1A). NLGN1, NLGN4X, and BMPR1A were also targeted by KSHV K5 (Figure 6G; Table S4), suggesting that these molecules may be of particular importance in herpesviral pathogenesis. A recent report suggested the existence of an as-yet-unidentified DNAM-1 ligand of particular importance for killing early-lytically infected B cells, whereas late-lytic cells are highly resistant to killing (Williams et al., 2015). It is thus possible that one or some of the protocadherins and neuroligins fulfill this function.

By stringent criteria, 48 proteins were commonly upregulated by EBV and cytomegalovirus (CMV) (Figures 6D and S2; Table S4). DAVID software suggested that this group was particularly enriched in proteins with Interpro categories that included “Unfolded protein binding,” “Endoplasmic reticulum (ER) lumen,” “chaperone,” suggesting increased ER stress. Further studies are required to determine whether these changes reflect directly targeted molecules versus non-specific effects of herpesviral infection.

### Quantitative Analysis of the EBV Virome

Prior proteomic analyses of EBV lytic protein expression have identified 16 or 44 EBV proteins at single time points of viral reactivation (Koganti et al., 2015, Traylen et al., 2015). A particular benefit of our proteomic analysis is the opportunity not only to identify viral proteins without specific reagents, but additionally to quantify (1) their expression over time using TMT reporters and (2) the relative abundance of each protein.

**Figure 7:**
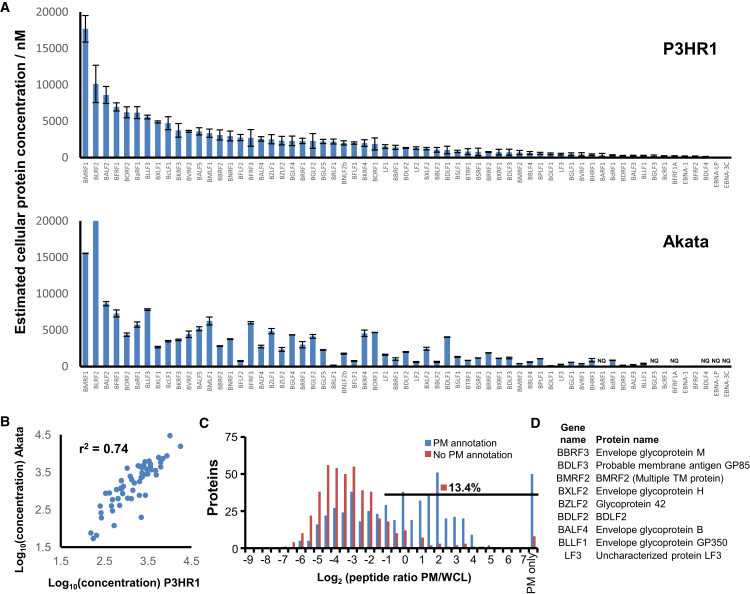
Characteristics of 63 Proteins (A) Estimated cellular concentration of all 63 viral proteins quantified in all three P3HR1 WCL samples (top panel). A “proteomic ruler” approach was used, which uses the mass spectrometry signal of histones to scale other proteins of unknown concentration (Wiśniewski et al., 2014). Protein concentrations were similarly calculated for both Akata duplicates (bottom panel). Error bars ±SEM (top panel); ± range (bottom panel). NQ, not quantified. The concentration of BLRF2 was estimated at 29,900 nM in Akata cells; however, the y axis extends only to 20,000 for ease of comparison with P3HR1 data. (B) Correlation between average protein concentrations estimated from P3HR1 and Akata WCL. (C) Identification of EBV proteins at the PM. Histogram of peptide ratios for all gene ontology (GO)-annotated proteins was quantified in experiment PM1. PM only, not detected in any of experiments WCL1–3. PM annotation, plasma membrane, cell surface, extracellular, or short GO. (D) Gene and protein names of all nine identified viral PM proteins.

We quantified 63 canonical EBV-encoded proteins in all three P3HR1 WCL samples. We used a “proteomic ruler” approach (Wiśniewski et al., 2014) to estimate cellular concentration (Figure 7A). We estimated that the most abundantly expressed EBV protein, DNA polymerase processivity factor BMRF1, was present at ∼1,000 times greater concentration than EBNA-3C, the least expressed protein. The top three most abundant proteins, BMRF1, BLRF2, and BALF2 accounted for 25% of the overall EBV viral protein abundance, and, in total, tegument proteins accounted for 39% of the total EBV protein abundance (Figure 7A). The least expressed EBV proteins that we detected included three Epstein-Barr nuclear antigens, which have roles in transcription regulation. Notably, the P3HR1 EBV strain does not encode the related EBNA2 TF and expresses a truncated form of EBNA-LP (Palser et al., 2015). 57/63 of these proteins were also quantified in Akata cells and displayed a similar relative abundance (Figure 7B).

To determine whether non-canonical EBV-encoded polypeptides were expressed in gp350^+^ cells, we searched our data against a six-frame translation of the P3HR1 genome (Palser et al., 2015). We identified only a single non-canonical EBV polypeptide, which was quantified by two peptides in experiment WCL1 and two peptides in WCL2. A further peptide from the same open reading frame (ORF) was identified in experiment WCL3; however, insufficient ions were present for quantitation (Figure S5A). DNA sequence encoding a similar ORF is present in the Akata genome (Grayson et al., 1985); however, peptides from this ORF were not identified in our Akata experiment. While additional EBV genes may exist in clinical isolates, our results are consistent with the hypothesis that the majority of P3HR1 strain EBV protein-coding genes have been identified.

We recently described a method of classification in which herpesviral proteins are grouped according to their temporal profiles (Weekes et al., 2014) and used this with data from experiment WCL2 to assign EBV proteins to four temporal classes (Figures S5B–S5D). The Tp1 cluster contains multiple members of the EBV viral preinitiation complex, which activates expression of most EBV late genes from newly replicated viral DNA (Aubry et al., 2014, Djavadian et al., 2016). The Tp2 cluster included proteins important in inhibition of host innate immunity, and multiple tegument, capsid, and envelope proteins were present in the later-expressed Tp3 class.

Abundant viral proteins present at the PM may be EBV vaccine and/or therapeutic antibody targets. We used a validated filtering strategy that relies on the ratio of PM to WCL peptides (Weekes et al., 2014) to identify nine high-confidence EBV PM proteins (Figures 7C and 7D; Experimental Procedures). These included four of five EBV glycoproteins involved in B cell entry: gp350, gp42 (encoded by BZLF2), gB, and gH (Hutt-Fletcher, 2015). The single-pass type I membrane protein BDLF3, implicated in cell surface major histocompatibility complex (MHC) class I and II downregulation (Quinn et al., 2015) and cell surface heparin sulfate binding (Chesnokova et al., 2016), was present at the P3HR1 PM. We additionally detected type II membrane protein BDLF2 and the multispanning membrane protein BMRF2, which form a complex implicated in epithelial attachment and possibly intercellular spread (Gore and Hutt-Fletcher, 2009, Hutt-Fletcher, 2015). Five EBV glycoproteins were not detected, including glycoprotein gL, the secreted factor BARF1, the tegument protein BLRF2, the 7-transmbrane segment G-protein-coupled receptor BILF1, and BILF2, whose function remains unknown. Additional membrane proteins may not have been detected given their high degree of hydrophobicity, including the multipass proteins LMP1 and LMP2A.

## Discussion

EBV establishes latency upon B cell infection. Global studies of EBV lytic reactivation have been limited by technical hurdles, such as the ability to obtain sufficient samples for systematic proteomic and viromic analysis. Most large-scale studies have therefore focused on mRNA transcription profiles (Concha et al., 2012, Koganti et al., 2015, Traylen et al., 2015, Yuan et al., 2006), single time-point analyses of a subset of the viral proteome, or on analyses of subsets of host proteins (Koganti et al., 2015, Traylen et al., 2015). Technological advances in mass spectrometry have enabled us now to provide the most complete to date temporal study of changes in the host proteome and EBV virome during lytic B cell replication. Collectively, our study provides a valuable resource for studies of EBV lytic replication and lays the foundation for future studies in ex vivo B cells, where limiting cell quantities preclude global proteomic analysis at present.

Our analysis unexpectedly revealed that an early EBV factor targets the BCR complex for proteasomal degradation. PM and WCL proteomic analysis, as well as fluorescence-activated cell sorting (FACS) and immunofluorescence, suggests that both PM and intracellular BCR pools are targeted. Further studies are required to identify whether soluble Ig isoforms are also targeted, and to characterize the ubiquitin-proteasome pathway by which EBV downmodulates these BCR pools. One candidate is EBV BDLF3, an early factor that targets cell-surface and intracellular MHC class I molecules for ubiquitin-mediated proteasome degradation (Quinn et al., 2015).

Targeting of the BCR during EBV B cell lytic replication may be conserved across EBV strains, since we observed a similar degree of Ig loss in Akata and P3HR1 B cells, which harbor type I and II EBV, respectively. We hypothesize that EBV targets the BCR to facilitate EBV B cell replication for one of the following reasons. First, a subset of lytic B cells may produce Ig reactive to antigen present on virions. BCR degradation would then enable EBV to avoid becoming trapped by binding to these antibodies within the secretory pathway or at the cell surface. Second, EBV lytic replication is thought to occur in tonsillar plasma cells, which are loaded with Igs. Plasma cells secrete Igs at the rate of approximately 2,000 molecules per second (Hibi and Dosch, 1986). Ig degradation may therefore shift metabolic capacity away from secretion of Ig and toward production of infectious virions, in order to prevent competition for amino acid resources. Third, multiple herpesviruses encode Ig Fc-binding proteins that enable lytic cells to evade antibody responses (Hook and Friedman, 2007). Degradation of endogenous Ig may prevent an EBV-encoded Fc receptor from being fully occupied by the Fc regions of plasma cell Ig, in particular, if EBV also downregulates soluble antibody.

Additional pathways were selectively downregulated during lytic viral reactivation, including multiple components of the cell-cycle machinery. Protein downregulation in some cases may involve the EBV host shutoff protein BGLF5, which was expressed early in infection (Table S1; Figure S5B). As BGLF5 accelerates host mRNA turnover (Rowe et al., 2007), it is likely either that BGLF5 selectively destabilizes these host mRNAs, or that targeted proteins are downregulated via an alternative route, as seen for the BCR complex. EBV-encoded microRNAs (miRNAs) also suppress expression of multiple host targets (Skalsky and Cullen, 2015). An important future goal will be to systematically compare changes in mRNA and protein abundances triggered by EBV lytic replication, in order to more fully define the extent to which EBV exerts post-transcriptional effects on host target gene expression.

Multiple aspects of the EBV life cycle are intimately linked with the complement system. The EBV entry protein gp350 has homology with the C3 complement component cleavage product C3dg (Nemerow et al., 1989). gp350 and C3dg each bind to CD21, a key B cell complement receptor. The Epstein-Barr virion accelerates decay of the alternative pathway C3 convertase (Mold et al., 1988). We now report that EBV B cell lytic reactivation stimulates complement component production. Despite its recognized roles in host defense, complement components are increasingly implicated in regulation of metabolism and cellular survival. Surprisingly, intracellular roles have recently been identified for complement, including in regulation of T cell metabolic networks and homeostatic survival (Hess and Kemper, 2016). Intracellular complement roles in BCL2 and Fas upregulation have been described in T cells (Kolev et al., 2015, Lalli et al., 2008). Similarly, KSHV latency proteins exploit the complement system to promote endothelial cell survival (Lee et al., 2014). Further studies will be required to determine whether complement upregulation represents a host defense to EBV, or EBV subversion of this immune pathway to activate metabolic or cell survival pathways that support B cell virion production.

EBV establishes latent infection upon B cell entry, and the tonsillar plasma cell population harbors lytic EBV in ex vivo studies. These observations support a model in which plasma cell differentiation provides a cue that stimulates EBV replication. Interestingly, our findings suggest that EBV lytic replication may likewise facilitate plasma cell differentiation, through suppression of TFs that maintain germinal center state and through upregulation of factors that promote plasma cell differentiation. Key germinal center B cell TFs downmodulated by EBV lytic replication included BCL6, ETS1, IRF8, ID3, and MYC, whereas the antibody secreting cell-inducing TFs IRF4, ZBTB20, FOS, and FosB were strongly upregulated. Thus, rather than passively activating as a bystander of plasma cell differentiation, EBV may play a more active role in remodeling the B cell environment to favor lytic replication.

To persist in an infected individual lifelong, herpesviruses have developed multiple strategies to modulate innate and adaptive immunity. One benefit is that we can use the overlap between proteins targeted by different herpesviruses to discover molecules particularly important in host defense. By comparing EBV, CMV, and KSHV, we identified downregulation of several members of the same protein family including Neuroligins and human leukocyte antigen (HLA) molecules. EBV and CMV both targeted Protocadherin γC3 for downregulation suggesting that this molecule, other members of the same family, and the Neuroligins might be activating NK ligands, as we previously demonstrated for Protocadherin FAT1 (Weekes et al., 2014).

The quantitation of ∼80% of canonical EBV proteins in a single experiment provides a significant technological advance and enabled systematic temporal analysis of γ-herpesviral protein expression. Clues as to viral mechanism may derive from correlation of viral and cellular protein expression patterns from our temporal analysis. Early-downregulated host proteins may be targeted by one of the earliest Tp1 or Tp2 classes of viral protein. Our protein temporal classes were complementary to classical IE/E/L nomenclature and provide an increased level of resolution in combination with transcriptional data. In general, we observed low-level expression of viral proteins in gp350^–^ cells compared to gp350^+^ cells, depending on the time point studied after induction (Figure S5C). Many of the host protein changes we observed were correspondingly regulated to a lesser extent in gp350^–^ cells (Figures 2B, S2B, and 5A; Table S1), suggesting that proteins expressed in abortive lytic P3HR1 or Akata cells likely account for these changes.

Innovative strategies are required to treat EBV-related diseases. Our quantitation of the relative abundance of cell surface EBV viral proteins may enable rational design of therapeutic monoclonal antibodies for preliminary studies and enhance efforts to develop an EBV vaccine. Collectively, our studies provide a rich resource for further analyses of EBV B cell lytic replication, and herpesviral infections more widely, and identify multiple viral targets for additional analysis.

## Experimental Procedures

Brief descriptions of key experimental procedures are provided below. For complete details, see Supplemental Experimental Procedures.

### WCL and PM Preparation

PM profiling was performed as previously described (Weekes et al., 2014) with minor modifications. FACS cells were washed twice with ice-cold PBS, followed by oxidation of sialic acid residues with sodium-meta-periodate. Plasma-membrane glycoproteins were selectively labeled with aminooxy-biotin. The reaction was quenched, cell numbers were normalized, and then cells were lysed in Triton X-100.

For whole-proteome samples, cells were washed, lysed in 6 M guanidine/50 mM HEPES buffer, and then vortexed, sonicated, and centrifuged. Protein concentrations were determined by bicinchoninic acid assay (BCA).

### Protein Isolation and Peptide Labeling with Tandem Mass Tags

TMT-based analysis was performed as described previously (Weekes et al., 2014). Alkylated and reduced proteins were digested into peptides, which were labeled with TMT reagents, and fractions generated from combined peptide samples strong cation exchange (PM samples) or high pH reverse-phase high-performance liquid chromatography (HPLC).

### Mass Spectrometry and Data Analysis

We performed mass spectrometry as described previously using an Orbitrap Fusion or Orbitrap Lumos (Weekes et al., 2014) and quantified TMT reporter ions from the MS3 scan. Peptides were identified and quantified using a Sequest-based in-house software pipeline. A combined database was searched, consisting of human, P3HR1 and Akata strains of EBV, all ORFs from six-frame translations of P3HR1 and Akata, and common contaminants. Peptide-spectral matches were filtered to a 1% false discovery rate (FDR) using linear discriminant analysis in conjunction with the target-decoy method (Huttlin et al., 2010). The resulting dataset was further collapsed to a final protein-level FDR of 1%. Protein assembly was guided by principles of parsimony. Where all peptide spectral matches (PSMs) from a given EBV protein could be explained either by a canonical gene or an ORF from the six-frame translation, the canonical gene was picked in preference. Proteins were quantified by summing TMT reporter ion counts across all matching PSM after filtering based on isolation specificity. Reverse and contaminant proteins were removed, and protein quantitation values were exported for normalization and further analysis in Excel.

### Contact for Reagent and Resource Sharing

Further information and reagent requests may be directed to the corresponding authors.

## Author Contributions

I.E., M.P.W., and B.E.G. designed the experiments. I.E., L.N., J.A.P., Y.N., and M.P.W. performed proteomics experiments; I.E., L.W.W., C.W.A., and C.J. performed biochemical experiments; and I.E., L.N., M.P.W., and B.E.G wrote the manuscript. L.N., L.S., J.A.P., S.P.G., and M.P.W. analyzed the proteomics data, and N.E.G. provided EBV genome sequence data and bioinformatic support. Y.M. and N.E.G. performed additional bioinformatic analyses; E.K. provided helpful scientific discussions; and I.E., L.N., S.P.G., M.P.W., E.K., and B.E.G. edited the manuscript. M.P.W. and B.E.G. supervised all research.

## Accession Numbers

The accession number for the mass spectrometry proteomics data reported in this paper is ProteomeXchange Consortium PRIDE: PXD006317 (Vizcaíno et al., 2016).

## Supplemental Information

Document S1. Supplemental Experimental Procedures and Figures S1–S5

Table S1. Interactive Plotter and Summary Table for All Proteomic Data, Related to Figure 1

Table S2. Enriched Clusters from DAVID Analysis of Down- or Upregulated Proteins and Their Protein Components, Related to Figures 2 and S2

Table S3. Multiple Identified Peptides from Samples WCL1–3 and PM1 Were of Human and Not of Bovine Origin, from Analysis of Known Polymorphisms in Protein Sequences, Related to Figures 2 and S2

Table S4. Shortlists of WCL and PM Proteins Co-downregulated by EBV and HCMV or EBV and KSHV K5 Protein, Related to Figure 6 A representative term is shown from each significantly enriched cluster.

Document S2. Article plus Supplemental Information
