# Supplementary material for: A Temporal Proteomic Map of Epstein-Barr Virus Lytic Replication in B Cells
Source: Cell Rep. 2017 May 16;19(7):1479–93. doi: 10.1016/j.celrep.2017.04.062 (PMC5446956; doi:10.1016/j.celrep.2017.04.062)
Supplement: Document S1. Supplemental Experimental Procedures and Figures S1–S5 [file mmc1.pdf]

Cell Reports, Volume *19*

Supplemental Information

A Temporal Proteomic Map of Epstein-Barr Virus Lytic Replication in B Cells

Ina Ersing, Luis Nobre, Liang Wei Wang, Lior Soday, Yijie Ma, Joao A. Paulo, Yohei Narita, Camille W. Ashbaugh, Chang Jiang, Nicholas E. Grayson, Elliott Kieff, Steven P. Gygi, Michael P. Weekes, and Benjamin E. Gewurz

Figure S1

A

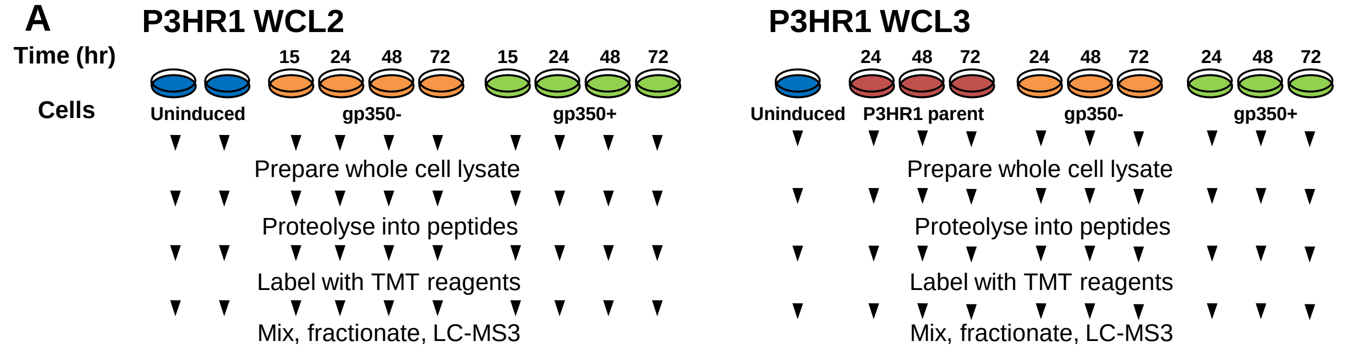

B

	P3HR1					Akata	P3HR1
	WCL1	WCL2	WCL3	Any WCL	All WCL	WCL	PM1
**All peptides**	89031	109497	95648			70311	5244
**Human proteins**	7493	7045	7382	8249	6307	6982	1012
**EBV proteins**	67	68	65	69	63	59	18

C

	1_P3HR1 parent Un	1_P3HR1 parent 24h	1_P3HR1 parent 72h	3_P3HR1 parent 24h	3_P3HR1 parent 48h	3_P3HR1 parent 72h	1_gp350- 24h	1_gp350- 48h	1_gp350- 72h	2_gp350- 15h	2_gp350- 24h	2_gp350- 48h	2_gp350- 72h	3_gp350- 24h	3_gp350- 48h	3_gp350- 72h	1_gp350+ 24h	1_gp350+ 48h	1_gp350+ 72h	2_gp350+ 15h	2_gp350+ 24h	2_gp350+ 48h	2_gp350+ 72h	3_gp350+ 24h	3_gp350+ 48h	3_gp350+ 72h
1_P3HR1 parent Un		0.85	0.83	0.50	0.44	0.49	0.35	0.17	0.15	0.05	0.07	0.04	-0.02	0.08	0.08	0.09	0.23	0.28	0.16	0.13	0.13	0.13	0.10	0.10	0.14	0.13
1_P3HR1 parent 24h	0.85		0.79	0.53	0.41	0.46	0.23	0.15	-0.02	0.12	0.09	0.04	0.04	0.02	0.00	0.07	0.15	0.15	0.05	0.12	0.11	0.12	0.12	0.00	0.07	0.07
1_P3HR1 parent 72h	0.83	0.79		0.50	0.51	0.50	0.19	0.01	0.09	0.05	0.02	0.00	0.01	0.00	-0.01	-0.03	0.08	0.14	0.05	0.04	0.04	0.06	0.07	0.01	0.05	0.04
3_P3HR1 parent 24h	0.50	0.53	0.50		0.78	0.90	-0.01	0.03	-0.11	0.05	0.01	-0.08	0.11	0.02	0.04	-0.08	-0.04	-0.03	-0.09	-0.01	-0.03	-0.03	0.05	0.00	0.01	0.04
3_P3HR1 parent 48h	0.44	0.41	0.51	0.78		0.85	0.11	0.09	0.09	0.07	0.08	0.13	0.27	0.24	0.31	0.13	0.17	0.19	0.20	0.19	0.18	0.20	0.24	0.30	0.31	0.27
3_P3HR1 parent 72h	0.49	0.46	0.50	0.90	0.85		0.10	0.12	0.04	0.07	0.05	-0.05	0.14	0.15	0.23	0.03	0.14	0.15	0.13	0.12	0.10	0.07	0.11	0.23	0.21	0.23
1_gp350- 24h	0.35	0.23	0.19	-0.01	0.11	0.10		0.66	0.67	0.37	0.34	0.27	0.21	0.39	0.52	0.41	0.75	0.75	0.73	0.55	0.56	0.46	0.35	0.48	0.47	0.55
1_gp350- 48h	0.17	0.15	0.01	0.03	0.09	0.12	0.66		0.54	0.16	0.20	0.15	0.25	0.33	0.45	0.37	0.59	0.65	0.61	0.35	0.35	0.31	0.28	0.41	0.41	0.48
1_gp350- 72h	0.15	-0.02	0.09	-0.11	0.09	0.04	0.67	0.54		0.00	0.11	0.20	0.09	0.40	0.52	0.48	0.51	0.57	0.75	0.23	0.26	0.23	0.17	0.39	0.39	0.41
2_gp350- 15h	0.05	0.12	0.05	0.05	0.07	0.07	0.37	0.16	0.00		0.67	0.52	0.53	0.24	0.26	0.15	0.37	0.26	0.20	0.73	0.70	0.60	0.51	0.27	0.26	0.29
2_gp350- 24h	0.07	0.09	0.02	0.01	0.08	0.05	0.34	0.20	0.11	0.67		0.48	0.48	0.30	0.32	0.25	0.37	0.31	0.26	0.64	0.60	0.59	0.44	0.30	0.32	0.30
2_gp350- 48h	0.04	0.04	0.00	-0.08	0.13	-0.05	0.27	0.15	0.20	0.52	0.48		0.54	0.61	0.56	0.62	0.29	0.28	0.26	0.58	0.66	0.80	0.68	0.34	0.45	0.25
2_gp350- 72h	-0.02	0.04	0.01	0.11	0.27	0.14	0.21	0.25	0.09	0.53	0.48	0.54		0.23	0.33	0.18	0.26	0.28	0.21	0.53	0.53	0.60	0.72	0.30	0.34	0.33
3_gp350- 24h	0.08	0.02	0.00	0.02	0.24	0.15	0.39	0.33	0.40	0.24	0.30	0.61	0.23		0.90	0.79	0.46	0.49	0.51	0.50	0.55	0.59	0.47	0.68	0.71	0.51
3_gp350- 48h	0.08	0.00	-0.01	0.04	0.31	0.23	0.52	0.45	0.52	0.26	0.32	0.56	0.33	0.90		0.78	0.63	0.64	0.69	0.60	0.63	0.64	0.54	0.83	0.85	0.71
3_gp350- 72h	0.09	0.07	-0.03	-0.08	0.13	0.03	0.41	0.37	0.48	0.15	0.25	0.62	0.18	0.79	0.78		0.35	0.38	0.44	0.35	0.41	0.49	0.38	0.48	0.56	0.40
1_gp350+ 24h	0.23	0.15	0.08	-0.04	0.17	0.14	0.75	0.59	0.51	0.37	0.37	0.29	0.26	0.46	0.63	0.35		0.92	0.89	0.76	0.75	0.65	0.47	0.80	0.74	0.82
1_gp350+ 48h	0.28	0.15	0.14	-0.03	0.19	0.15	0.75	0.65	0.57	0.26	0.31	0.28	0.28	0.49	0.64	0.38	0.92		0.90	0.69	0.69	0.63	0.51	0.79	0.77	0.83
1_gp350+ 72h	0.16	0.05	0.05	-0.09	0.20	0.13	0.73	0.61	0.75	0.20	0.26	0.26	0.21	0.51	0.69	0.44	0.89	0.90		0.62	0.63	0.54	0.41	0.78	0.73	0.80
2_gp350+ 15h	0.13	0.12	0.04	-0.01	0.19	0.12	0.55	0.35	0.23	0.73	0.64	0.58	0.53	0.50	0.60	0.35	0.76	0.69	0.62		0.96	0.88	0.72	0.72	0.70	0.70
2_gp350+ 24h	0.13	0.11	0.04	-0.03	0.18	0.10	0.56	0.35	0.26	0.70	0.60	0.66	0.53	0.55	0.63	0.41	0.75	0.69	0.63	0.96		0.91	0.73	0.71	0.70	0.68
2_gp350+ 48h	0.13	0.12	0.06	-0.03	0.20	0.07	0.46	0.31	0.23	0.60	0.59	0.80	0.60	0.59	0.64	0.49	0.65	0.63	0.54	0.88	0.91		0.83	0.66	0.73	0.62
2_gp350+ 72h	0.10	0.12	0.07	0.05	0.24	0.11	0.35	0.28	0.17	0.51	0.44	0.68	0.72	0.47	0.54	0.38	0.47	0.51	0.41	0.72	0.73	0.83		0.53	0.68	0.50
3_gp350+ 24h	0.10	0.00	0.01	0.00	0.30	0.23	0.48	0.41	0.39	0.27	0.30	0.34	0.30	0.68	0.83	0.48	0.80	0.79	0.78	0.72	0.71	0.66	0.53		0.93	0.90
3_gp350+ 48h	0.14	0.07	0.05	0.01	0.31	0.21	0.47	0.41	0.39	0.26	0.32	0.45	0.34	0.71	0.85	0.56	0.74	0.77	0.73	0.70	0.70	0.73	0.68	0.93		0.85
3_gp350+ 72h	0.13	0.07	0.04	0.04	0.27	0.23	0.55	0.48	0.41	0.29	0.30	0.25	0.33	0.51	0.71	0.40	0.82	0.83	0.80	0.70	0.68	0.62	0.50	0.90	0.85	

Pearson correlation

-0.11

0.96

D

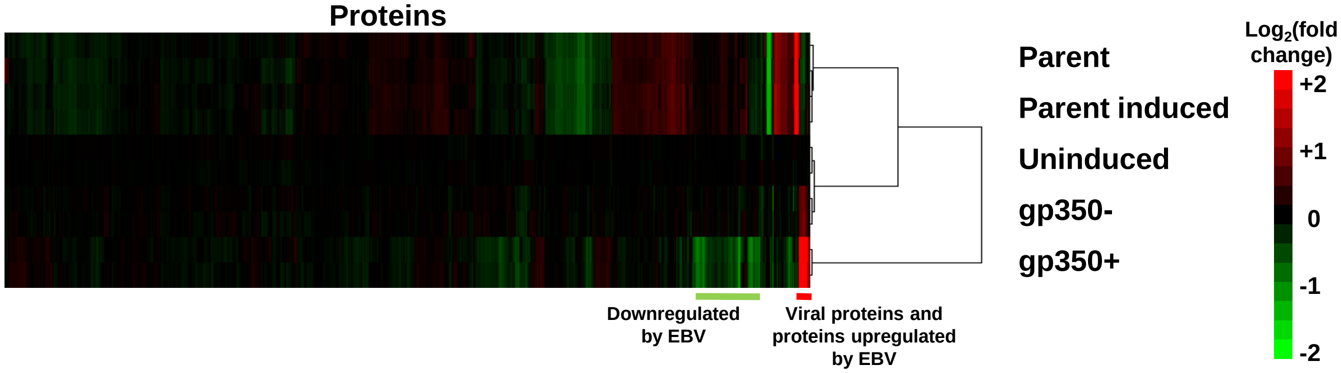

Figure S1. Related to **Figure 1**: Temporal proteomic profiling of EBV infection in B-cells.

A. Workflow of P3HR1 experiments WCL2 and WCL3. ZHT/RHT or parental control P3HR1 cells were treated with 4-HT for the indicated time.

B. Peptides and proteins quantified in all experiments in this manuscript. Of 1012 proteins quantified in P3HR1 experiment PM1, 550 were annotated by Gene Ontology 'plasma membrane', 'cell surface', 'extracellular' or 'short GO' (see Supplemental Experimental Procedures).

C. Matrix of Pearson correlations for P3HR1 data. For every sample in each of the P3HR1 WCL1-3 series, fold change was calculated compared to the unstimulated P3HR1-ZHT/RHT control (indicated in dark blue in Figures 1A, S1A) and then correlated to the fold change of every other sample. For the WCL2 series, the average of the two unstimulated controls was used in the calculation. Particularly strong correlations were seen between replicated gp350+ samples (lower right box) and replicate parental controls (upper left box).

D. Hierarchical cluster analysis of proteins quantified in the Akata WCL experiment (EBV-negative parent untreated or anti-IgG cross-linked for 48 hours, and EBV+ uninduced or gp350- versus gp350+ cells following 48 hour of anti-IgG crosslinking) demonstrated that biological duplicates clustered together. Fold change was calculated for each value compared to the average of the two uninduced samples. Proteins were filtered to be quantified by a minimum of 2 peptides.

Figure S2

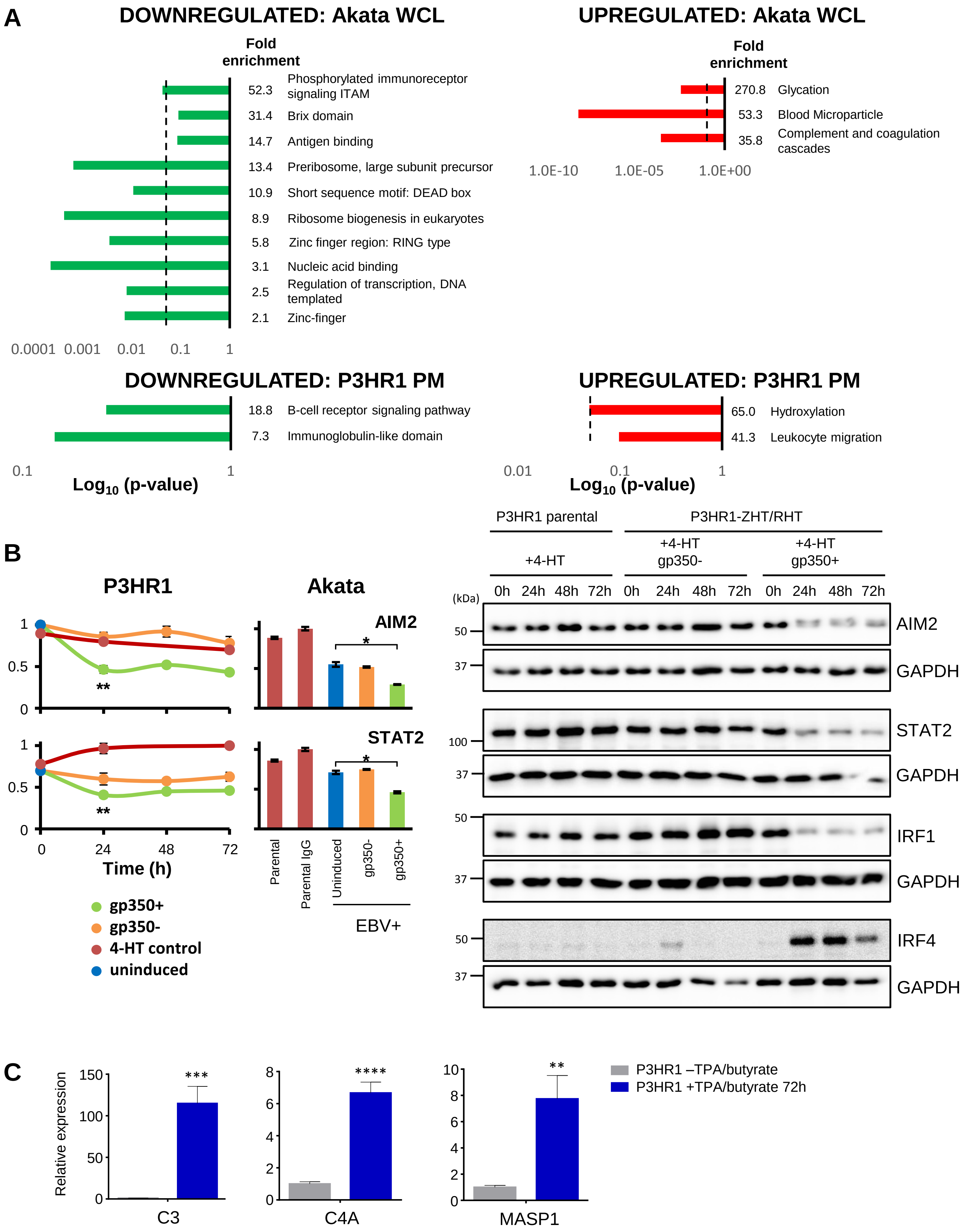
A
DOWNREGULATED: Akata WCL
Fold enrichment
52.3 Phosphorylated immunoreceptor signaling ITAM
31.4 Brix domain
14.7 Antigen binding
13.4 Preribosome, large subunit precursor
10.9 Short sequence motif: DEAD box
8.9 Ribosome biogenesis in eukaryotes
5.8 Zinc finger region: RING type
3.1 Nucleic acid binding
2.5 Regulation of transcription, DNA templated
2.1 Zinc-finger
0.0001 0.001 0.01 0.1 1
UPREGULATED: Akata WCL
Fold enrichment
270.8 Glycation
53.3 Blood Microparticle
35.8 Complement and coagulation cascades
1.0E-10 1.0E-05 1.0E+00
DOWNREGULATED: P3HR1 PM
18.8 B-cell receptor signaling pathway
7.3 Immunoglobulin-like domain
0.1 1
Log10 (p-value)
UPREGULATED: P3HR1 PM
65.0 Hydroxylation
41.3 Leukocyte migration
0.01 0.1 1
Log10 (p-value)
B
P3HR1
Akata
AIM2
STAT2
1
0.5
0
**
*
0 24 48 72
Time (h)
gp350+
gp350-
4-HT control
uninduced
Parental
Parental IgG
Uninduced
gp350-
gp350+
EBV+
P3HR1 parental
P3HR1-ZHT/RHT
+4-HT
+4-HT gp350-
+4-HT gp350+
(kDa)
0h 24h 48h 72h 0h 24h 48h 72h 0h 24h 48h 72h
50
37
100
AIM2
GAPDH
STAT2
IRF1
IRF4
C
Relative expression
150
100
50
0
***
****
**
8
6
4
2
10
C3
C4A
MASP1
P3HR1 –TPA/butyrate
P3HR1 +TPA/butyrate 72h

Figure S2. Related to **Figure 2**: EBV lytic replication induces complement pathway component expression in B-cells, and regulates components of the interferon pathway.

A. Functional enrichment within all proteins that were significantly ($p<0.075$) down or up-regulated an average of >2-fold by lytic EBV replication in Akata cells (red and orange dots in Figure 1B, right panel). A background of all quantified proteins was used. A similar analysis was performed for proteins down- or upregulated >2-fold in P3HR1 experiment PM1, with $p<0.1$ (red and orange dots in Figure 1C). Dotted lines: $p=0.05$.

B. Downregulation of AIM2 and STAT2 by EBV lytic replication, with validation by immunoblot. Also shown are immunoblots of P3HR1 whole cell extracts for IRF1 down-modulation and up-regulation of IRF4 (Table S1). STAT2 immunoblot was performed on the same membrane as CD79A in Figure 3A.

C. qRT-PCR identification of complement transcript upregulation in induced P3HR1 cells induced by TPA and sodium butyrate treatment. Shown are mean and SEM of three independent experiments. **$p<0.001$, ***$p<0.0001$, ****$p<0.00001$.

Figure S3

A

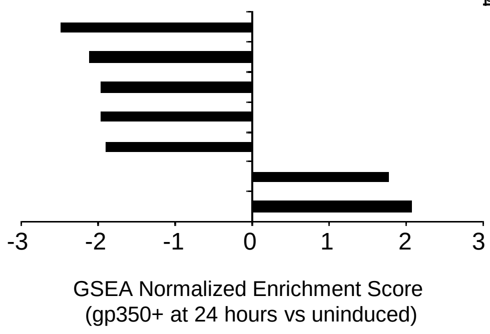

p-value	Direction of Gene Set Expression	
0.001	Naïve B-cell ↑	Plasma Cell ↓
0.002	Memory B-cell ↑	Blood Plasma Cell ↓
0.002	GC B cell ↑	Plasma Cell ↓
0.006	Naïve B-cell ↑	Plasma Cell ↓
0.01	Memory B cell ↑	BM Plasma Cell ↓
0.02	Plasmablast ↑	Naïve B-cell ↓
0.001	Blood Plasma Cell ↑	Naïve B-cell ↓

B

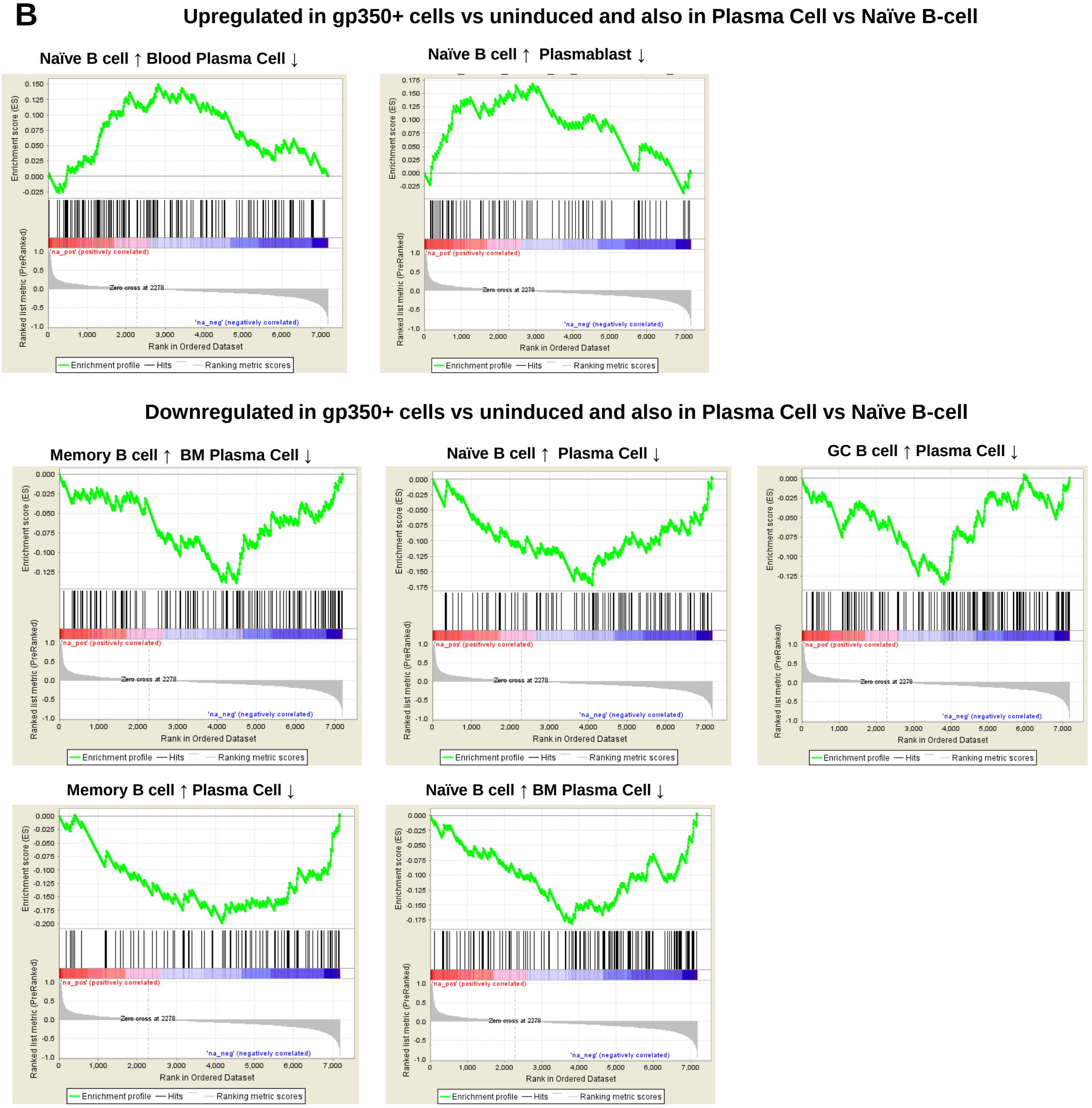

Figure S3. Related to **Figure 5**: Gene set enrichment analysis identified enrichment of plasmablast and plasma cell markers in gp350+ B-cells.

A. Changes in WCL composition between uninduced versus gp350+ P3HR1 cells (from the 24 hour timepoint) were analyzed by GSEA Preranked analysis. The differences in WCL3 proteome-wide abundances between uninduced and gp350+ WCL were cross-compared with published microarray B-cell differentiation datasets (Subramanian et al., 2005). Significantly enriched gene sets involving plasma cells were selected and visualized. Enrichment levels were expressed as Normal Enrichment Score (NES), calculated by the GSEA analysis. Significantly enriched gene were selected (p-values shown). GSEA demonstrated gp350+ cell enrichment for factors more highly expressed in plasmablasts and plasma cells, and depletion for factors more highly expressed in naïve or memory B-cells.

B. Data underlying the GSEA enrichment scores shown in (A).

Figure S4

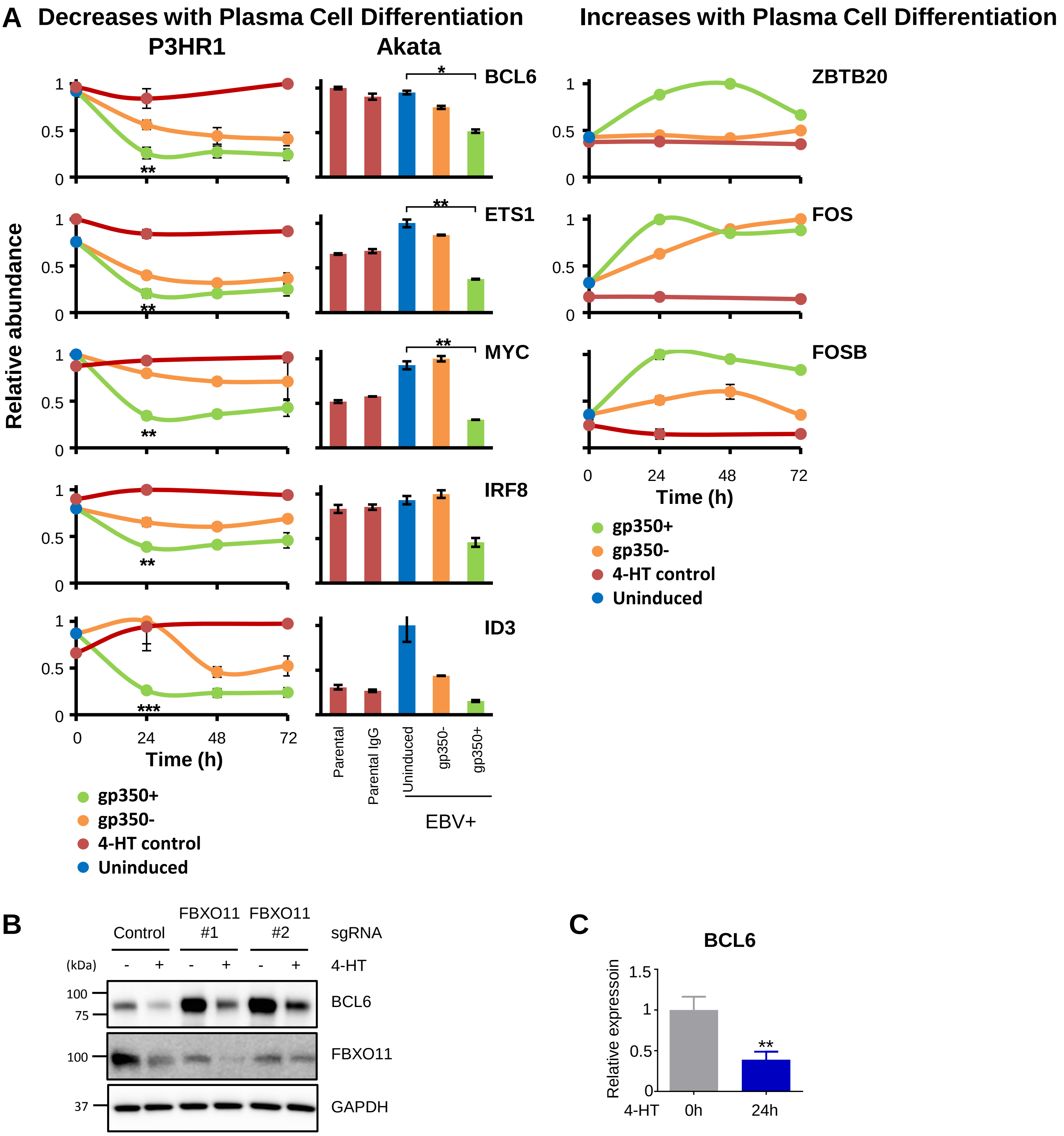
A
Decreases with Plasma Cell Differentiation
P3HR1
Akata
Increases with Plasma Cell Differentiation
BCL6
ETS1
MYC
IRF8
ID3
ZBTB20
FOS
FOSB
Relative abundance
Time (h)
Parental
Parental IgG
Uninduced
gp350-
gp350+
EBV+
gp350+
gp350-
4-HT control
Uninduced
B
Control
FBXO11 #1
FBXO11 #2
sgRNA
(kDa)
4-HT
BCL6
FBXO11
GAPDH
C
BCL6
Relative expressoin
4-HT
0h
24h

Figure S4. Related to **Figure 5**: EBV lytic replication remodels multiple B-cell transcription factors that regulate plasma cell differentiation.

A. Temporal profiles of representative transcription factors whose abundance decreases or increases both with EBV lytic replication and plasma cell differentiation. p-values were calculated as described in Figure 1B: *** $p<0.005$, ** $p<0.05$, *$p<0.075$. ZBTB20 and FOS were only quantified in P3HR1 replicate WCL1. FOSB was only quantified in P3HR1 cells, and not Akata cells.

B. EBV downregulates BCL6 in an FBXO11-independent manner. P3HR1-ZHT/RHT Cas9+ cells expressing control versus two independent single guide RNAs (sgRNAs) against the ubiquitin ligase FBXO11 were treated with 4-HT for 72 hours then lysates immunoblotted, as shown.

C. Downregulation of BCL6 transcripts after induction in P3HR1-ZHT/RHT cells by qRT-PCR. Shown are mean and SEM of three independent experiments. *** $P < 0.01$.

Figure S5

A

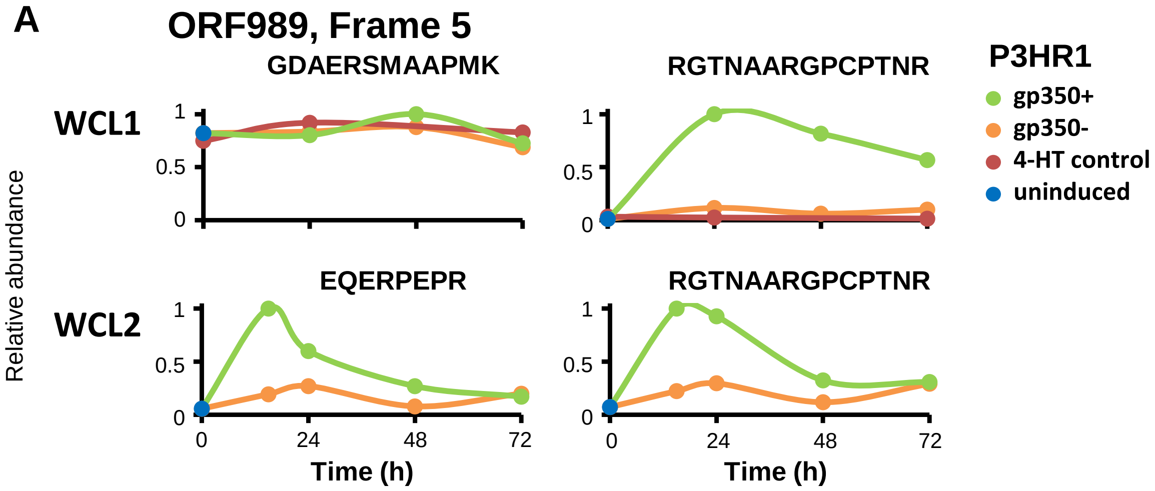

ORF989, Frame 5 (P3HR1 sequence)

FPERSGTYRTRQSHCIAHPCAGTRTRRRRLATPLTPEATKGDAERSMAAPMKVMASADTCPREQERPEPRGP
PGCLEHRGLQELPEATEQPEVPGQNLEG PAGAQGSRRGTNAARGPCPTNRGLT

ORF985, Frame 6 (Akata sequence)

FPERSGTYRTRRSRCIAHPCAGTRTRRRRLATPLTPEATKGDAERSMAAPMKVMASADTCPREQERPEPQGP
PGCLEHRGLQELPEATEQPEVPGQNLEGPAGAQGSRRGKNAARGPCPTNRGLT

B

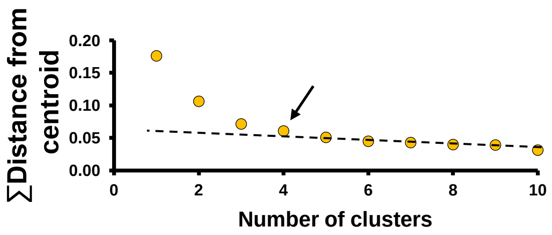

C

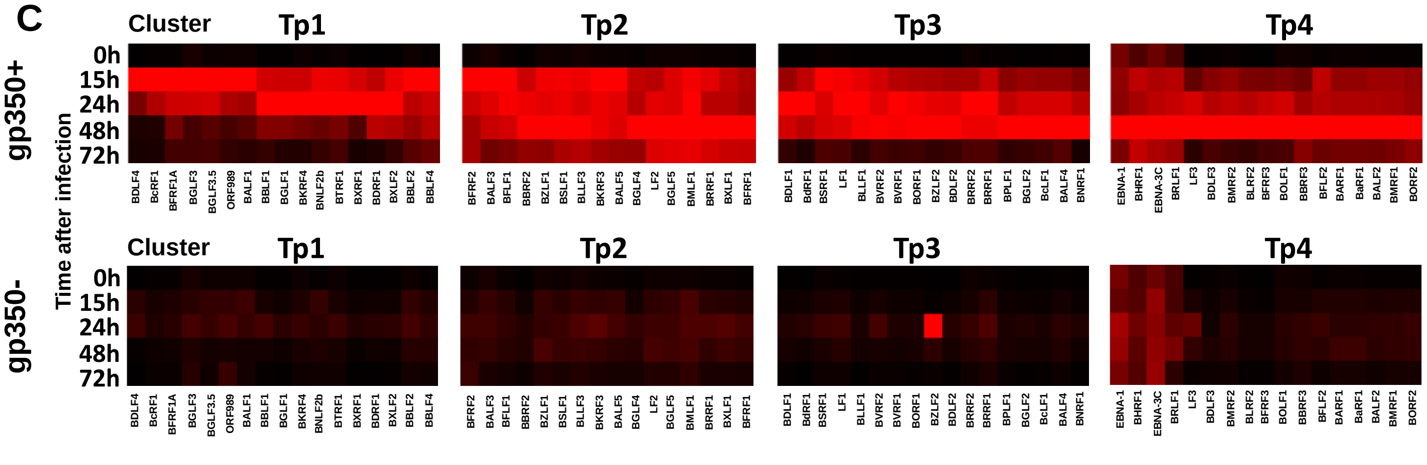

D

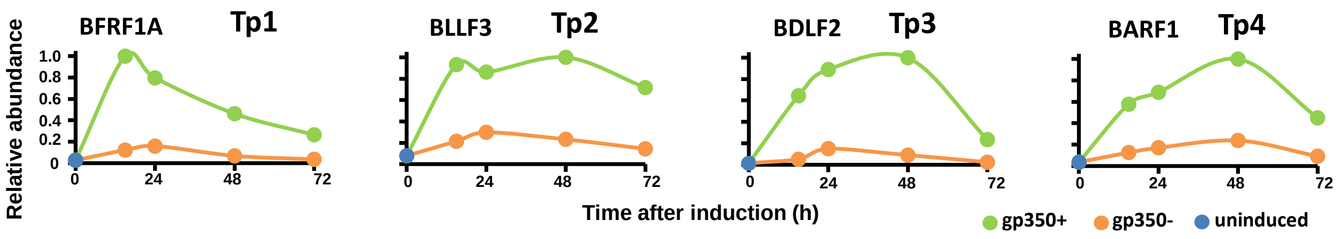

Figure S5, related to **Figure 7**. Peptides identified from two novel EBV ORFs.

A. Peptides quantified from novel ORF989, P3HR1 experiments WCL1 (upper panels) and WCL2 (lower panels). Note GDAERSMAAPMK may have been misidentified, and did not exhibit the expected temporal profile of a viral protein. Peptide GPCPTNR was identified in experiment WCL3, although insufficient ions were present for quantitation. Protein sequences of ORF989 are shown, indicating the position of the identified peptides both in the P3HR1 and corresponding Akata ORF sequences, although no peptides were quantified in our Akata experiment.

B. Number of temporal classes of EBV gene expression. We used the k-means approach with 1 to 10 classes to cluster viral proteins measured in experiment WCL2 (since this experiment included 4 rather than 3 time points of EBV replication) and assessed the summed distance of each protein from its cluster centroid. Although this summed distance necessarily becomes smaller as more clusters are added, the rate of decline decreases with each added group, eventually settling at a fairly constant rate of decline that reflects overfitting; clusters added prior to this point reflect underlying structure in the temporal protein data, whereas clusters subsequently added through overfitting are not informative. The point of inflexion fell between four and six classes, suggesting that even within sorted cells marked by gp350 late antigen expression, there are at least four distinct temporal protein profiles of viral protein expression. Profiles of proteins in each k-means cluster are shown in **Figure S5C-D**.

C. Temporal profiles of proteins in each k-means class were subjected to hierarchical clustering by Euclidian distance.

D. Temporal profiles of typical proteins from each cluster.

SUPPLEMENTAL EXPERIMENTAL PROCEDURES

Resources Table

REAGENT or RESOURCE	SOURCE	IDENTIFIER
Antibodies		
Anti-EBV gp350 (72A1)	BioXCell	N/A
Anti-IgM goat $F(ab')_2$	Southern Biotech	Cat# 2022-01
Anti-IgM-FITC goat	Southern Biotech	Cat# 2020-02
Anti-IgM-biotin goat	Southern Biotech	Cat# 2020-08
Anti-IgG goat $F(ab')_2$	Southern Biotech	Cat# 2042-01
Anti-EBV early antigens	Novus Biologicals	Cat# NBP2-34516
Anti-AIM2 rabbit mAb (D5X7K)	Cell Signaling Technologies	Cat# 12948
Anti-STAT2	Bethyl	Cat# A303-512A-T
Anti-IRF1 rabbit mAb (D5E4)	Cell Signaling Technologies	Cat# 8478
Anti-IRF4 rabbit Ab	Cell Signaling Technologies	Cat# 4964
Anti-CD79A rabbit mAb (D1X5C)	Cell Signaling Technologies	Cat# 13333
Anti-ubiquitin mouse mAb (P4D1)	Cell Signaling Technologies	Cat# 3936
Anti-PDCD4 rabbit mAb (D29C6)	Cell Signaling Technologies	Cat# 9535
Anti-BCL6 rabbit mAb (D412V)	Cell Signaling Technologies	Cat# 14895
Anti-α-Tubulin	Sigma	Cat# T5168
Anti-GAPDH mouse mAb	Proteintech	Cat# 60004-1-1g
Anti-HA.11	Covance	Cat# MMS-101P
Anti-FBXO11	Bethyl	Cat# A301-177A-T
AF488 goat anti-mouse	Molecular Probes	Cat# A-11029
AF568 goat anti-mouse	Molecular Probes	Cat# A-11031
HRP-conjugated anti-mouse	Cell Signaling Technologies	Cat# 7076
HRP-conjugated anti-rabbit	Cell Signaling Technologies	Cat# 7074
Anti-goat bovine IgG	Jackson Immunolabs	Cat# 805-035-180
Polyclonal rabbit anti-human IgG	Dako	Cat # A 0423
Biological Samples		
Chemicals, Peptides, and Recombinant Proteins		
Tandem mass tag (TMT) 10-plex isobaric reagents	Thermo Fisher	Cat# 90110
HPLC water	VWR	Cat# 23595.328
LC-MS grade Acetonitrile	Merck	Cat# 1.00029.2500
Formic acid	Thermo Fisher	Cat# 85178
Aminooxy-biotin	Biotium	Cat# 90113
Sodium meta-periodate	Thermo Fisher	Cat# 20504
Streptavidin agarose beads	Pierce	Cat# 20359

NeutrAvidin agarose beads	Thermo Fisher	Cat# 29200
Cy-5 fluorophore for 72A1 labeling	Abcam	Cat# ab188288
Bortezomib	Apex Bio	Cat# A2614
Idelalisib	Apex Bio	Cat# A3005
Fostamatinib	Apex Bio	Cat# A8332
Pepstatin A	Calbiochem	Cat# 516481
E-64 Protease Inhibitor	Calbiochem	Cat# 324890
Cathepsin Inhibitor I	Calbiochem	Cat# 219415
MLN4924	Active Biochem	Cat# A-1139
16% formaldehyde	Ted Pella Inc.	Cat# 9658705
Hoechst	Molecular Probes	Cat# 33258
Unless otherwise noted, all other chemicals	Sigma	
Complete Protease Inhibitor Cocktail	Roche	Cat# 11836153001
Critical Commercial Assays		
Dual Glo Luciferase Assay Kit	Promega	Cat# E2920
Power SYBR Green RNA-to-CT 1-Step Kit	Applied Biosystems	Cat# 4389986
BCA Protein Assay Kit	ThermoFisher Scientific	Cat# 23225
Deposited Data		
Raw Mass Spectrometry Data Files	This paper; Proteome Xchange	
Experimental Models: Cell Lines		
P3HR1 cl.16	(Rabson et al., 1983)	N/A
P3HR1-ZHT/RHT	(Calderwood et al., 2008)	N/A
Experimental Models: Organisms/Strains		
Recombinant DNA		
pFR_CrPV_xb	Addgene	#11509
lentiGuide-puro	Addgene	#52963
pHAGE-LMP2A	This paper	N/A
MSCV-GFP	Addgene	#41034
Sequence-Based Reagents		
Primer for sgFBXO11 #1 cloning 5'-CACCGATATCTTCTCCACAGCACCT-3'	IDT	N/A
Primer for sgFBXO11 #1 cloning 5'-AAACAGGTGCTGTGGAGAAGATATC-3'	IDT	N/A
Primer for sgFBXO11 #2 cloning 5'-CACCGTTTCACATCGAACCACTGTA-3'	IDT	N/A
Primer or sgFBXO11 #2 cloning 5'-AAACTACAGTGGTTCGATGTGAAAC-3'	IDT	N/A
Primer GAPDH for 5'-TGCACCACCAACTGCTTAGC-3'	IDT	N/A
Primer GAPDH rev 5'-GGCATGGACTGTGGTCATGAG-3'	IDT	N/A
Primer C3 for 5'-CCTGGACTGCTGCAACTACA-3'	IDT	N/A

Primer C3 rev 5'-GCAATGATGTCCTCATCCAG-3'	IDT	N/A
Primer C4A for 5'-GGAGAAGCTGAATATGGGCA-3'	IDT	N/A
Primer C4A rev 5'-GATGTGAGCTCTGCCTCCTC-3'	IDT	N/A
Primer MASP1 for 5'-CACTGTCCCAGATGGGTTTC-3'	IDT	N/A
Primer MASP1 rev 5'-GCACCTGGTCCTCAGTTTCT-3'	IDT	N/A
Primer BCL6 for 5'-GTTTCCGGCACCTTCAGACT-3'	IDT	N/A
Primer BCL6 rev 5'-CTGGCTTTTGTGACGGAAAT-3'	IDT	N/A
Software and Algorithms		
In-house mass spectrometry data analysis software	(Huttlin et al., 2010)	N/A
SEQUEST	(Eng et al., 1994)	N/A
XLStat	Addinsoft	N/A
STRING database	http://string-db.org/	N/A
DAVID software	https://david.ncifcrf.gov/	N/A
Cluster 3.0	Stanford University	N/A
Java Treeview	SourceForge.net	N/A
Other		
Orbitrap Fusion Mass Spectrometer	ThermoFisher Scientific	Cat# IQLAAEGAAP FADBMBCX
Orbitrap Fusion Lumos Mass Spectrometer	ThermoFisher Scientific	Cat# IQLAAEGAAP FADBMBHQ
Easy-nLC 1000	ThermoFisher Scientific	Cat# LC120

Cell lines

P3HR1, P3HR1-ZHT/RHT and EBV-positive Akata cells were cultured in RPMI (Thermo Fisher) supplemented with fetal bovine serum (10% v/v) and penicillin/streptomycin (Gibco) at 37°C in 5% CO_2. For selection, G418 (Gemini Bio-Products, P3HR1-ZHT/RHT, EBV-positive Akata cells) and hygromycin (Calbiochem, P3HR1-ZHT/RHT) were used. 293T cells were grown in DMEM, supplemented with fetal bovine serum (10% v/v) and penicillin/streptomycin (Gibco) at 37°C in 5% CO_2. All cells were confirmed to be mycoplasma negative.

Epstein-Barr virus reactivation

Epstein-Barr virus lytic replication cycle was reactivated in P3HR1-ZHT/RHT cells or mock-induced in parental P3HR1 cells by addition of 400 nM 4-HT (Sigma) to P3HR1-ZHT/RHT at a density of 3 x 10^5 cells/ml or by addition of 20 ng/ml phorbol 12-myristate 13-acetate (TPA) (Sigma). For later time points of induction, TPA/sodium butyrate was removed after 24h. For inductions in the presence of inhibitors, the following concentrations were used: bortezomib (100 nM), idelalisib (200 nM), fostamatinib (100 nM), pepstatin A (1 µg/ml), E-64 protease inhibitor (1 µM), cathepsin inhibitor (12.5 µM), MLN4924 (10 µM), acyclovir (50 µg/ml). EBV lytic replication was induced in Akata-EBV+ cells or mock-induced in parental Akata EBV-negative cells by addition of anti-human IgG (1:2000at a density of 5 x 10^5 cells/ml, as previously described (Takada and Ono, 1989). Where indicated, Akata cells were induced by addition of 3 mM sodium butyrate (Sigma).

Flow cytometry

For cell sorting, P3HR1-ZHT/RHT were washed once in PBS and incubated with the anti-gp350 primary mouse monoclonal antibody 72A1 on ice, followed by three washes in PBS/0.5% BSA and incubation with the secondary goat anti-mouse 488 antibody. For ZHT/RHT cells, flow sorting was performed on live cells on a BD FACSAria sorter, using cell surface gp350 expression. The parental clone P3HR1 cl.16 was mock induced with 4-hydroxytamoxifen or left uninduced, stained with PI (Molecular Probes) and FACS sorted, based on PI negativity. For intracellular staining of IgM and gp350, cells were fixed with 4% formaldehyde (Ted Pella, Inc.) and

permeabilized with 90% ice-cold methanol for 30 min. After one wash step in PBS/0.5% BSA, cells were incubated with anti-IgM-FITC, followed by incubation with anti-EBV-gp350 (72A1) on ice, as described above. Cells were washed in PBS twice before being analyzed on a FACSCalibur instrument. Data was processed using FlowJo software. For intracellular flow cytometry analysis, cells were fixed in 4% formaldehyde (Ted Pella, Inc.) and permeabilized with 90% ice-cold methanol for 30 min. Cells were incubated with primary antibody in PBS/0.5% BSA and secondary antibody on ice and analysed using a FACSCalibur instrument.

Immunoblot Analysis
Cell pellets were lysed SDS/Tris buffer and denatured at 95°C for 5 min. Samples were sonicated for 15 seconds and equal amounts of proteins were separated by SDS-PAGE using 10% or 12% Bis-Tris polyacrylamide gels. Samples were transferred to nitrocellulose membranes. Membranes were blocked in TBS/5% non-fat dried milk/0.2% Tween and incubated with indicated primary antibodies at 4°C overnight, washed in TBST three times for 10 min, followed by a 45 min incubation with HRP-conjugated secondary anti-mouse, anti-rabbit (Cell Signaling Technologies) or anti-goat (Jackson Immunolab) antibody, respectively. Blots were washed in TBST for 10 min, three cycles. Bands were visualized, using enhanced chemoluminescent substrate (PerkinElmer). Band intensities were measured using a Carestream Molecular Imaging workstation.

Immunoprecipitation
For immunoprecipitation, 3 x 10^7 cells were washed twice with PBS and lysed in 1.5 ml NP40 lysis buffer, supplemented with Complete Protease Inhibitor Cocktail (Roche), 10mM glycerophosphate, 2mM sodium pyrophosphate, 1mM PMSF and 10 mM N-Ethylmaleimide (NEM) for 15 min. All steps were performed on ice or at 4°C. Nuclear debris was removed by centrifugation at 13,000 x g for 15 min. Lysates were incubated with 10 µg anti-IgM-biotin antibody (Southern Biotech) for 1h, followed by incubation with 20 µl NeutrAvidin beads (Thermo Fisher) for 1h. After four washes in NP40 lysis buffer, supplemented with Complete Protease Inhibitor Cocktail (Roche), 10mM glycerophosphate, 2mM sodium pyrophosphate, 1mM PMSF and 2mM NEM, samples were eluted in SDS sample buffer. SDS-PAGE and immunoblot were performed as described above.

Immunofluoresence Microscopy
P3HR1 cells were dried onto slides and fixed with 4% formaldehyde (Ted Pella, Inc.). Cells were then permeabilized with 0.5% triton X-100 for 10 min and blocked with PBS/1% BSA. Cells were stained with primary antibodies, anti-mouse AF568 sequentially for 1h in PBS/1% BSA and Hoechst for 10 min in PBS. Washes were performed with PBS/0.1% Tween-20. For analysis by confocal microscopy, cover glasses were mounted onto slides with ProLong Gold Antifade Mountant (Invitrogen) and images were taken using a Olympus Fluoview FV1000 confocal microscope.

Lentiviral transductions in 293T cells
293T cells were transfected in 6 well plates with 500ng of lentiviral plasmid, together with 500ng gag-pol and 50ng VSV-G for packaging. P3HR1 cells were transduced with 2ml lenti-/retrovirus, produced in 293T cells, two times and put under selection for 48h. For stable GFP expression, we used MSCV-N-Flag-HA-GFP, a gift from Wade Harper (Addgene plasmid # 41034) (Sowa et al., 2009).

Lentiviral vectors for sgRNA expression
For CRISPR/Cas9-mediated knockout of FBXO11 in P3HR1 cells, we used the FBXO11-specific sgRNAs, cloned into pLentiguide-puro, a gift from Feng Zhang (Addgene plasmid # 52963) (Sanjana et al., 2014) and sequence verified (Eton Bioscience).

Luciferase-based cap-dependent translation assay
5 x 10^6 P3HR1-ZHT/RHT cells were electroporated with 25 µg pFR_CrPv_xb (Petersen et al., 2006), a gift from Phil Sharp (Addgene plasmid # 11509) using a BioRad GenePulser (200V, 0.95 µF). After resting the cells for 24h, viral replication was induced by addition of 4-HT to the media. Subsequent luciferase assays were performed using a Dual Glo Luciferase Assay kit (Promega), according to the manifacturer's instructions. After 48h, 5 x 10^5 cells were lysed in 100 µl lysis buffer. Cap-dependent firefly (FF) luciferase and cap-independent renilla luciferase activity was quantified on a Molecular Devices plate reader. Firefly and Renilla luciferase were normalized to levels in uninduced cells and fold changes of normalized firefly luciferase activity, relative to normalized Renilla activity were calculated.

Quantitative reverse transcription PCR

RT-qPCR was performed on a BioRad CFX Connect Real-time PCR detection system, using Power SYBR Green RNA-to-CT 1-Step Kit (Applied Biosystems) for 40 cycles. Expression values and SEM relative to GAPDH expression were calculated using CFX Manager Software.

TMT-based proteomics

Plasma membrane profiling was performed as described previously, (Weekes et al., 2012; Weekes et al., 2014). Briefly, FACS sorted B-cells were washed twice with ice-cold PBS. Sialic acid residues were oxidized with sodium meta-periodate (Thermo Fisher) then biotinylated with aminooxy-biotin (Biotium). The reaction was quenched, cell numbers for each condition were normalized to 2×10^6 using a BioRad TC20 automated cell counter and the biotinylated cells were lysed in 1.6% Triton X-100 lysis buffer. Biotinylated glycoproteins were enriched with high affinity streptavidin agarose beads (Pierce) and washed extensively. Captured protein was denatured with DTT, alkylated with iodoacetamide (IAA, Sigma) and digested on-bead with trypsin (Promega) in 200 mM HEPES pH 8.5 for 3h. Tryptic peptides were collected.

For whole proteome samples, cells were washed twice with PBS, and 150 ul of 6M Guanidine/50 mM HEPES pH8.5 lysis buffer added. Samples were vortexed extensively then sonicated. Cell debris was removed by centrifuging at 13,000 g for 10 min twice. Dithiothreitol (DTT) was added to a final concentration of 5mM and samples were incubated for 20 min. Cysteines were alkylated with 15mM iodoacetamide and incubated 20 min at room temperature in the dark. Excess iodoacetamide was quenched with DTT for 15 min. Samples were diluted with 200 mM HEPES pH 8.5 to 1.5 M Guanidine followed by digestion at room temperature for 3 hr with LysC protease at a 1:100 protease-to protein ratio. Trypsin was then added at a 1:100 protease-to-protein ratio followed by overnight incubation at 37oC. The reaction was quenched with 1% formic acid, samples were spun at 21,000g for 10 min to remove debris and undigested protein, then subjected to C18 solid-phase extraction (Sep-Pak, Waters) and vacuum centrifuged to near-dryness.

In preparation for TMT labelling, desalted peptides were dissolved in 200 mM HEPES pH 8.5. For whole proteome samples, peptide concentration was measured by micro BCA (Pierce), and 50 mg of peptide labelled with TMT reagent. For plasma membrane samples, 100% of each peptide sample was labelled. TMT reagents (0.8 mg) were dissolved in 43 μL anhydrous acetonitrile and 5 μl added to peptide sample at a final acetonitrile concentration of 30% (v/v). Samples were labelled as follows: Experiment WCL1 and PM1: P3HR1-ZHT/RHT uninduced (TMT 126); P3HR1-ZHT/RHT induced 24h gp350- (TMT 127N); P3HR1-ZHT/RHT induced 48h gp350- (TMT 127C); P3HR1-ZHT/RHT induced 72h gp350- (TMT 128N); P3HR1-ZHT/RHT induced 24h gp350+ (TMT 128C); P3HR1-ZHT/RHT induced 48h gp350+ (TMT 129N); P3HR1-ZHT/RHT induced 72h gp350+ (TMT 129C); P3HR1 (TMT 130N); P3HR1+4-HT 24h (TMT 130C); P3HR1+4-HT 72h (TMT 131). For Experiment WCL2: P3HR1-ZHT/RHT uninduced (TMT 126); P3HR1-ZHT/RHT induced 15h gp350+ (TMT 127N); P3HR1-ZHT/RHT induced 15h gp350- (TMT 127C); P3HR1-ZHT/RHT induced 24h gp350+ (TMT 128N); P3HR1-ZHT/RHT induced 15h gp350- (TMT 128C); P3HR1-ZHT/RHT induced 48h gp350+ (TMT 129N); P3HR1-ZHT/RHT induced 48h gp350- (TMT 129C); P3HR1-ZHT/RHT induced 72h gp350+ (TMT 130N); P3HR1-ZHT/RHT induced 72h gp350- (TMT 130C); P3HR1-ZHT/RHT uninduced, biological replicate (TMT 131). Experiment WCL3: P3HR1-ZHT/RHT uninduced (TMT 126); P3HR1+4-HT 24h (TMT 127N); P3HR1-ZHT/RHT induced 24h gp350- (TMT 127C); P3HR1-ZHT/RHT induced 24h gp350+ (TMT 128N); P3HR1+4-HT 48h (TMT 128C); P3HR1-ZHT/RHT induced 48h gp350- (TMT 129N); P3HR1-ZHT/RHT induced 48h gp350+ (TMT 129C); P3HR1+4-HT 72h (TMT 130N); P3HR1-ZHT/RHT induced 72h gp350- (TMT 130C); P3HR1-ZHT/RHT induced 72h gp350+ (TMT 131). Akata WCL experiment: EBV negative untreated duplicates (TMT 126 and 127N); EBV positive uninduced (TMT 127C and 128N); EBV positive mock induced (TMT 128C and 129N); EBV positive gp350 negative (TMT 129C and 130N); EBV positive gp350 positive (TMT 130C and 131).
Following incubation at room temperature for 1 hr, the reaction was quenched with hydroxylamine to a final concentration of 0.5% (v/v). TMT-labeled samples were combined at a 1:1:1:1:1:1:1:1:1:1 ratio. The sample was vacuum-centrifuged to near dryness and subjected to C18 solid-phase extraction (SPE) (Sep-Pak, Waters).
Offline high pH reversed-phase fractionation of peptides from all WCL experiments and offline tip-based strong cation exchange fractionation of the PM sample were performed, and WCL peptide fractions combined as described previously (Weekes et al., 2014).

Liquid Chromatography and Tandem Mass Spectrometry

Mass spectrometry data were acquired using an Orbitrap Lumos (experiments WCL1, PM1, Akata WCL) coupled with a Proxeon EASY-nLC 1000 LC pump (Thermo Fisher Scientific, San Jose, CA), or an Orbitrap Fusion (experiments WCL2-3). We quantified TMT reporter ions from the MS3 scan (McAlister et al., 2012; Ting et al., 2011).

For Orbitrap Lumos experiments, peptides were separated on a 75 mm inner diameter microcapillary column packed with 0.5 cm of Magic C4 resin (5 mm, 100 A°,Michrom Bioresources) followed by approximately 20 cm of GP118 resin (1.8 mm, 120 A°, Sepax Technologies). Peptides were separated using a 3 hr gradient of 6 to 30% acetonitrile in 0.125% formic acid at a flow rate of 300 nl/min. Each analysis used an MS3-based TMT method (McAlister et al., 2012; Ting et al., 2011). The scan sequence began with an MS1 spectrum (Orbitrap analysis, resolution 120,000, 350-1400 Th, AGC target 5 x 10^5, maximum injection time 100 ms). 'Rapid' was selected for MS2 analysis, which consisted of CID (quadrupole ion trap analysis, AGC 1.8 x 10^4, NCE 35, maximum injection time 120 ms). For MS3 analysis, precursors were fragmented by HCD prior to Orbitrap analysis (NCE 55, max AGC 3 x 10^5, maximum injection time 120 ms, isolation specificity 0.5 Th, resolution 60,000).

For Orbitrap Fusion experiments, peptides were separated as above. The scan sequence began with an MS1 spectrum (Orbitrap analysis, resolution 120,000, 400-1400 Th, AGC target 2 x 10^5, maximum injection time 100 ms). 'Rapid' was selected for MS2 analysis, which consisted of CID (quadrupole ion trap analysis, AGC 4 x 10^3, NCE 35, maximum injection time 150 ms). The top ten precursors were selected for MS3 analysis, in which precursors were fragmented by HCD prior to Orbitrap analysis (NCE 55, max AGC 5 x 10^4, maximum injection time 150 ms, isolation specificity 0.5 Th, resolution 60,000) (McAlister et al., 2012).

Data Analysis

Mass spectra were processed using a Sequest-based in-house software pipeline as described previously (Weekes et al., 2014). Briefly, MS spectra were converted to mzXML using a modified version of ReAdW.exe. A combined database was constructed from (a) a combined human Uniprot and Trembl database (February 4th, 2014), (b) P3HR1 strain EBV, (c) Akata strain EBV (d) all open reading frames from a six-frame translation of P3HR1 strain EBV, (e) all open reading frames from a six-frame translation of Akata strain EBV and (f) common contaminants such as porcine trypsin and endoproteinase LysC. The combined database was concatenated with a reverse database composed of all protein sequences in reversed order. Searches were performed using a 20 ppm precursor ion tolerance. Product ion tolerance was set to 0.03 Th. TMT tags on lysine residues and peptide N termini (229.162932 Da) and carbamidomethylation of cysteine residues (57.02146 Da) were set as static modifications, while oxidation of methionine residues (15.99492 Da) was set as a variable modification. To control the fraction of erroneous protein identifications, we used a target-decoy strategy (Elias and Gygi, 2007, 2010). Peptide spectral matches (PSMs) were filtered to an initial peptide-level false discovery rate (FDR) of 1% with subsequent filtering to attain a final protein-level FDR of 1%. PSM filtering was performed using a linear discriminant analysis, as described previously (Huttlin et al., 2010), considering the following parameters: XCorr, DCn, missed cleavages, peptide length, charge state, and precursor mass accuracy. Protein assembly was guided by principles of parsimony to produce the smallest set of proteins necessary to account for all observed peptides. Where all PSMs from a given EBV protein could be explained either by a canonical gene or an ORF from the six-frame translation, the canonical gene was picked in preference. For Figure 6A-F, to identify host proteins co-regulated by EBV and HCMV, raw files comprising EBV P3HR1 experiments WCL1-3 and PM1 were searched in combination with HCMV files from experiments WCL1-2 and PM1-2 (Weekes et al., 2014). For Figure 6G, raw files comprising EBV experiment PM1 were re-searched in combination with KHSV K5 files from the experiment described in this paper (Timms et al., 2013), against the human Uniprot database (February 4th, 2014).

Proteins were quantified by summing TMT reporter ion counts across all matching peptide-spectral matches using in-house software, as described previously (Pease et al., 2013). Briefly, a 0.003 Th window around the theoretical m/z of each reporter ion (126, 127N, 127C, 128N, 128C, 129N, 129C, 130N, 130C, 131) was scanned for ions, and the maximum intensity nearest to the theoretical m/z was used. We required every individual peptide used for quantitation to contribute sufficient TMT reporter ions (minimum of 1,250 per spectrum) so that each on its own provided a representative picture of relative protein abundance (McAlister et al., 2012). We additionally employed an isolation specificity filter to minimize peptide coisolation (Ting et al., 2011). Peptide-spectral matches with poor quality MS3 spectra (more than 9 TMT channels missing and/or a combined signal:noise ratio of less than 250 across all TMT reporter ions) or no MS3 spectra at all were excluded from quantitation. Protein quantitation values were exported for further analysis in Excel. Reverse and contaminant proteins were removed, then two

normalisation steps were employed. (A) each reporter ion channel was summed across all quantified proteins and normalized assuming equal protein loading across all 10 samples. (B) for calculation of p-values for P3HR1 experiments WCL1-3, the resulting values needed first to be normalised *between* experiments to enable their direct comparison. For each of experiments WCL1-3, each reporter ion value for each protein was therefore normalised to the sum of the uninduced and 24h gp350+ samples for that protein. For the WCL2 series, the average of the two unstimulated controls was used in the calculation. Normalised values were imported into Perseus version 1.5.1.6 (Tyanova et al., 2016), and 2-tailed t-test values calculated. For calculation of p-values for the Akata WCL experiment, as biological duplicates were quantified in the same experiment, only normalisation (A) described above was needed. One-way ANOVA values were added using Perseus. For t-test and ANOVA p-values, correction for multiple hypothesis testing was further applied using the Benjamini-Hochberg method. As Perseus only indicates p-values that are significant after correction but does not display or export corrected values, the method was employed via a macro written in Excel by one of the authors. For Figure S1C, the matrix of Pearson correlations was generated using Perseus. For Figures 1D and 7B, r^2 values were calculated in Excel. Gene Ontology terms were downloaded from www.uniprot.org. Hierarchical centroid clustering based on uncentered Pearson correlation for Figure S1D, or Euclidian distance for Figure S5C was performed using Cluster 3.0 (Stanford University) and visualized using Java Treeview (http://jtreeview.sourceforge.net). Statistical analyses including k-means clustering were performed using XLStat (Addinsoft). The cellular concentrations of viral proteins in whole cell lysates (Figure 7A) were calculated using a 'proteomic ruler' approach implemented in Perseus plugin (http://www.coxdocs.org/doku.php?id=perseus:user:plugins:proteomicruler:estimatecopynumbers). Briefly, intensity values that had been normalised assuming equal protein loading across all 10 samples were imported into Perseus. For P3HR1 samples, intensities were further corrected to normalise between experiments WCL1-3. Protein sequence lengths and molecular weights were downloaded for human, P3HR1 and Akata from Uniprot and additionally imported. Averaging was performed using the 'same normalisation for all columns' option. Scaling was using the histone proteomic ruler, assuming a ploidy of 2.

Pathway Analysis was performed using the Database for Annotation, Visualization and Integrated Discovery (DAVID) (Huang da et al., 2009) version 6.8 with default settings. A given cluster was always searched against a background of all proteins quantified within the relevant experiment. To generate lists of proteins for DAVID enrichment analysis, for P3HR1 WCL data, proteins were included if they were quantified in all of experiments WCL1-3. All proteins quantified in the Akata WCL and P3HR1 PM experiment were included. Lists of down- or up-regulated proteins were generated by employing a minimum 2-fold change, and p-value as described in Figure 1. Fold enrichment and Benjamini-Hochberg corrected p-values were downloaded from the DAVID website.
For Figure 6B we used the STRING database to identify known protein interactions (http://string-db.org/). For prediction of EBV viral proteins present at the PM (Figure 7C), we used a strategy described in our recent publication (Weekes et al., 2014). We initially used human protein data to delineate a cutoff that predicted PM location. For or every GO-annotated human protein quantified in experiment PM1, we calculated the ratio of peptides (experiments PM1)/(average (experiments WCL1, WCL2, WCL3)). 87% of proteins that were GO-defined non-PM had a ratio of <0.35; 84% of human proteins scoring above 0.35 were annotated as PM proteins, demonstrating the predictive value of this metric (Figure 7C). Applying this filter, we defined 9 high-confidence viral PM proteins, which included the majority of viral proteins previously identified at the surface of cells induced to replicate EBV, and excluded all proteins unlikely to be present at the cell surface based on their known function.

Database generation

A P3HR1 Uniprot database was generated from published sequence data (www.uniprot.org, accession number LN827548) (Palser et al., 2015). An Akata Uniprot database was downloaded (http://www.uniprot.org/citations/23152513). A678 and B95.8 strain EBV proteins have high sequence homology to P3HR1, and were included in addition to the P3HR1 protein in regions in which the P3HR1 genome was incompletely sequenced (A678 in preference to B95.1 where both were available). Specific additional proteins included for A678 were (Uniprot or Trembl where indicated): EBNAL-LP (Q1HVI8), EBNA3 (Q69138), EBNA6 (Q69140), BLLF1 (P68343), EBNA1 (Q1HVF7), BPLF1 (Q1HVH9). Specific additional proteins included for B95.8 were: LF3 (P0C727), BHLF1 (P03181), EC-RF4 (P03235), BCRF2 (Trembl Q8AZK8), BSLF2 (Trembl Q9QCF1). A 6-frame translation of the full P3HR1 and Akata nucleotide sequences were additionally generated, requiring each ORF to contain a minimum of >= 7 amino acids. For each of the 6 reading frames, each potential polypeptide was sequentially numbered starting from 1.

In a small number of cases, data searches identified viral proteins with the same gene name, but belonging to more than one EBV strain. In each case, the relevant Uniprot or Trembl terms, strains and protein names have been indicated, separated by '/' in Table S1. Signal : noise values and peptide numbers were summed to generate a single entry for each EBV protein.

Data and software availability
Mass spectrometry data reported in this paper will be made available through the PRIDE Archive, a member of the ProteomeXchange (PX) consortium.

Gene Set Enrichment Analysis (GSEA) Analysis.
The difference in the proteomics data at 24 hour post induction compared to uninduced sample was used to generate a ranked list for GSEA Preranked anlaysis using the Molecular Signatures Database v5.2 (C7:immunologic signatures) (Subramanian et al., 2005). Gene sets with nominal p value < 0.05 and false discovery rate (FDR) <0.25 were defined as significantly enriched gene sets, which were selected for visualization. GSEA first calculates the enrichment score (ES), which reflects the degree to which a gene set is overrepresented at the top or bottom of a ranked list of genes. GSEA calculates the ES by walking down the ranked list of genes, increasing a running-sum statistic when a gene is in the gene set and decreasing it when it is not. The magnitude of the increment depends on the correlation of the gene with the phenotype. The ES is the maximum deviation from zero encountered in walking the list. A positive ES indicates gene set enrichment at the top of the ranked list; a negative ES indicates gene set enrichment at the bottom of the ranked list. The normalized enrichment score (NES) is the primary statistic for examining gene set enrichment results. By normalizing the ES, GSEA accounts for differences in gene set size and in correlations between gene sets and the expression dataset; therefore, the normalized enrichment scores (NES) can be used to compare analysis results across gene sets. GSEA determines NES as follows: NES is equal to the actual ES, divided by the mean of ESs against all permutations of the dataset.

Supplemental References:

Calderwood, M.A., Holthaus, A.M., and Johannsen, E. (2008). The Epstein-Barr virus LF2 protein inhibits viral replication. J Virol *82*, 8509-8519.

Elias, J.E., and Gygi, S.P. (2007). Target-decoy search strategy for increased confidence in large-scale protein identifications by mass spectrometry. Nat Methods *4*, 207-214.

Elias, J.E., and Gygi, S.P. (2010). Target-decoy search strategy for mass spectrometry-based proteomics. Methods Mol Biol *604*, 55-71.

Eng, J.K., McCormack, A.L., and Yates, J.R. (1994). An approach to correlate tandem mass spectral data of peptides with amino acid sequences in a protein database. J Am Soc Mass Spectrom *5*, 976-989.

Huang da, W., Sherman, B.T., and Lempicki, R.A. (2009). Systematic and integrative analysis of large gene lists using DAVID bioinformatics resources. Nat Protoc *4*, 44-57.

Huttlin, E.L., Jedrychowski, M.P., Elias, J.E., Goswami, T., Rad, R., Beausoleil, S.A., Villen, J., Haas, W., Sowa, M.E., and Gygi, S.P. (2010). A tissue-specific atlas of mouse protein phosphorylation and expression. Cell *143*, 1174-1189.

McAlister, G.C., Huttlin, E.L., Haas, W., Ting, L., Jedrychowski, M.P., Rogers, J.C., Kuhn, K., Pike, I., Grothe, R.A., Blethrow, J.D., *et al.* (2012). Increasing the multiplexing capacity of TMTs using reporter ion isotopologues with isobaric masses. Anal Chem *84*, 7469-7478.

Palser, A.L., Grayson, N.E., White, R.E., Corton, C., Correia, S., Ba Abdullah, M.M., Watson, S.J., Cotten, M., Arrand, J.R., Murray, P.G., *et al.* (2015). Genome diversity of Epstein-Barr virus from multiple tumor types and normal infection. J Virol *89*, 5222-5237.

Pease, B.N., Huttlin, E.L., Jedrychowski, M.P., Talevich, E., Harmon, J., Dillman, T., Kannan, N., Doerig, C., Chakrabarti, R., Gygi, S.P., *et al.* (2013). Global analysis of protein expression and phosphorylation of three stages of Plasmodium falciparum intraerythrocytic development. J Proteome Res *12*, 4028-4045.

Petersen, C.P., Bordeleau, M.E., Pelletier, J., and Sharp, P.A. (2006). Short RNAs repress translation after initiation in mammalian cells. Mol Cell *21*, 533-542.

Rabson, M., Heston, L., and Miller, G. (1983). Identification of a rare Epstein-Barr virus variant that enhances early antigen expression in Raji cells. Proc Natl Acad Sci U S A *80*, 2762-2766.

Sanjana, N.E., Shalem, O., and Zhang, F. (2014). Improved vectors and genome-wide libraries for CRISPR screening. Nat Methods *11*, 783-784.

Sowa, M.E., Bennett, E.J., Gygi, S.P., and Harper, J.W. (2009). Defining the human deubiquitinating enzyme interaction landscape. Cell *138,* 389-403.
Subramanian, A., Tamayo, P., Mootha, V.K., Mukherjee, S., Ebert, B.L., Gillette, M.A., Paulovich, A., Pomeroy, S.L., Golub, T.R., Lander, E.S., *et al.* (2005). Gene set enrichment analysis: a knowledge-based approach for interpreting genome-wide expression profiles. Proc Natl Acad Sci U S A *102*, 15545-15550.
Takada, K., and Ono, Y. (1989). Synchronous and sequential activation of latently infected Epstein-Barr virus genomes. J Virol *63*, 445-449.
Timms, R.T., Duncan, L.M., Tchasovnikarova, I.A., Antrobus, R., Smith, D.L., Dougan, G., Weekes, M.P., and Lehner, P.J. (2013). Haploid genetic screens identify an essential role for PLP2 in the downregulation of novel plasma membrane targets by viral E3 ubiquitin ligases. PLoS Pathog *9*, e1003772.
Ting, Y.S., Shaffer, S.A., Jones, J.W., Ng, W.V., Ernst, R.K., and Goodlett, D.R. (2011). Automated lipid A structure assignment from hierarchical tandem mass spectrometry data. J Am Soc Mass Spectrom *22*, 856-866.
Tyanova, S., Temu, T., Sinitcyn, P., Carlson, A., Hein, M.Y., Geiger, T., Mann, M., and Cox, J. (2016). The Perseus computational platform for comprehensive analysis of (prote)omics data. Nat Methods *13*, 731-740.
Weekes, M.P., Antrobus, R., Talbot, S., Hor, S., Simecek, N., Smith, D.L., Bloor, S., Randow, F., and Lehner, P.J. (2012). Proteomic plasma membrane profiling reveals an essential role for gp96 in the cell surface expression of LDLR family members, including the LDL receptor and LRP6. J Proteome Res *11*, 1475-1484.
Weekes, M.P., Tomasec, P., Huttlin, E.L., Fielding, C.A., Nusinow, D., Stanton, R.J., Wang, E.C., Aicheler, R., Murrell, I., Wilkinson, G.W., *et al.* (2014). Quantitative temporal viromics: an approach to investigate host-pathogen interaction. Cell *157*, 1460-1472.
