# Supplementary material for: A Temporal Proteomic Map of Epstein-Barr Virus Lytic Replication in B Cells
Source: Cell Rep. 2017 May 16;19(7):1479–93. doi: 10.1016/j.celrep.2017.04.062 (PMC5446956; doi:10.1016/j.celrep.2017.04.062)
Supplement: Document S2. Article plus Supplemental Information [file mmc6.pdf]

Cell Reports

Resource

A Temporal Proteomic Map of Epstein-Barr Virus Lytic Replication in B Cells

Graphical Abstract

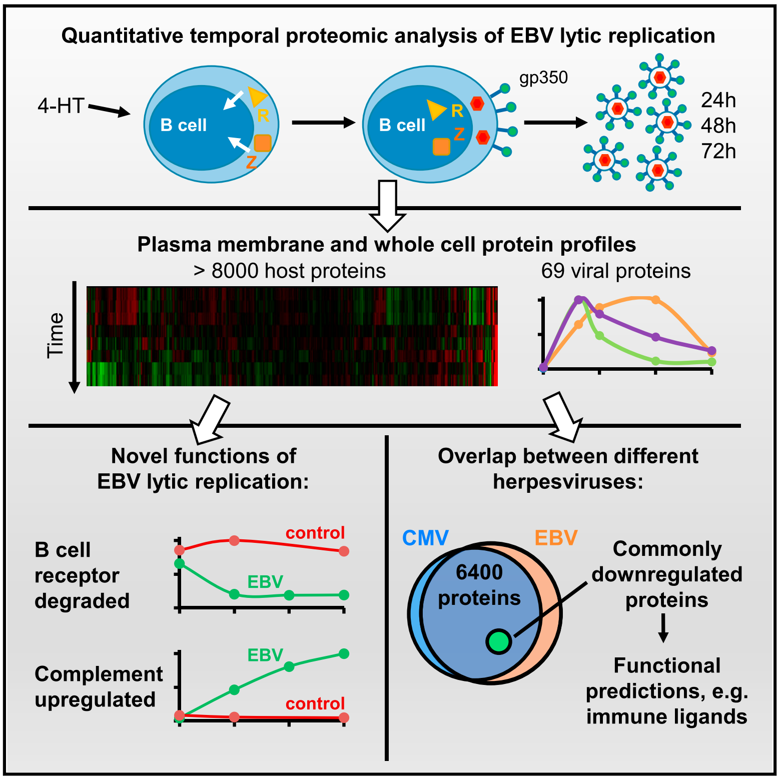

Authors

Ina Ersing, Luis Nobre,
Liang Wei Wang, ..., Steven P. Gygi,
Michael P. Weekes, Benjamin E. Gewurz

Correspondence

mpw1001@cam.ac.uk (M.P.W.),
bgewurz@partners.org (B.E.G.)

In Brief

Ersing et al. present a temporal proteomic map of EBV B cell lytic replication. Tandem-mass-tag-based proteomics uncover extensive remodeling of the human proteome by EBV, conserved across the two major EBV strains. Cell-cycle, innate, and adaptive immune pathways are modulated, complement is upregulated, and the B cell receptor is degraded by infection.

Highlights

- Unbiased global analysis of host and EBV proteome remodeling in lytic replication
- Temporal profiles of >8,000 host and 69 viral proteins, using type I and II EBV
- Both EBV types target the B cell receptor complex for degradation
- Conserved EBV and HCMV lytic cycle host targets are identified

Accession Numbers

PXD006317

Ersing et al., 2017, Cell Reports *19*, 1479–1493
May 16, 2017 © 2017 The Author(s).
http://dx.doi.org/10.1016/j.celrep.2017.04.062

OPEN ACCESS
CellPress

A Temporal Proteomic Map of Epstein-Barr Virus Lytic Replication in B Cells

Ina Ersing,[1,2,8] Luis Nobre,[3,8] Liang Wei Wang,[1,7] Lior Soday,[3] Yijie Ma,[1] Joao A. Paulo,[4] Yohei Narita,[1,6] Camille W. Ashbaugh,[1] Chang Jiang,[1] Nicholas E. Grayson,[5] Elliott Kieff,[1,6] Steven P. Gygi,[4] Michael P. Weekes,[3,9,*] and Benjamin E. Gewurz[1,7,9,10,*]
[1]Division of Infectious Disease, Department of Medicine, Brigham & Women's Hospital, Harvard Medical School, 181 Longwood Avenue, Boston, MA 02115, USA
[2]Institut für Klinische und Molekulare Virologie, Friedrich-Alexander-Universität Erlangen-Nürnberg, 91054 Erlangen, Germany
[3]Cambridge Institute for Medical Research, University of Cambridge, Hills Road, Cambridge CB2 0XY, UK
[4]Department of Cell Biology, Harvard Medical School, 240 Longwood Avenue, Boston, MA 02115, USA
[5]Wellcome Trust Sanger Institute, Hinxton, Cambridge, CB10 1HH, UK
[6]Department of Immunobiology and Microbiology, Harvard Medical School, 77 Avenue Louis Pasteur, Boston, MA 02115, USA
[7]Harvard Virology Program, Harvard Medical School, 77 Avenue Louis Pasteur, Boston, MA 02115, USA
[8]These authors contributed equally
[9]These authors contributed equally
[10]Lead Contact
*Correspondence: mpw1001@cam.ac.uk (M.P.W.), bgewurz@partners.org (B.E.G.)
http://dx.doi.org/10.1016/j.celrep.2017.04.062

SUMMARY

Epstein-Barr virus (EBV) replication contributes to multiple human diseases, including infectious mononucleosis, nasopharyngeal carcinoma, B cell lymphomas, and oral hairy leukoplakia. We performed systematic quantitative analyses of temporal changes in host and EBV proteins during lytic replication to gain insights into virus-host interactions, using conditional Burkitt lymphoma models of type I and II EBV infection. We quantified profiles of >8,000 cellular and 69 EBV proteins, including >500 plasma membrane proteins, providing temporal views of the lytic B cell proteome and EBV virome. Our approach revealed EBV-induced remodeling of cell cycle, innate and adaptive immune pathways, including upregulation of the complement cascade and proteasomal degradation of the B cell receptor complex, conserved between EBV types I and II. Cross-comparison with proteomic analyses of human cytomegalovirus infection and of a Kaposi-sarcoma-associated herpesvirus immunoevasin identified host factors targeted by multiple herpesviruses. Our results provide an important resource for studies of EBV replication.

INTRODUCTION

Epstein-Barr virus (EBV) is a gamma-herpesvirus that establishes persistent infection in >95% of adults worldwide. Two distinct strains of EBV have been identified, referred to as type I and II (Kieff and Rickinson, 2007). Following salivary transmission, EBV replicates in or translocates through epithelial cells and infects tonsillar B cells to establish lifelong B cell infection (Thorley-Lawson, 2015; Tugizov et al., 2013). Periodic viral reactivation re-infects the tonsillar epithelium, in which further rounds of lytic replication amplify the virus population that is secreted into saliva (Kenney and Mertz, 2014; Laichalk and Thorley-Lawson, 2005). EBV lytic reactivation is central to the virus life cycle and to most EBV-related diseases.

EBV is the etiologic agent of infectious mononucleosis and is closely linked to the pathogenesis of multiple human malignancies, with 200,000 EBV-associated cancers reported annually (Cohen et al., 2011). Lytic viral replication is implicated in the pathogenesis of nasopharyngeal carcinoma and oral hairy leukoplakia (Chien et al., 2001; Tsai et al., 2013) and may contribute to growth of B cell tumors, particularly in immunodeficiency (Arvey et al., 2012; Ma et al., 2011). The incidences of EBV-related Hodgkin lymphoma continue to rise in individuals with HIV infection despite antiretroviral therapy (Powles et al., 2009).

Upon lytic reactivation, EBV genes are sequentially expressed in immediate-early (IE), early (E,) and late (L) phases. The immediate early transcription factors ZTA (encoded by BZLF1) and RTA (encoded by BRLF1) jointly trigger the EBV lytic cycle. EBV early genes are synergistically induced by ZTA and RTA and encode the viral polymerase and replication machinery. Late viral genes encode structural proteins that encapsidate and mediate release of infectious virions (McKenzie and El-Guindy, 2015).

mRNA expression profiling has provided important information on the kinetics of viral gene expression upon lytic cycle induction in Burkitt lymphoma cell lines (Koganti et al., 2015; Yuan et al., 2006). Likewise, RNA sequencing (RNA-seq) of lymphoblastoid cell lines with varying degrees of lytic replication provided insights into B cell and virus transcription patterns induced by EBV reactivation (Arvey et al., 2012). However,

This is an open access article under the CC BY license (http://creativecommons.org/licenses/by/4.0/).

post-transcriptional effects may substantially alter the host and EBV proteome, and little is presently known about cell surface remodeling during EBV lytic replication. The comparative effects of type I and II EBV on human proteins are unknown.

We used tandem-mass-tag (TMT)-based MS3 mass spectrometry to perform quantitative temporal proteomic analysis of EBV replication in human Burkitt lymphoma B cells latently infected by type II EBV, prior to and at four time points after induction of lytic replication (Weekes et al., 2014). Selective plasma membrane (PM) protein enrichment enabled quantitation of global cell surface changes, without the need for specific antibodies. We quantified 8,318 host proteins, including 550 PM proteins and 69 EBV proteins, providing an in-depth temporal view of the host and viral proteome during B cell replication. Our analysis identified key host targets of EBV lytic replication, including multiple immune pathways. Unexpectedly, an EBV early factor targets the B cell receptor (BCR) complex for proteasomal degradation. We found that host protein abundance was similarly remodeled by type I EBV lytic replication in Burkitt lymphoma cells, identifying evolutionarily conserved EBV B cell targets. We further highlight host proteins co-targeted by multiple human herpesviruses and provide an in-depth resource for studies of EBV lytic replication.

RESULTS

Quantitative Temporal Viromic Analysis of EBV Replication

We used the well-characterized type II EBV$^+$ Burkitt lymphoma cell line P3HR1 to quantify changes in PM and whole cell lysate (WCL) protein expression by 10-plex TMT and MS3 mass spectrometry at three reference time points after induction of lytic EBV replication (experiments WCL1 and PM1, Figure 1A). Although it is difficult to induce viral replication in most latently infected B cell lines, P3HR1 is highly permissive for induced EBV lytic reactivation and exhibits low level spontaneous lytic replication (Balachandran et al., 1986; Verma et al., 2009). To ensure sufficient cells entered the EBV lytic replication cycle for proteomic analysis, the P3HR1 line was engineered to express the EBV immediate early proteins ZTA and RTA fused to a 4-hydroxy tamoxifen (4-HT)-dependent mutant estrogen receptor binding domain (ZHT and RHT, respectively) (Calderwood et al., 2008; Chiu et al., 2013). The addition of 4-HT causes ZHT/RHT nuclear translocation, triggering EBV lytic replication. As is commonly observed, abortive lytic replication occurred in a fraction of P3HR1 cells, in which ZTA/RTA do not trigger the full lytic program and infectious particles are not produced (Chiu and Sugden, 2016; Klein et al., 1974; Lin and Raab-Traub, 1987; Miller et al., 1974). We therefore used flow cytometry to sort 4-HT-induced P3HR1-ZHT/RHT cells into fully lytic and abortive lytic populations, based on expression of the EBV late cycle protein gp350, routinely achieving >40% of cells with PM gp350 expression (Figure 1A). We performed two additional replicate proteomic experiments at 24, 48, and 72 hr after induction of replication, which also enabled assessment of an early time point of infection (15 hr, experiment WCL2), or a full time course of analysis of mock 4-HT-induced parental control cell line, which lacks the ZHT/RHT system (experiment WCL3) (Figure S1A).

We quantified 8,249 human proteins across all three P3HR1 replicates (WCL1–3), including 6,307 proteins in all three replicates. We additionally quantified 69 EBV proteins (63 in all replicates). In experiment PM1, 550 PM proteins were quantified (Figures 1B, 1C, and S1B). We observed a high degree of correlation between biological replicates (Figure S1C).

To validate our results in a second Burkitt lymphoma cell system and to determine the extent to which proteome remodeling is conserved between type I and II EBV replication, we next analyzed Akata cells (Figure 1A). Type I EBV$^+$ Akata cells are highly permissive for EBV lytic replication in response to anti-immunoglobulin G (IgG) cross-linking (Takada and Ono, 1989). We examined biological duplicates at 0 and 48 hr after Ig cross-linking and again used flow cytometry to sort induced cells into fully lytic and abortive lytic populations, based on EBV gp350 PM expression at 48 hr. To control for additional effects of anti-IgG stimulation, we performed parallel analyses of unstimulated and mock anti-IgG-induced EBV-negative Akata control cells (Figure 1A). These analyses quantified 7,041 proteins. Biological replicates clustered tightly (Figure S1D), and a comparison of changes in gp350$^+$ Akata and P3HR1 cells revealed a high degree of correlation, with an r^2 value of 0.70 (Figure 1D). All data are shown in Table S1, where the worksheet "Plotter" is interactive, enabling generation of temporal graphs of PM and WCL expression of any of the human and viral proteins quantified. Throughout this manuscript, all analyses are based on the average values from all analyzed replicates.

Quantitative Temporal Viromics Identifies Down- or Upregulated Pathways

We used DAVID software (Huang et al., 2009) to identify pathways enriched among proteins significantly down- or upregulated during lytic EBV replication in WCL and PM samples from gp350$^+$ P3HR1 or Akata cells. The term "antigen binding" or "B cell receptor signaling pathway" was enriched 14.7- to 22.3-fold among downregulated proteins compared to background (Figures 2A and S2A). Unexpectedly, the most highly downmodulated host factors included multiple BCR components: immunoglobulin M heavy chain (IgM) in P3HR1 and IgG in Akata, signaling chains CD79A and CD79B (also called Igα and Igβ), and the immunoglobulin joining chain (IgJ), which regulates multimerization of secretory IgM and IgA and is necessary for their poly-Ig receptor-mediated transfer across mucosal epithelium (Figures 1B, 1C, and 2B). Ig heavy-chain class switching was unlikely to account for the observed result, as we did not observe an accumulation of a different Ig heavy-chain isotype in either P3HR1 or Akata. Among significantly upregulated proteins, DAVID analysis identified the complement and blood coagulation pathways as highly significantly induced (Figures 2A, 2C, and S2A).

We additionally validated our proteomic findings by immunoblot for key innate immune signaling components AIM2, a cytosolic receptor for double stranded DNA with roles in sensing cytomegalovirus replication (Rathinam et al., 2010) and the transcription factors IRF1, IRF4, and STAT2, which each have important roles in antiviral responses (Figure S2B) (Blaszczyk et al., 2016; Taniguchi et al., 2001).

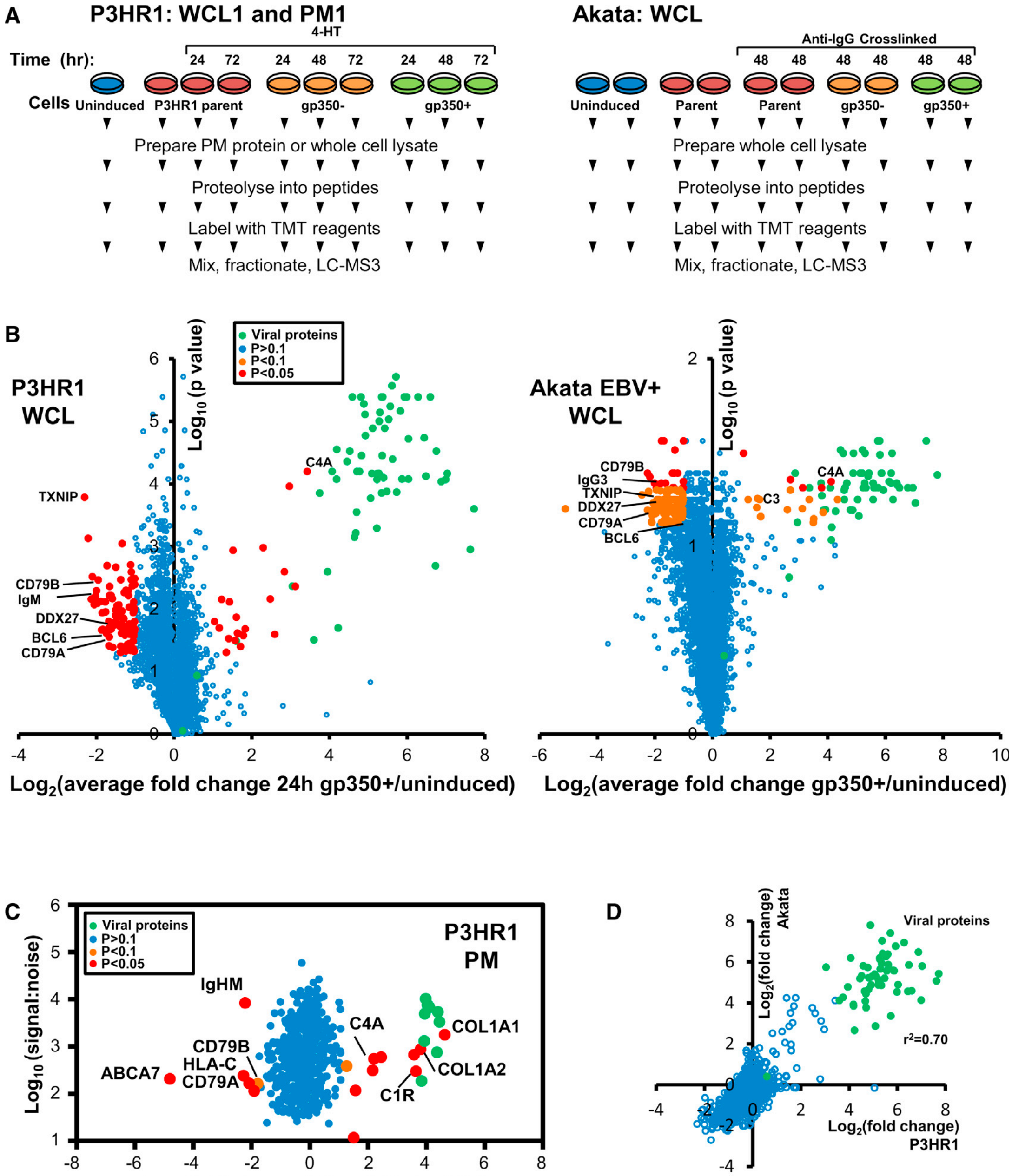

Figure 1. Temporal Proteomic Profiling of EBV B Cell Lytic Replication

(A) Workflow of experiments WCL1 and PM1 in P3HR1 cells and a WCL experiment in Akata cells. Parental P3HR1 cells were analyzed at 0, 24, and 72 hr after 4-HT induction. P3HR1-ZHT/RHT cells were analyzed at 0, 24, 48, and 72 hr after 4-HT induction. Parental EBV-negative or EBV-positive Akata cells were analyzed at 0 and 48 hr after induction. Cells were sorted by FACS in order to separate lytic gp350^{+} cells from abortive lytic gp350^{-} cells.

(B) Volcano plot of proteins quantified in all three P3HR1 WCL experiments (left panel) or the Akata WCL experiment (right panel). p values were estimated using a two-tailed t test (left panel) or a one-way ANOVA (right panel). In each case, values were corrected for multiple hypothesis testing using the method of Benjamini and Hochberg. Red dots: >2-fold change and $p < 0.05$. Orange dots: >2-fold change and $p < 0.075$. Green dots: viral proteins.

(C) Scatterplot of PM proteins quantified in experiment PM1. Significance A was used to estimate p values (Cox and Mann, 2008), which were corrected using the method of Benjamini and Hochberg.

(D) Correlation between average fold change at 24 hr (gp350^{+}/uninduced) in P3HR1 cells (replicates WCL1–3) and average fold change (gp350^{+}/uninduced) in Akata cell duplicates, for proteins quantified in all experiments.

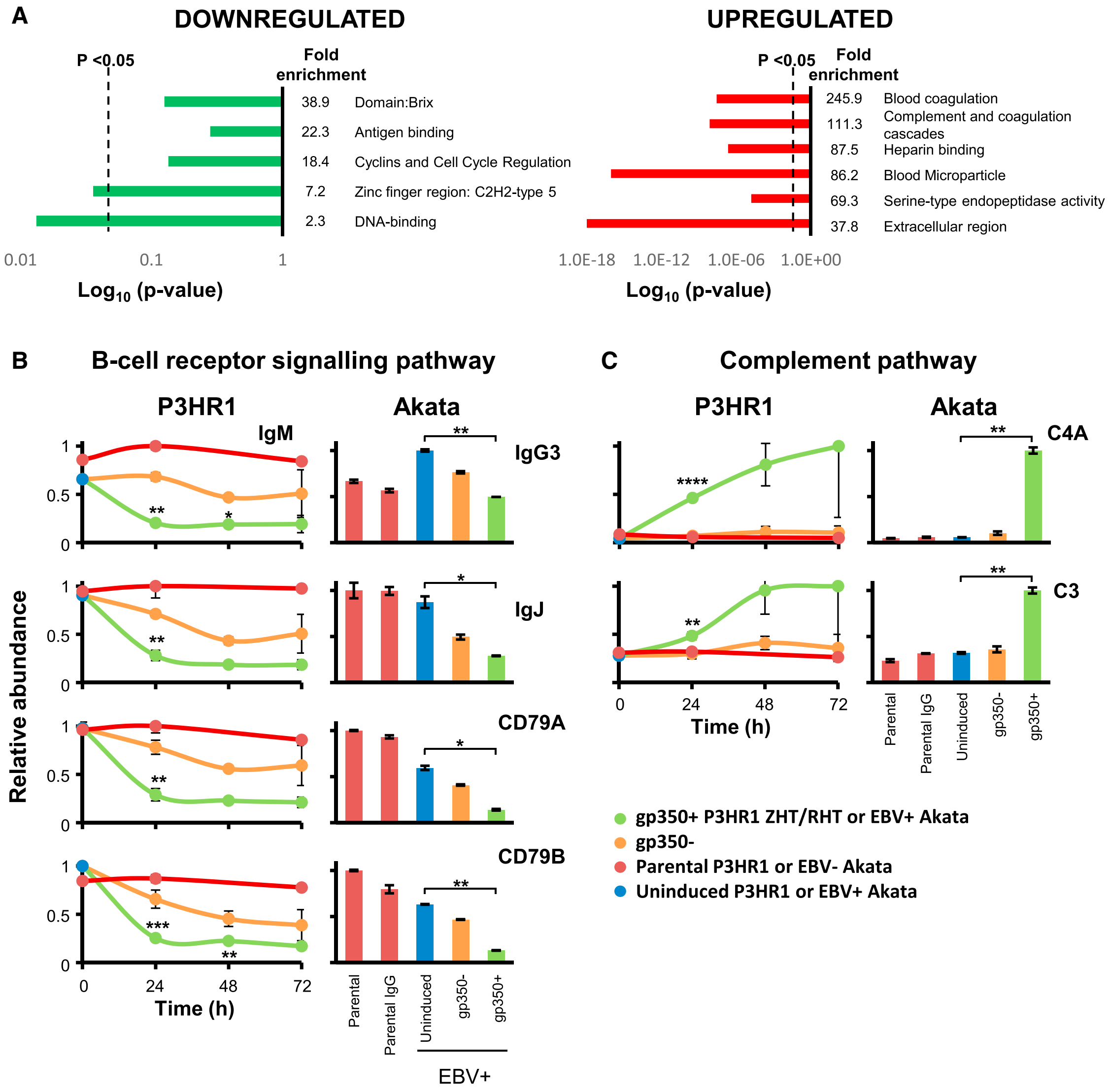

Figure 2. Enrichment Analysis Identifies EBV-Induced Downregulation of the BCR Complex and Upregulation of the Complement Pathway

(A) Functional enrichment within all proteins that were significantly ($p < 0.05$) downregulated an average of >2-fold by lytic EBV replication in P3HR1 replicates WCL1–3 (red dots in Figure 1B, left panel). A background of all quantified proteins was used. A similar analysis was performed for proteins significantly upregulated >2-fold. Dotted lines: $p = 0.05$. Components of each cluster are shown in Tables S2A and S2B.

(B) All BCR components quantified. Temporal plots (left-hand panels) illustrate data averaged over P3HR1 experiments WCL1–3. The parental cell line was measured only in experiments WCL1 and WCL3. Right-hand panels show corresponding results from Akata cells. Error bars ±SEM (triplicates) or range (duplicate). p values were calculated as described in Figure 1B: ****$p < 0.0005$, ***$p < 0.005$, **$p < 0.05$, *$p < 0.075$.

(C) Upregulated complement components, illustrated as described in (B).

An Early-Expressed EBV Protein Targets the BCR Complex for Degradation

IgM, CD79A, CD79B, and J chain were all downregulated by 15 hr of EBV lytic induction in gp350$^+$ P3HR1 cells (Table S1). We validated changes in IgM and CD79A by immunoblot. Moderate IgM and CD79A loss were observed in gp350$^-$ cells by the 72-hr time point (Figure 3A), which may reflect downregulation by an early-expressed EBV protein during abortive replication in these cells. We additionally validated changes in IgM by flow cytometry, both by 4-HT induction in P3HR1-ZHT/RHT cells and chemical induction in parental P3HR1 cells (Figures 3B and 3C).

EBV lytic reactivation typically occurs in B cells expressing either IgM or class-switched IgG heavy chains. Induction of EBV reactivation in IgG$^+$ Akata cells by anti-Ig cross-linking or by treatment with sodium butyrate led to robust downregulation of IgG and CD79A/B (Figures 2 and 3D). Acyclovir inhibits the viral polymerase, preventing EBV late gene expression (Colby et al., 1980; Feighny et al., 1981). Acyclovir treatment of 4-HT-induced P3HR1 cells did not appreciably affect IgM

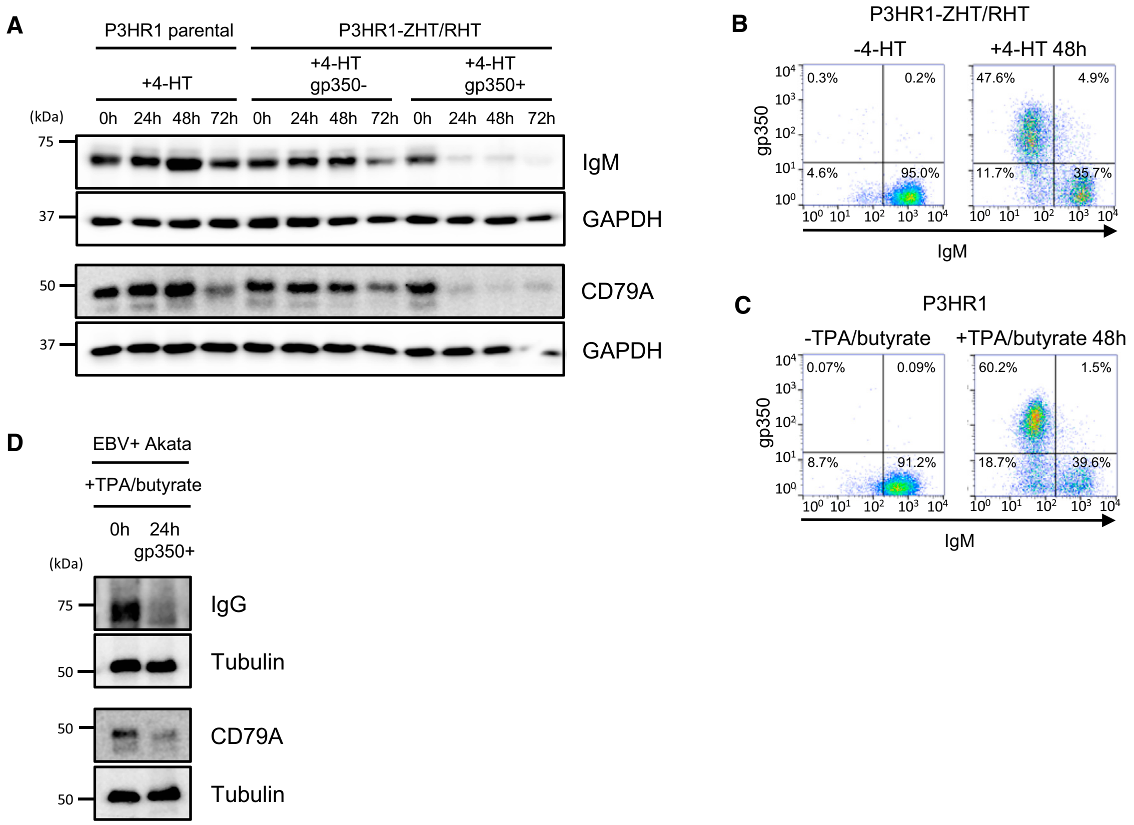

Figure 3. EBV Lytic Replication Targets the BCR Complex for Degradation

(A) Immunoblot of whole-cell extracts indicate downregulation of IgM and CD79A in gp350$^+$ ZHT/RHT cells, representative of three independent experiments.

(B and C) Downregulation of IgM during P3HR1 lytic EBV replication confirmed by flow cytometry. Shown are representative plots for three independent experiments of uninduced (B; left) and 48-hr 4-HT-induced P3HR1 (B; right) or of uninduced (C; left) and 48 hr 12-O-Tetradecanoylphorbol-13-Acetate (TPA)/butyrate-induced P3HR1 (C; right).

(D) EBV lytic replication destabilizes the IgG BCR complex. EBV$^+$ Akata B cells were induced to undergo lytic replication by TPA/butyrate treatment for 24 hr. WCL from sorted gp350$^+$ cells were immunoblotted are as indicated.

downregulation, even though it prevented expression of the late antigen gp350 (Figure 4A). These results are consistent with a model in which an early-expressed EBV gene, conserved between type I EBV (Akata) and type II EBV (P3HR1) strains, targets the BCR for degradation independently of heavy-chain isotype.

Lysosomal blockade had little effect on lytic replication-induced loss of IgM; however, inhibition of the proteasome by bortezomib stabilized IgM in gp350$^+$ cells (Figures 4B–4D). By immunofluorescence, we similarly observed IgM loss in 4-HT-induced P3HR1-ZHT/RHT cells that was rescued by bortezomib, without appreciable effect on early antigen expression (Figure 4E).

IgM distribution appeared distinct in control versus bortezomib-treated 4-HT-induced cells, suggesting accumulation in an intracellular pool, for example a cytoplasmic aggresome (Figure 4E). To further test this possibility, we examined immunopurified P3HR1-ZHT/RHT IgM complexes from uninduced and induced cells. EBV lytic replication in the presence of bortezomib increased levels of poly-ubiquitin conjugates attached to immune-purified IgM complexes (Figure 4F). Taken together, our results suggest that an EBV early gene targets cell-surface and intracellular BCR pools for ubiquitin-mediated proteasomal degradation.

EBV Lytic Replication Induces B Cell Complement Pathway Expression

Complement proteins C3, C4A, and C7 were substantially upregulated by EBV replication in P3HR1 and Akata cells, in addition to C5 and C9 in P3HR1 (Figure 2C; Table S1). Complement component expression was detectable by 24 hr after induction and peaked at 48–72 hr. Similarly, C1R, and C4A were upregulated at the PM of gp350$^+$ but not gp350$^-$ P3HR1 cells (Figure 1C). To our knowledge, complement expression has not previously been observed in B cells.

We excluded the possibility of an artifact due to deposition of serum complement proteins from cell culture media via qRT-PCR for C3 and C4A in gp350$^+$ cells (Figure S2C). The classical pathway activator mannose activated serine protease 1 (MASP1) was similarly increased by EBV lytic replication (Figure S2C). Furthermore, polymorphisms in C3, C4A, and C5 protein sequences revealed that multiple identified peptides were of human and not of bovine origin (Table S3). These data indicate

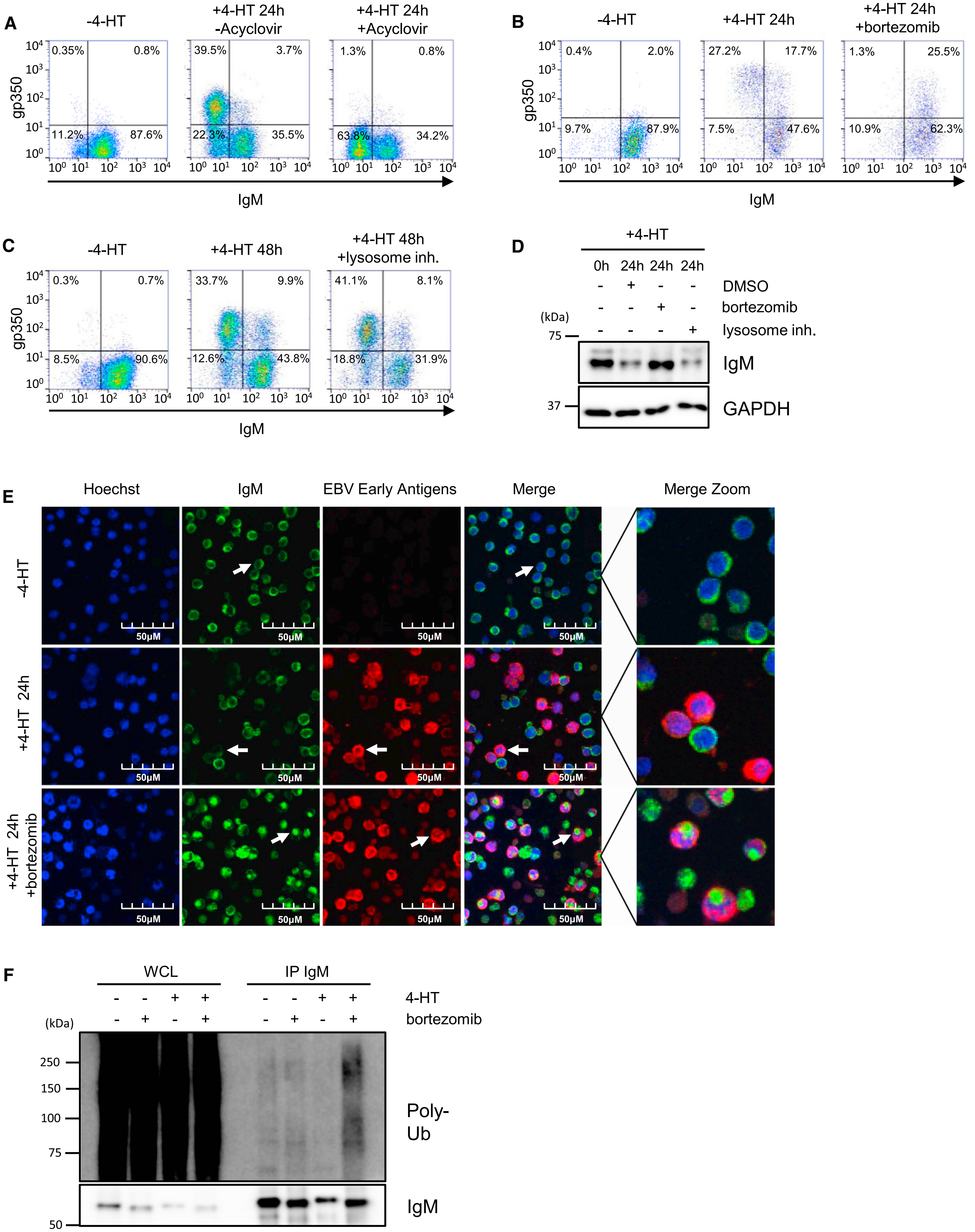

(legend on next page)

that EBV replication induces mRNA and protein expression of multiple complement components, and that complement components are deposited on lytic B cell PM. Complement induction may be a host response to EBV infection or may instead play an undefined role in EBV replication or spread, for example, through recently appreciated intracellular roles in host metabolism (Hess and Kemper, 2016). By contrast, lytic replication of the related herpesvirus human cytomegalovirus (HCMV) did not induce complement production in human fibroblasts (Weekes et al., 2014).

EBV Lytic Program Effects on Host Cell-Cycle Regulators

The EBV lytic cycle promotes a pseudo-late G1/S-phase to support EBV replication, through partially understood mechanisms (Chiu et al., 2013; Hammerschmidt and Sugden, 2013; Kenney, 2007; Paladino et al., 2014). Enrichment analysis suggested that multiple cyclin-dependent kinases (CDK) or CDK regulators were downregulated during lytic replication (Figure 2A; Table S2A), including the cyclin-dependent kinase CDK4, which phosphorylates retinoblastoma family proteins to promote G1/S progression. A comprehensive search of our P3HR1 and Akata data revealed additional downmodulated cell-cycle regulators including tyrosine kinase WEE1 (Figure 5A), which inhibits entry into mitosis (Matheson et al., 2016); the licensing factor CDT1, which is a key component of the pre-replication complex and p15, which binds to CDK4 and inhibits G1/S progression (Havens and Walter, 2011). The observed effects on each of these key cell-cycle regulatory factors may contribute to establishment of the pseudo-late G1/S phase environment.

mTOR promotes cell-cycle progression through S6K and eIF4E pathways, though specific roles in EBV lytic replication remain incompletely characterized (Adamson et al., 2014). Interestingly, we observed the S6K target programmed cell death 4 (PDCD4) to be among the most highly downregulated protein by EBV replication in P3HR1 cells; a similar effect was observed in Akatas (Figures 5B and 5C). Since S6K-mediated phosphorylation triggers PDCD4 ubiquitination by the cullin ubiquitin ligase β-TRCP (Dorrello et al., 2006), the observed PDCD4 loss suggests that EBV lytic replication may activate the S6K pathway. We confirmed that PDCD4 was stabilized in 4-HT-induced P3HR1 cells by small molecule antagonists of proteasome or cullin ubiquitin ligase activity (Figure 5D). Since PDCD4 suppresses cap-dependent translation (Yang et al., 2003), we used cap-dependent firefly and cap-independent renilla luciferase reporters, revealing that EBV lytic induction significantly increased cap-dependent translation (Figure 5E).

EBV-encoded LMP2A is a dedicated activator of mTOR in latent EBV infection (Cen and Longnecker, 2015; Moody et al., 2005) that is also expressed in the viral lytic phase (Yuan et al., 2006), suggesting it may trigger PDCD4 loss. Interestingly, we found that despite the absence of EBNA2 in P3HR1, LMP1, and LMP2A expression were nonetheless robustly induced by 4-HT (Figure 5F). While EBNA2 drives LMP1 expression from the ED-L1 promoter in EBV latency III, EBV lytic replication may instead use the LT-R1 promoter located in the terminal repeats, which mediates LMP1/LMP2A expression in an EBNA2-independent manner (Kieff and Rickinson, 2007). Immunoblot analysis demonstrated that PDCD4 expression was markedly reduced by stable LMP2A expression in P3HR1 cells (Figure 5G). Likewise, inhibition of the LMP2A/mTOR pathway kinases SYK by fostamatinib or PI3K by idelalasib, or blockade of mTOR itself by rapamycin, each stabilized PDCD4 in 4-HT-induced P3HR1 cells (Figure 5H). Taken together, these results indicate that LMP2A supports EBV lytic replication by de-repressing PDCD4 effects on cap-dependent translation. PDCD4 downregulation may be of wider importance to other herpesviruses, as we previously found that HCMV also downregulates this protein (Weekes et al., 2014).

Effects on B Cell Transcription Factor Networks

The physiological trigger for reactivation of latent EBV in B cells remains elusive; however, EBV replication is thought to initiate upon plasma cell differentiation (Laichalk and Thorley-Lawson, 2005). Interestingly, gene set enrichment analysis (GSEA) (Subramanian et al., 2005) identified enrichment of the plasma cell network in gp350^{+} P3HR1 cells (Figures S3A and S3B). Furthermore, DAVID analysis (Huang et al., 2009) suggested that EBV lytic replication downmodulated multiple B cell transcription factors (TFs) which are known to be suppressed upon germinal center B cell differentiation into plasma cells (Figure S4A; Table S3A). For instance, BCL6, which is critical for the maintenance of germinal center B cell fate, was decreased by ~85% at 24 hr after induction of replication in P3HR1 and also significantly decreased in gp350^{+} Akata cells (Figure S4A). CRISPR knockout of the ubiquitin ligase FBXO11, which controls BCL6 steady-state levels in uninfected B cells (Duan et al., 2012), did not inhibit EBV-mediated BCL6 loss (Figure S4B). By contrast, BCL6 mRNA levels were significantly decreased by 24 hr after lytic induction by 4-HT (Figure S4C). These results suggest that EBV lytic replication blocks BCL6 expression prior to translation. Similarly, Myc is repressed during plasma cell terminal differentiation, and MYC levels were reduced by nearly 80% in gp350^{+} P3HR1 and Akata cells by 24 hr. ETS1 and ID3 each inhibit plasma cell differentiation (Nutt et al., 2015) and were

Figure 4. An EBV Early Factor Targets IgM for Proteasomal Degradation

(A–C) Flow cytometry of P3HR1-ZHT/RHT cells, treated with acyclovir (A), bortezomib (B), or lysosome inhibitors (C), as indicated. Shown are representative plots for three independent experiments.

(D) IgM or GAPDH immunoblot analysis of WCL from P3HR1-ZHT/RHT treated with bortezomib or lysosome inhibitors, representative of three independent experiments.

(E) Immunofluorescence analysis of Hoechst stain, IgM, or EBV early antigen expression in P3HR1 ZHT/RHT cells treated with 4-HT and bortezomib, as indicated. Proteasome inhibition results in intracellular accumulation of IgM in early antigen^{+} cells.

(F) Immunoblot analysis of poly-ubiquitin chains (poly-Ub) from WCL or IgM complexes immunopurified from P3HR1 ZHT/RHT cells, induced for EBV lytic replication for 24 hr, in the absence or presence of bortezomib, as indicated. Blots are representative of three independent experiments.

OPEN ACCESS
CellPress

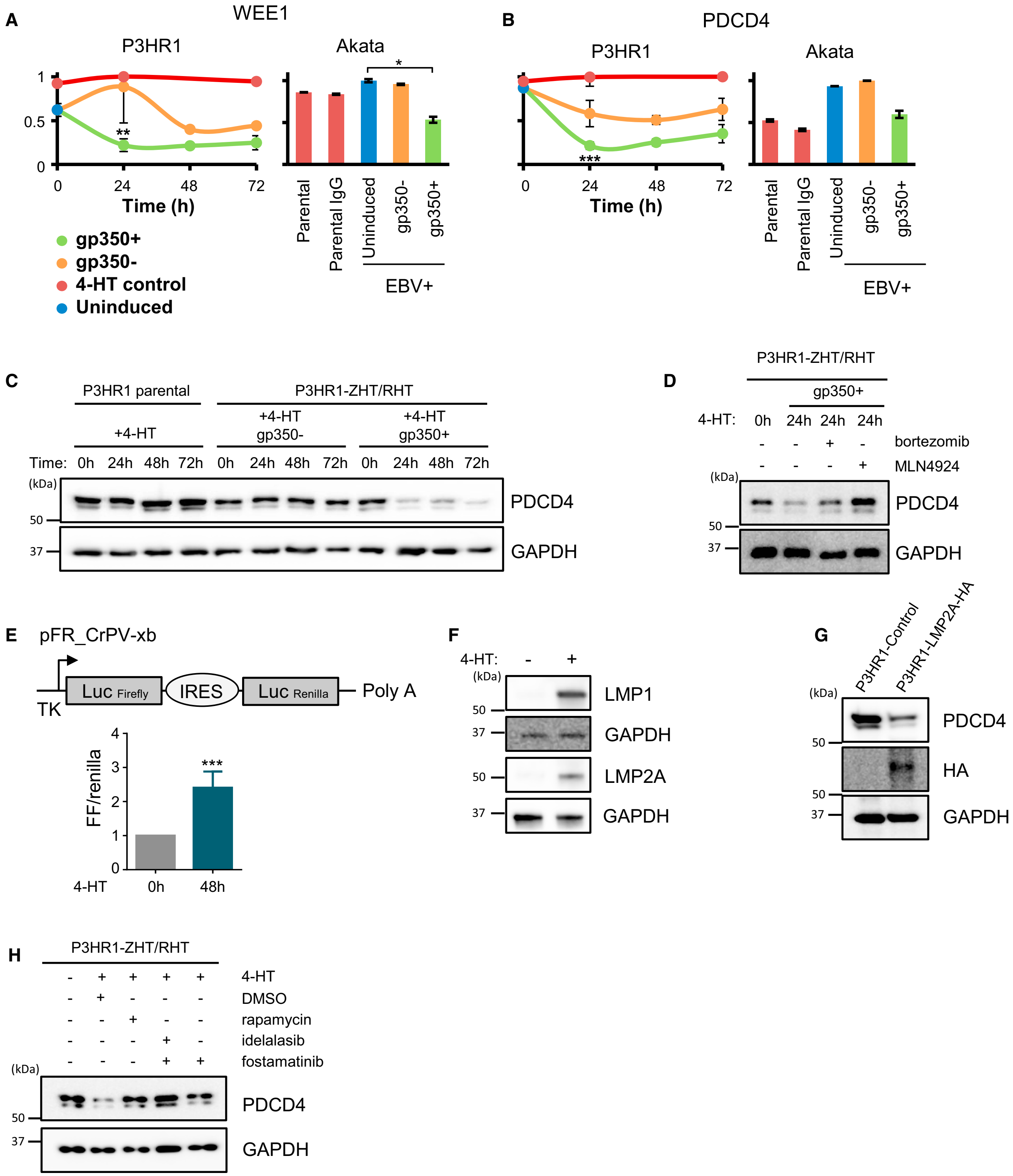

Figure 5. EBV Lytic Replication Suppresses Cell-Cycle Regulators WEE1 and PDCD4 and Increases Cap-Dependent Translation

(A and B) Temporal proteomic analysis of WEE1 (A) and PDCD4 (B). ***p < 0.01, **p < 0.05, *p = 0.1.

(C and D) Immunoblots demonstrate that EBV lytic replication triggers PDCD4 protein loss (C), which is restored by proteasome antagonist bortezomib or neddylation antagonist MLN4924, representative of three independent experiments (D).

(legend continued on next page)

similarly downregulated in gp350$^+$ P3HR1 and Akata cells (Figure S4A). Notably, the deubiquitinase USP1 stabilizes ID3 (Williams et al., 2011), and USP1 was also suppressed by EBV lytic replication (Table S1). A transcription module composed of IRF8 and SPI1/PU.1 limits plasma cell differentiation (Nutt et al., 2015), and each was downmodulated in gp350$^+$ P3HR1 and Akata cells by 24 hr (Figure S4A; Table S1).

We observed significant upregulation in gp350$^+$ cells of TFs that have key roles in plasma cell development or terminal B cell differentiation including IRF4, the IRF4 target ZBTB20, Fos, and FosB (Table S1; Figure S4B) (Nutt et al., 2015). While plasma cell differentiation stimulates EBV lytic replication, our data suggest that EBV lytic replication in germinal-center-derived Burkitt lymphoma B cells may, in turn, remodel the B cell TF network to mimic key aspects of plasma cell differentiation.

Identification of PM and Whole-Cell Proteins Commonly Targeted by Diverse Herpesviruses

Certain key proteins important in innate antiviral immunity are known to be targeted by more than one, or sometimes multiple viruses (Schoggins et al., 2011; Schreiner and Wodrich, 2013). To identify proteins jointly targeted by EBV and other human herpesviruses, we combined our data with our previous analyses of either temporal HCMV infection or of cell-surface proteins targeted by the Kaposi's-sarcoma-associated herpesvirus (KSHV) viral ubiquitin E3 ligase K5 (Timms et al., 2013; Weekes et al., 2014). We applied stringent filters to identify proteins that were (1) regulated >2-fold in all experiments by both viruses; (2) regulated by EBV as opposed to the 4-HT added to induce lytic-cycle replication (Figure 6). Of 6,389 whole-cell proteins quantified in both HCMV and EBV analyses, ten downregulated host factors met all criteria, of which eight function in poly(A) RNA binding and six are known to interact (Figures 6A–6C; Table S4).

A similar analysis of PM proteins downregulated by both EBV and HCMV identified 12 proteins, enriched in molecules functioning in synapse organization (Figures 6D–6F; Table S4). We previously identified HCMV-induced downregulation of multiple members of the protocadherin family and provided initial confirmatory evidence that members of this family are activating natural killer (NK) ligands (Weekes et al., 2014). Protocadherin γC3 was also downregulated during EBV infection (Figure 6E, Table S4), in addition to three Neuroligins (NLGN1, 3, and 4X) and Bone Morphogenetic Protein Receptor Type 1A (BMPR1A). NLGN1, NLGN4X, and BMPR1A were also targeted by KSHV K5 (Figure 6G; Table S4), suggesting that these molecules may be of particular importance in herpesviral pathogenesis. A recent report suggested the existence of an as-yet-unidentified DNAM-1 ligand of particular importance for killing early-lytically infected B cells, whereas late-lytic cells are highly resistant to killing (Williams et al., 2015). It is thus possible that one or some of the protocadherins and neuroligins fulfill this function.

By stringent criteria, 48 proteins were commonly upregulated by EBV and cytomegalovirus (CMV) (Figures 6D and S2; Table S4). DAVID software suggested that this group was particularly enriched in proteins with Interpro categories that included "Unfolded protein binding," "Endoplasmic reticulum (ER) lumen," "chaperone," suggesting increased ER stress. Further studies are required to determine whether these changes reflect directly targeted molecules versus non-specific effects of herpesviral infection.

Quantitative Analysis of the EBV Virome

Prior proteomic analyses of EBV lytic protein expression have identified 16 or 44 EBV proteins at single time points of viral reactivation (Koganti et al., 2015; Traylen et al., 2015). A particular benefit of our proteomic analysis is the opportunity not only to identify viral proteins without specific reagents, but additionally to quantify (1) their expression over time using TMT reporters and (2) the relative abundance of each protein.

We quantified 63 canonical EBV-encoded proteins in all three P3HR1 WCL samples. We used a "proteomic ruler" approach (Wiśniewski et al., 2014) to estimate cellular concentration (Figure 7A). We estimated that the most abundantly expressed EBV protein, DNA polymerase processivity factor BMRF1, was present at ~1,000 times greater concentration than EBNA-3C, the least expressed protein. The top three most abundant proteins, BMRF1, BLRF2, and BALF2 accounted for 25% of the overall EBV viral protein abundance, and, in total, tegument proteins accounted for 39% of the total EBV protein abundance (Figure 7A). The least expressed EBV proteins that we detected included three Epstein-Barr nuclear antigens, which have roles in transcription regulation. Notably, the P3HR1 EBV strain does not encode the related EBNA2 TF and expresses a truncated form of EBNA-LP (Palser et al., 2015). 57/63 of these proteins were also quantified in Akata cells and displayed a similar relative abundance (Figure 7B).

To determine whether non-canonical EBV-encoded polypeptides were expressed in gp350$^+$ cells, we searched our data against a six-frame translation of the P3HR1 genome (Palser et al., 2015). We identified only a single non-canonical EBV polypeptide, which was quantified by two peptides in experiment WCL1 and two peptides in WCL2. A further peptide from the same open reading frame (ORF) was identified in experiment WCL3; however, insufficient ions were present for quantitation (Figure S5A). DNA sequence encoding a similar ORF is present in the Akata genome (Grayson et al., 1985); however, peptides

(E) EBV lytic replication increases cap-dependent translation in P3HR1-ZHT/RHT cells. Cells were transiently transfected with the cap-dependent translation firefly (FF) and cap-independent renilla luciferase reporter vector pFR_CrPV-xb and rested for 24 hr. Cells were then left uninduced or were 4-HT-induced for 48 hr, and FF and renilla activity were quantified by dual luciferase assay. Shown are mean fold changes of renilla-normalized FF luciferase activity. Median and SEM values from three independent experiments. ***$p < 0.001$.

(F) LMP1 and LMP2A immunoblot of WCL from P3HR1 ZHT/RHT cells treated with 4-HT, as indicated.

(G) LMP2A downregulates PDCD4. Immunoblot of WCL from uninduced P3HR1 cells stably expressing control or HA-tagged LMP2A is representative of three independent experiments.

(H) Blockade of mTOR (rapamycin), PI3K (idelalasib), or SYK (fostamatinib) rescued cellular PDCD4 during EBV lytic replication. Immunoblot of sorted P3HR1 gp350$^+$ cell WCL is as indicated. Blots are representative of three independent experiments.

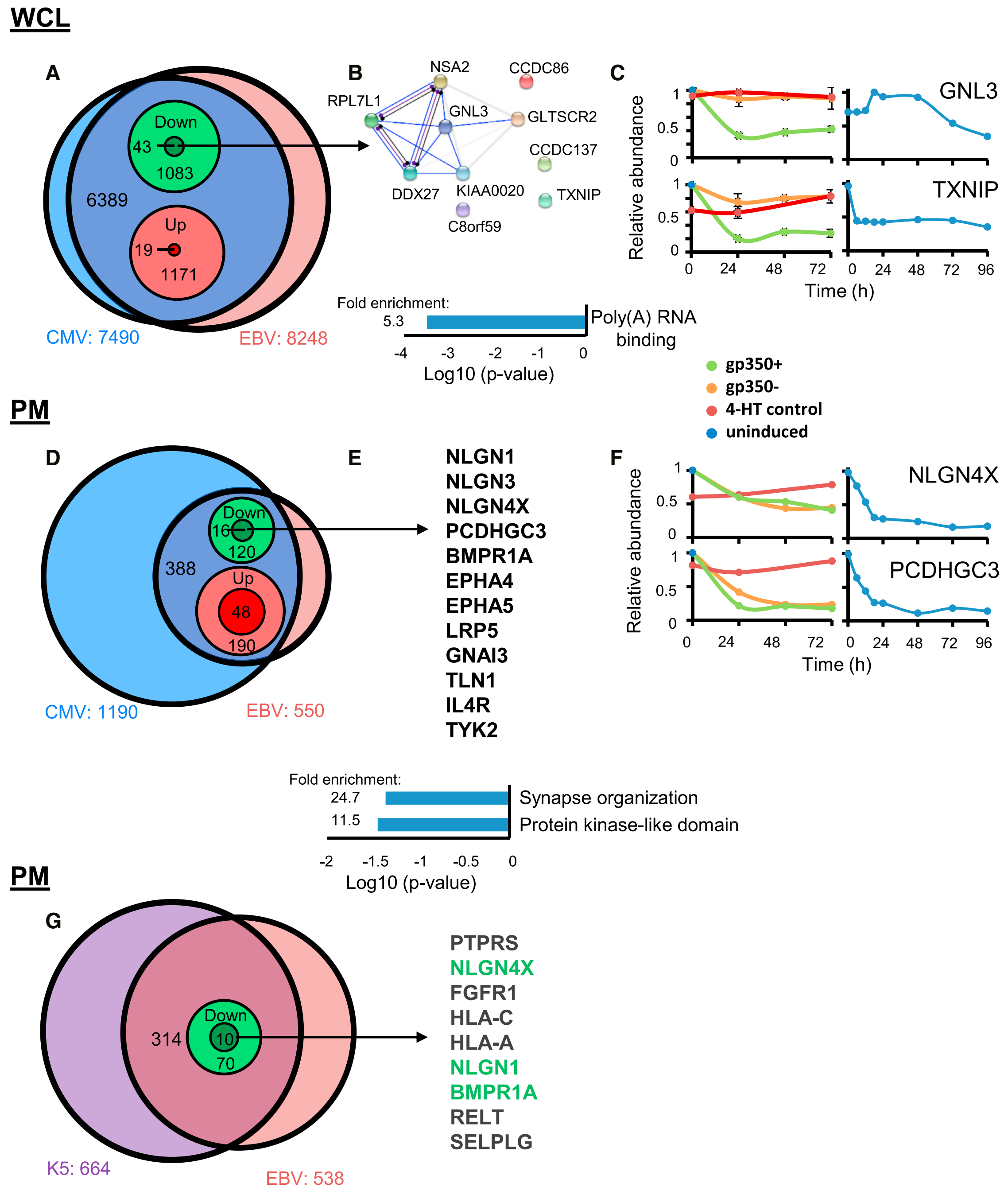

Figure 6. Whole-Cell and PM Proteins Co-regulated by EBV, CMV, and KSHV K5

(A) Overlap between EBV and CMV WCL data. 7,490 proteins were quantified in either CMV experiment WCL1 or WCL2 or both (Weekes et al., 2014). 6,389 of these proteins were additionally quantified in EBV experiments WCL1–3. From this overlap, 1,083 proteins were downregulated either by EBV or CMV, of which 43 were downregulated by both viruses. This initial "non-stringent" filtering included proteins downregulated in (1) all of experiments WCL1–3, (2) any combination of two of three experiments, but not quantified in the third, and (3) any one of three experiments, but not quantified in the other two. Using "stringent" filtering, we identified a subset of ten proteins downregulated in all three EBV and both CMV experiments, that were additionally downregulated at least 2-fold more than the P3HR1 parental control, to exclude non-specific effects of tamoxifen treatment (Figure 2 and Experimental Procedures). Of the 19 proteins upregulated by both viruses, only one (APOE) met our stringent criteria.

(B) DAVID analysis identified eight of ten stringently filtered proteins functioning in poly(A) RNA binding ($p < 0.001$), of which six are known to interact (STRING database). Blue lines represent experimental evidence for physical binding, purple for catalysis, and black for reaction. Gray bars indicate evidence for co-expression.

(C) Example plots of two of the ten proteins GNL3 and TXNIP. Left: EBV experiments WCL 1–3. Right: CMV Experiment WCL2.

(legend continued on next page)

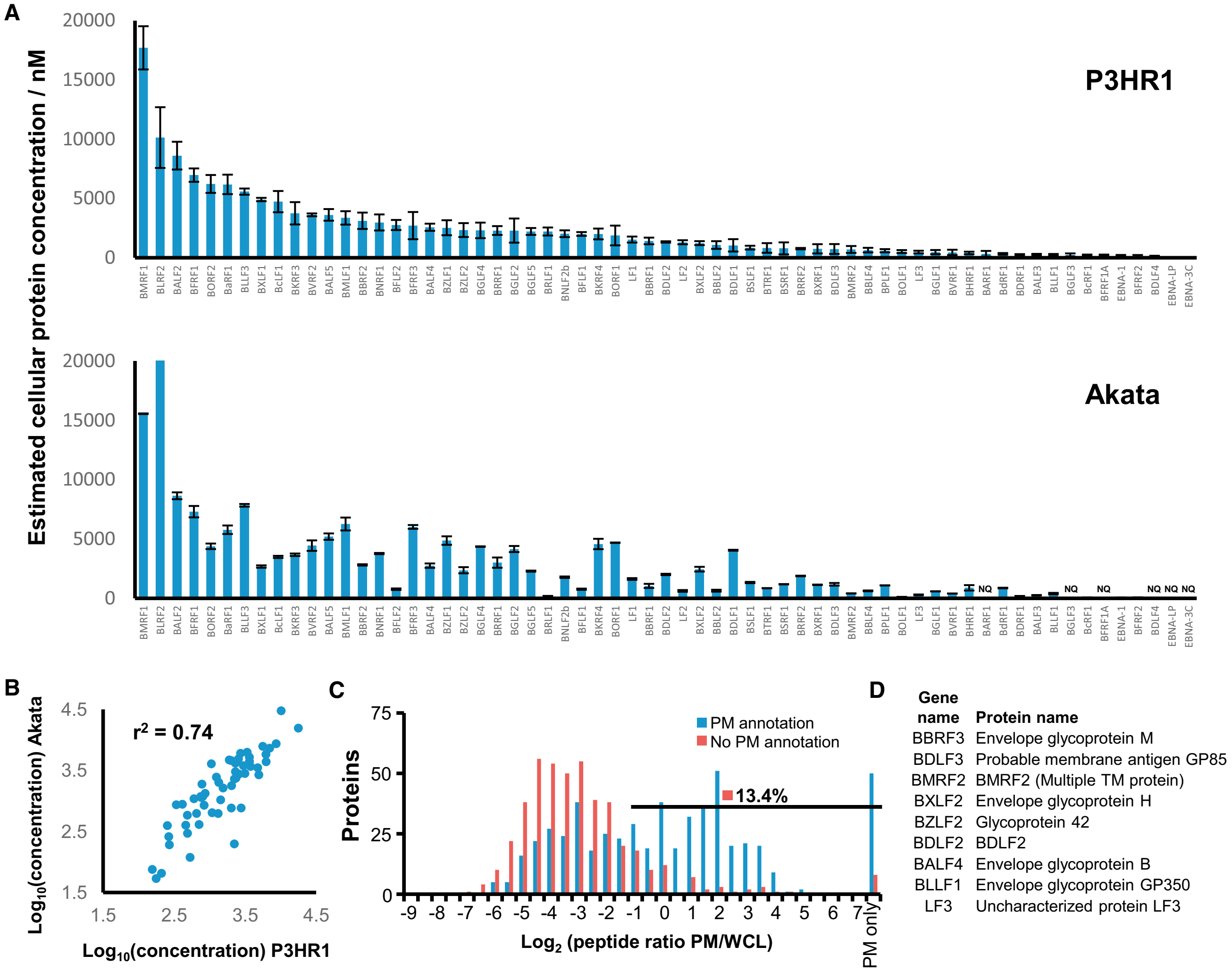

Gene name	Protein name
BBRF3	Envelope glycoprotein M
BDLF3	Probable membrane antigen GP85
BMRF2	BMRF2 (Multiple TM protein)
BXLF2	Envelope glycoprotein H
BZLF2	Glycoprotein 42
BDLF2	BDLF2
BALF4	Envelope glycoprotein B
BLLF1	Envelope glycoprotein GP350
LF3	Uncharacterized protein LF3

Figure 7. Characteristics of 63 Proteins

(A) Estimated cellular concentration of all 63 viral proteins quantified in all three P3HR1 WCL samples (top panel). A "proteomic ruler" approach was used, which uses the mass spectrometry signal of histones to scale other proteins of unknown concentration (Wiśniewski et al., 2014). Protein concentrations were similarly calculated for both Akata duplicates (bottom panel). Error bars ±SEM (top panel); ± range (bottom panel). NQ, not quantified. The concentration of BLRF2 was estimated at 29,900 nM in Akata cells; however, the y axis extends only to 20,000 for ease of comparison with P3HR1 data.

(B) Correlation between average protein concentrations estimated from P3HR1 and Akata WCL.

(C) Identification of EBV proteins at the PM. Histogram of peptide ratios for all gene ontology (GO)-annotated proteins was quantified in experiment PM1. PM only, not detected in any of experiments WCL1–3. PM annotation, plasma membrane, cell surface, extracellular, or short GO.

(D) Gene and protein names of all nine identified viral PM proteins.

from this ORF were not identified in our Akata experiment. While additional EBV genes may exist in clinical isolates, our results are consistent with the hypothesis that the majority of P3HR1 strain EBV protein-coding genes have been identified.

We recently described a method of classification in which herpesviral proteins are grouped according to their temporal profiles (Weekes et al., 2014) and used this with data from experiment WCL2 to assign EBV proteins to four temporal classes

(D) Analysis of co-regulated PM proteins. We used similar non-stringent filter as detailed in (A), comparing EBV experiment PM1 with HCMV experiments PM1 and PM2. Stringent filtering excluded non-specific effects of tamoxifen treatment only, as there was no second temporal replicate for the EBV PM experiment.

(E) 12/16 downregulated proteins met our stringent criteria, of which the top four listed functioned in synapse organization ($p < 0.05$). 46/48 upregulated proteins met our stringent criteria.

(F) Temporal proteomic analysis of NLGN4X and PCDHGC3. Left: EBV experiments WCL 1–3. Right: CMV experiment WCL 2.

(G) Coregulation of PM proteins by EBV and the KSHV K5 gene. Nine proteins met the stringent filter (D), of which three were additionally downregulated by CMV (green text) (E).

(Figures S5B–S5D). The Tp1 cluster contains multiple members of the EBV viral preinitiation complex, which activates expression of most EBV late genes from newly replicated viral DNA (Aubry et al., 2014; Djavadian et al., 2016). The Tp2 cluster included proteins important in inhibition of host innate immunity, and multiple tegument, capsid, and envelope proteins were present in the later-expressed Tp3 class.

Abundant viral proteins present at the PM may be EBV vaccine and/or therapeutic antibody targets. We used a validated filtering strategy that relies on the ratio of PM to WCL peptides (Weekes et al., 2014) to identify nine high-confidence EBV PM proteins (Figures 7C and 7D; Experimental Procedures). These included four of five EBV glycoproteins involved in B cell entry: gp350, gp42 (encoded by BZLF2), gB, and gH (Hutt-Fletcher, 2015). The single-pass type I membrane protein BDLF3, implicated in cell surface major histocompatibility complex (MHC) class I and II downregulation (Quinn et al., 2015) and cell surface heparin sulfate binding (Chesnokova et al., 2016), was present at the P3HR1 PM. We additionally detected type II membrane protein BDLF2 and the multispanning membrane protein BMRF2, which form a complex implicated in epithelial attachment and possibly intercellular spread (Gore and Hutt-Fletcher, 2009; Hutt-Fletcher, 2015). Five EBV glycoproteins were not detected, including glycoprotein gL, the secreted factor BARF1, the tegument protein BLRF2, the 7-transmbrane segment G-protein-coupled receptor BILF1, and BILF2, whose function remains unknown. Additional membrane proteins may not have been detected given their high degree of hydrophobicity, including the multipass proteins LMP1 and LMP2A.

DISCUSSION

EBV establishes latency upon B cell infection. Global studies of EBV lytic reactivation have been limited by technical hurdles, such as the ability to obtain sufficient samples for systematic proteomic and viromic analysis. Most large-scale studies have therefore focused on mRNA transcription profiles (Concha et al., 2012; Koganti et al., 2015; Traylen et al., 2015; Yuan et al., 2006), single time-point analyses of a subset of the viral proteome, or on analyses of subsets of host proteins (Koganti et al., 2015; Traylen et al., 2015). Technological advances in mass spectrometry have enabled us now to provide the most complete to date temporal study of changes in the host proteome and EBV virome during lytic B cell replication. Collectively, our study provides a valuable resource for studies of EBV lytic replication and lays the foundation for future studies in ex vivo B cells, where limiting cell quantities preclude global proteomic analysis at present.

Our analysis unexpectedly revealed that an early EBV factor targets the BCR complex for proteasomal degradation. PM and WCL proteomic analysis, as well as fluorescence-activated cell sorting (FACS) and immunofluorescence, suggests that both PM and intracellular BCR pools are targeted. Further studies are required to identify whether soluble Ig isoforms are also targeted, and to characterize the ubiquitin-proteasome pathway by which EBV downmodulates these BCR pools. One candidate is EBV BDLF3, an early factor that targets cell-surface and intracellular MHC class I molecules for ubiquitin-mediated proteasome degradation (Quinn et al., 2015).

Targeting of the BCR during EBV B cell lytic replication may be conserved across EBV strains, since we observed a similar degree of Ig loss in Akata and P3HR1 B cells, which harbor type I and II EBV, respectively. We hypothesize that EBV targets the BCR to facilitate EBV B cell replication for one of the following reasons. First, a subset of lytic B cells may produce Ig reactive to antigen present on virions. BCR degradation would then enable EBV to avoid becoming trapped by binding to these antibodies within the secretory pathway or at the cell surface. Second, EBV lytic replication is thought to occur in tonsillar plasma cells, which are loaded with Igs. Plasma cells secrete Igs at the rate of approximately 2,000 molecules per second (Hibi and Dosch, 1986). Ig degradation may therefore shift metabolic capacity away from secretion of Ig and toward production of infectious virions, in order to prevent competition for amino acid resources. Third, multiple herpesviruses encode Ig Fc-binding proteins that enable lytic cells to evade antibody responses (Hook and Friedman, 2007). Degradation of endogenous Ig may prevent an EBV-encoded Fc receptor from being fully occupied by the Fc regions of plasma cell Ig, in particular, if EBV also downregulates soluble antibody.

Additional pathways were selectively downregulated during lytic viral reactivation, including multiple components of the cell-cycle machinery. Protein downregulation in some cases may involve the EBV host shutoff protein BGLF5, which was expressed early in infection (Table S1; Figure S5B). As BGLF5 accelerates host mRNA turnover (Rowe et al., 2007), it is likely either that BGLF5 selectively destabilizes these host mRNAs, or that targeted proteins are downregulated via an alternative route, as seen for the BCR complex. EBV-encoded microRNAs (miRNAs) also suppress expression of multiple host targets (Skalsky and Cullen, 2015). An important future goal will be to systematically compare changes in mRNA and protein abundances triggered by EBV lytic replication, in order to more fully define the extent to which EBV exerts post-transcriptional effects on host target gene expression.

Multiple aspects of the EBV life cycle are intimately linked with the complement system. The EBV entry protein gp350 has homology with the C3 complement component cleavage product C3dg (Nemerow et al., 1989). gp350 and C3dg each bind to CD21, a key B cell complement receptor. The Epstein-Barr virion accelerates decay of the alternative pathway C3 convertase (Mold et al., 1988). We now report that EBV B cell lytic reactivation stimulates complement component production. Despite its recognized roles in host defense, complement components are increasingly implicated in regulation of metabolism and cellular survival. Surprisingly, intracellular roles have recently been identified for complement, including in regulation of T cell metabolic networks and homeostatic survival (Hess and Kemper, 2016). Intracellular complement roles in BCL2 and Fas upregulation have been described in T cells (Kolev et al., 2015; Lalli et al., 2008). Similarly, KSHV latency proteins exploit the complement system to promote endothelial cell survival (Lee et al., 2014). Further studies will be required to determine whether complement upregulation represents a host defense to EBV, or EBV subversion of this

immune pathway to activate metabolic or cell survival pathways that support B cell virion production.

EBV establishes latent infection upon B cell entry, and the tonsillar plasma cell population harbors lytic EBV in ex vivo studies. These observations support a model in which plasma cell differentiation provides a cue that stimulates EBV replication. Interestingly, our findings suggest that EBV lytic replication may likewise facilitate plasma cell differentiation, through suppression of TFs that maintain germinal center state and through upregulation of factors that promote plasma cell differentiation. Key germinal center B cell TFs downmodulated by EBV lytic replication included BCL6, ETS1, IRF8, ID3, and MYC, whereas the antibody secreting cell-inducing TFs IRF4, ZBTB20, FOS, and FosB were strongly upregulated. Thus, rather than passively activating as a bystander of plasma cell differentiation, EBV may play a more active role in remodeling the B cell environment to favor lytic replication.

To persist in an infected individual lifelong, herpesviruses have developed multiple strategies to modulate innate and adaptive immunity. One benefit is that we can use the overlap between proteins targeted by different herpesviruses to discover molecules particularly important in host defense. By comparing EBV, CMV, and KSHV, we identified downregulation of several members of the same protein family including Neuroligins and human leukocyte antigen (HLA) molecules. EBV and CMV both targeted Protocadherin γC3 for downregulation suggesting that this molecule, other members of the same family, and the Neuroligins might be activating NK ligands, as we previously demonstrated for Protocadherin FAT1 (Weekes et al., 2014).

The quantitation of ~80% of canonical EBV proteins in a single experiment provides a significant technological advance and enabled systematic temporal analysis of γ-herpesviral protein expression. Clues as to viral mechanism may derive from correlation of viral and cellular protein expression patterns from our temporal analysis. Early-downregulated host proteins may be targeted by one of the earliest Tp1 or Tp2 classes of viral protein. Our protein temporal classes were complementary to classical IE/E/L nomenclature and provide an increased level of resolution in combination with transcriptional data. In general, we observed low-level expression of viral proteins in gp350^{-} cells compared to gp350^{+} cells, depending on the time point studied after induction (Figure S5C). Many of the host protein changes we observed were correspondingly regulated to a lesser extent in gp350^{-} cells (Figures 2B, S2B, and 5A; Table S1), suggesting that proteins expressed in abortive lytic P3HR1 or Akata cells likely account for these changes.

Innovative strategies are required to treat EBV-related diseases. Our quantitation of the relative abundance of cell surface EBV viral proteins may enable rational design of therapeutic monoclonal antibodies for preliminary studies and enhance efforts to develop an EBV vaccine. Collectively, our studies provide a rich resource for further analyses of EBV B cell lytic replication, and herpesviral infections more widely, and identify multiple viral targets for additional analysis.

EXPERIMENTAL PROCEDURES

Brief descriptions of key experimental procedures are provided below. For complete details, see Supplemental Experimental Procedures.

WCL and PM Preparation

PM profiling was performed as previously described (Weekes et al., 2014) with minor modifications. FACS cells were washed twice with ice-cold PBS, followed by oxidation of sialic acid residues with sodium-meta-periodate. Plasma-membrane glycoproteins were selectively labeled with aminooxy-biotin. The reaction was quenched, cell numbers were normalized, and then cells were lysed in Triton X-100.

For whole-proteome samples, cells were washed, lysed in 6 M guanidine/50 mM HEPES buffer, and then vortexed, sonicated, and centrifuged. Protein concentrations were determined by bicinchoninic acid assay (BCA).

Protein Isolation and Peptide Labeling with Tandem Mass Tags

TMT-based analysis was performed as described previously (Weekes et al., 2014). Alkylated and reduced proteins were digested into peptides, which were labeled with TMT reagents, and fractions generated from combined peptide samples strong cation exchange (PM samples) or high pH reverse-phase high-performance liquid chromatography (HPLC).

Mass Spectrometry and Data Analysis

We performed mass spectrometry as described previously using an Orbitrap Fusion or Orbitrap Lumos (Weekes et al., 2014) and quantified TMT reporter ions from the MS3 scan. Peptides were identified and quantified using a Sequest-based in-house software pipeline. A combined database was searched, consisting of human, P3HR1 and Akata strains of EBV, all ORFs from six-frame translations of P3HR1 and Akata, and common contaminants. Peptide-spectral matches were filtered to a 1% false discovery rate (FDR) using linear discriminant analysis in conjunction with the target-decoy method (Huttlin et al., 2010). The resulting dataset was further collapsed to a final protein-level FDR of 1%. Protein assembly was guided by principles of parsimony. Where all peptide spectral matches (PSMs) from a given EBV protein could be explained either by a canonical gene or an ORF from the six-frame translation, the canonical gene was picked in preference. Proteins were quantified by summing TMT reporter ion counts across all matching PSM after filtering based on isolation specificity. Reverse and contaminant proteins were removed, and protein quantitation values were exported for normalization and further analysis in Excel.

Contact for Reagent and Resource Sharing

Further information and reagent requests may be directed to the corresponding authors.

ACCESSION NUMBERS

The accession number for the mass spectrometry proteomics data reported in this paper is ProteomeXchange Consortium PRIDE: PXD006317 (Vizcaíno et al., 2016).

SUPPLEMENTAL INFORMATION

Supplemental Information includes Supplemental Experimental Procedures, five figures, and four tables and can be found with this article online at http://dx.doi.org/10.1016/j.celrep.2017.04.062.

AUTHOR CONTRIBUTIONS

I.E., M.P.W., and B.E.G. designed the experiments. I.E., L.N., J.A.P., Y.N., and M.P.W. performed proteomics experiments; I.E., L.W.W., C.W.A., and C.J. performed biochemical experiments; and I.E., L.N., M.P.W., and B.E.G wrote the manuscript. L.N., L.S., J.A.P., S.P.G., and M.P.W. analyzed the proteomics data, and N.E.G. provided EBV genome sequence data and bioinformatic support. Y.M. and N.E.G. performed additional bioinformatic analyses; E.K. provided helpful scientific discussions; and I.E., L.N., S.P.G., M.P.W., E.K., and B.E.G. edited the manuscript. M.P.W. and B.E.G. supervised all research.

ACKNOWLEDGMENTS

This work was supported by a Burroughs Wellcome Career Award in Medical Sciences and KO8 NCI CA140780 to B.E.G., a Wellcome Trust Senior Clinical

Research Fellowship (108070/Z/15/Z) to M.P.W., a strategic award to Cambridge Institute for Medical Research from the Wellcome Trust (100140), NCI RO1 CA085180 to E.K., NIH GM67945 to S.P.G., K01 DK098285 to J.A.P., and a National Science Scholarship (PhD) from A*STAR to L.W.W. We thank Bo Zhao for discussion and review of the manuscript, Eric Johannsen for the P3HR1-ZHT/RHT cells and for scientific discussions, Makoto Ohashi and Stephen Trudeau for technical assistance, and Yagnesh Umrania for assistance with database generation.

Received: December 22, 2016
Revised: March 24, 2017
Accepted: April 20, 2017
Published: May 16, 2017

REFERENCES

Adamson, A.L., Le, B.T., and Siedenburg, B.D. (2014). Inhibition of mTORC1 inhibits lytic replication of Epstein-Barr virus in a cell-type specific manner. Virol. J. *11*, 110.

Arvey, A., Tempera, I., Tsai, K., Chen, H.S., Tikhmyanova, N., Klichinsky, M., Leslie, C., and Lieberman, P.M. (2012). An atlas of the Epstein-Barr virus transcriptome and epigenome reveals host-virus regulatory interactions. Cell Host Microbe *12*, 233–245.

Aubry, V., Mure, F., Mariamé, B., Deschamps, T., Wyrwicz, L.S., Manet, E., and Gruffat, H. (2014). Epstein-Barr virus late gene transcription depends on the assembly of a virus-specific preinitiation complex. J. Virol. *88*, 12825–12838.

Balachandran, N., Pittari, J., and Hutt-Fletcher, L.M. (1986). Detection by monoclonal antibodies of an early membrane protein induced by Epstein-Barr virus. J. Virol. *60*, 369–375.

Blaszczyk, K., Nowicka, H., Kostyrko, K., Antonczyk, A., Wesoly, J., and Bluyssen, H.A. (2016). The unique role of STAT2 in constitutive and IFN-induced transcription and antiviral responses. Cytokine Growth Factor Rev. *29*, 71–81.

Calderwood, M.A., Holthaus, A.M., and Johannsen, E. (2008). The Epstein-Barr virus LF2 protein inhibits viral replication. J. Virol. *82*, 8509–8519.

Cen, O., and Longnecker, R. (2015). Latent Membrane Protein 2 (LMP2). Curr. Top. Microbiol. Immunol. *391*, 151–180.

Chesnokova, L.S., Valencia, S.M., and Hutt-Fletcher, L.M. (2016). The BDLF3 gene product of Epstein-Barr virus, gp150, mediates non-productive binding to heparan sulfate on epithelial cells and only the binding domain of CD21 is required for infection. Virology *494*, 23–28.

Chien, Y.C., Chen, J.Y., Liu, M.Y., Yang, H.I., Hsu, M.M., Chen, C.J., and Yang, C.S. (2001). Serologic markers of Epstein-Barr virus infection and nasopharyngeal carcinoma in Taiwanese men. N. Engl. J. Med. *345*, 1877–1882.

Chiu, Y.F., and Sugden, B. (2016). Epstein-Barr virus: The path from latent to productive infection. Annu. Rev. Virol. *3*, 359–372.

Chiu, Y.F., Sugden, A.U., and Sugden, B. (2013). Epstein-Barr viral productive amplification reprograms nuclear architecture, DNA replication, and histone deposition. Cell Host Microbe *14*, 607–618.

Cohen, J.I., Fauci, A.S., Varmus, H., and Nabel, G.J. (2011). Epstein-Barr virus: An important vaccine target for cancer prevention. Sci. Transl. Med. *3*, 107fs7.

Colby, B.M., Shaw, J.E., Elion, G.B., and Pagano, J.S. (1980). Effect of acyclovir [9-(2-hydroxyethoxymethyl)guanine] on Epstein-Barr virus DNA replication. J. Virol. *34*, 560–568.

Concha, M., Wang, X., Cao, S., Baddoo, M., Fewell, C., Lin, Z., Hulme, W., Hedges, D., McBride, J., and Flemington, E.K. (2012). Identification of new viral genes and transcript isoforms during Epstein-Barr virus reactivation using RNA-seq. J. Virol. *86*, 1458–1467.

Cox, J., and Mann, M. (2008). MaxQuant enables high peptide identification rates, individualized p.p.b.-range mass accuracies and proteome-wide protein quantification. Nat. Biotechnol. *26*, 1367–1372.

Djavadian, R., Chiu, Y.F., and Johannsen, E. (2016). An Epstein-Barr virus-encoded protein complex requires an origin of lytic replication in cis to mediate late gene transcription. PLoS Pathog. *12*, e1005718.

Dorrello, N.V., Peschiaroli, A., Guardavaccaro, D., Colburn, N.H., Sherman, N.E., and Pagano, M. (2006). S6K1- and betaTRCP-mediated degradation of PDCD4 promotes protein translation and cell growth. Science *314*, 467–471.

Duan, S., Cermak, L., Pagan, J.K., Rossi, M., Martinengo, C., di Celle, P.F., Chapuy, B., Shipp, M., Chiarle, R., and Pagano, M. (2012). FBXO11 targets BCL6 for degradation and is inactivated in diffuse large B-cell lymphomas. Nature *481*, 90–93.

Feighny, R.J., Henry, B.E., 2nd, and Pagano, J.S. (1981). Epstein-Barr virus polypeptides: Effect of inhibition of viral DNA replication on their synthesis. J. Virol. *37*, 61–71.

Gore, M., and Hutt-Fletcher, L.M. (2009). The BDLF2 protein of Epstein-Barr virus is a type II glycosylated envelope protein whose processing is dependent on coexpression with the BMRF2 protein. Virology *383*, 162–167.

Grayson, S., Johnson-Winegar, A.G., Wintroub, B.U., Isseroff, R.R., Epstein, E.H., Jr., and Elias, P.M. (1985). Lamellar body-enriched fractions from neonatal mice: Preparative techniques and partial characterization. J. Invest. Dermatol. *85*, 289–294.

Hammerschmidt, W., and Sugden, B. (2013). Replication of Epstein-Barr viral DNA. Cold Spring Harb. Perspect. Biol. *5*, a013029.

Havens, C.G., and Walter, J.C. (2011). Mechanism of CRL4(Cdt2), a PCNA-dependent E3 ubiquitin ligase. Genes Dev. *25*, 1568–1582.

Hess, C., and Kemper, C. (2016). Complement-mediated regulation of metabolism and basic cellular processes. Immunity *45*, 240–254.

Hibi, T., and Dosch, H.M. (1986). Limiting dilution analysis of the B cell compartment in human bone marrow. Eur. J. Immunol. *16*, 139–145.

Hook, L.M., and Friedman, H.M. (2007). Subversion of innate and adaptive immunity: Immune evasion from antibody and complement. In Human Herpesviruses: Biology, Therapy, and Immunoprophylaxis, A. Arvin, G. Campadelli-Fiume, E. Mocarski, P.S. Moore, B. Roizman, R. Whitley, and K. Yamanishi, eds. (Cambridge), pp. 1137–1150.

Huang, W., Sherman, B.T., and Lempicki, R.A. (2009). Systematic and integrative analysis of large gene lists using DAVID bioinformatics resources. Nat. Protoc. *4*, 44–57.

Hutt-Fletcher, L.M. (2015). EBV glycoproteins: Where are we now? Future Virol. *10*, 1155–1162.

Huttlin, E.L., Jedrychowski, M.P., Elias, J.E., Goswami, T., Rad, R., Beausoleil, S.A., Villén, J., Haas, W., Sowa, M.E., and Gygi, S.P. (2010). A tissue-specific atlas of mouse protein phosphorylation and expression. Cell *143*, 1174–1189.

Kenney, S.C. (2007). Reactivation and lytic replication of EBV. In Human Herpesviruses: Biology, Therapy, and Immunoprophylaxis, A. Arvin, G. Campadelli-Fiume, E. Mocarski, P.S. Moore, B. Roizman, R. Whitley, and K. Yamanishi, eds. (Cambridge).

Kenney, S.C., and Mertz, J.E. (2014). Regulation of the latent-lytic switch in Epstein-Barr virus. Semin. Cancer Biol. *26*, 60–68.

Kieff, E., and Rickinson, A. (2007). Epstein-Barr virus and its replication. In Fields Virology, D.M. Knipe and P.M. Howley, eds. (Lippincott), pp. 2603–2654.

Klein, G., Sugden, B., Leibold, W., and Menezes, J. (1974). Infection of EBV-genome-negative and -positive human lymphoblastoid cell lines with biologically different preparations of EBV. Intervirology *3*, 232–244.

Koganti, S., Clark, C., Zhi, J., Li, X., Chen, E.I., Chakrabortty, S., Hill, E.R., and Bhaduri-McIntosh, S. (2015). Cellular STAT3 functions via PCBP2 to restrain Epstein-Barr virus lytic activation in B lymphocytes. J. Virol. *89*, 5002–5011.

Kolev, M., Dimeloe, S., Le Friec, G., Navarini, A., Arbore, G., Povoleri, G.A., Fischer, M., Belle, R., Loeliger, J., Develioglu, L., et al. (2015). Complement regulates nutrient influx and metabolic reprogramming during Th1 cell responses. Immunity *42*, 1033–1047.

Laichalk, L.L., and Thorley-Lawson, D.A. (2005). Terminal differentiation into plasma cells initiates the replicative cycle of Epstein-Barr virus in vivo. J. Virol. *79*, 1296–1307.

Lalli, P.N., Strainic, M.G., Yang, M., Lin, F., Medof, M.E., and Heeger, P.S. (2008). Locally produced C5a binds to T cell-expressed C5aR to enhance

effector T-cell expansion by limiting antigen-induced apoptosis. Blood *112*, 1759–1766.

Lee, M.S., Jones, T., Song, D.Y., Jang, J.H., Jung, J.U., and Gao, S.J. (2014). Exploitation of the complement system by oncogenic Kaposi's sarcoma-associated herpesvirus for cell survival and persistent infection. PLoS Pathog. *10*, e1004412.

Lin, J.C., and Raab-Traub, N. (1987). Two strains of Epstein-Barr virus (B95-8 and a P3HR-1 subclone) that lack defective genomes induce early antigen and cause abortive infection of Raji cells. J. Virol. *61*, 1985–1991.

Ma, S.D., Hegde, S., Young, K.H., Sullivan, R., Rajesh, D., Zhou, Y., Jankowska-Gan, E., Burlingham, W.J., Sun, X., Gulley, M.L., et al. (2011). A new model of Epstein-Barr virus infection reveals an important role for early lytic viral protein expression in the development of lymphomas. J. Virol. *85*, 165–177.

Matheson, C.J., Backos, D.S., and Reigan, P. (2016). Targeting WEE1 Kinase in Cancer. Trends Pharmacol. Sci. *37*, 872–881.

McKenzie, J., and El-Guindy, A. (2015). Epstein-Barr virus lytic cycle reactivation. Curr. Top. Microbiol. Immunol. *391*, 237–261.

Miller, G., Robinson, J., Heston, L., and Lipman, M. (1974). Differences between laboratory strains of Epstein-Barr virus based on immortalization, abortive infection, and interference. Proc. Natl. Acad. Sci. USA *71*, 4006–4010.

Mold, C., Bradt, B.M., Nemerow, G.R., and Cooper, N.R. (1988). Epstein-Barr virus regulates activation and processing of the third component of complement. J. Exp. Med. *168*, 949–969.

Moody, C.A., Scott, R.S., Amirghahari, N., Nathan, C.O., Young, L.S., Dawson, C.W., and Sixbey, J.W. (2005). Modulation of the cell growth regulator mTOR by Epstein-Barr virus-encoded LMP2A. J. Virol. *79*, 5499–5506.

Nemerow, G.R., Houghten, R.A., Moore, M.D., and Cooper, N.R. (1989). Identification of an epitope in the major envelope protein of Epstein-Barr virus that mediates viral binding to the B lymphocyte EBV receptor (CR2). Cell *56*, 369–377.

Nutt, S.L., Hodgkin, P.D., Tarlinton, D.M., and Corcoran, L.M. (2015). The generation of antibody-secreting plasma cells. Nat. Rev. Immunol. *15*, 160–171.

Paladino, P., Marcon, E., Greenblatt, J., and Frappier, L. (2014). Identification of herpesvirus proteins that contribute to G1/S arrest. J. Virol. *88*, 4480–4492.

Palser, A.L., Grayson, N.E., White, R.E., Corton, C., Correia, S., Ba Abdullah, M.M., Watson, S.J., Cotten, M., Arrand, J.R., Murray, P.G., et al. (2015). Genome diversity of Epstein-Barr virus from multiple tumor types and normal infection. J. Virol. *89*, 5222–5237.

Powles, T., Robinson, D., Stebbing, J., Shamash, J., Nelson, M., Gazzard, B., Mandelia, S., Møller, H., and Bower, M. (2009). Highly active antiretroviral therapy and the incidence of non-AIDS-defining cancers in people with HIV infection. J. Clin. Oncol. *27*, 884–890.

Quinn, L.L., Williams, L.R., White, C., Forrest, C., Zuo, J., and Rowe, M. (2015). The missing link in Epstein-Barr virus immune evasion: The BDLF3 gene induces ubiquitination and downregulation of major histocompatibility complex class I (MHC-I) and MHC-II. J. Virol. *90*, 356–367.

Rathinam, V.A., Jiang, Z., Waggoner, S.N., Sharma, S., Cole, L.E., Waggoner, L., Vanaja, S.K., Monks, B.G., Ganesan, S., Latz, E., et al. (2010). The AIM2 inflammasome is essential for host defense against cytosolic bacteria and DNA viruses. Nat. Immunol. *11*, 395–402.

Rowe, M., Glaunsinger, B., van Leeuwen, D., Zuo, J., Sweetman, D., Ganem, D., Middeldorp, J., Wiertz, E.J., and Ressing, M.E. (2007). Host shutoff during productive Epstein-Barr virus infection is mediated by BGLF5 and may contribute to immune evasion. Proc. Natl. Acad. Sci. USA *104*, 3366–3371.

Schoggins, J.W., Wilson, S.J., Panis, M., Murphy, M.Y., Jones, C.T., Bieniasz, P., and Rice, C.M. (2011). A diverse range of gene products are effectors of the type I interferon antiviral response. Nature *472*, 481–485.

Schreiner, S., and Wodrich, H. (2013). Virion factors that target Daxx to overcome intrinsic immunity. J. Virol. *87*, 10412–10422.

Skalsky, R.L., and Cullen, B.R. (2015). EBV noncoding RNAs. Curr. Top. Microbiol. Immunol. *391*, 181–217.

Subramanian, A., Tamayo, P., Mootha, V.K., Mukherjee, S., Ebert, B.L., Gillette, M.A., Paulovich, A., Pomeroy, S.L., Golub, T.R., Lander, E.S., and Mesirov, J.P. (2005). Gene set enrichment analysis: A knowledge-based approach for interpreting genome-wide expression profiles. Proc. Natl. Acad. Sci. USA *102*, 15545–15550.

Takada, K., and Ono, Y. (1989). Synchronous and sequential activation of latently infected Epstein-Barr virus genomes. J. Virol. *63*, 445–449.

Taniguchi, T., Ogasawara, K., Takaoka, A., and Tanaka, N. (2001). IRF family of transcription factors as regulators of host defense. Annu. Rev. Immunol. *19*, 623–655.

Thorley-Lawson, D.A. (2015). EBV persistence—introducing the virus. Curr. Top. Microbiol. Immunol. *390*, 151–209.

Timms, R.T., Duncan, L.M., Tchasovnikarova, I.A., Antrobus, R., Smith, D.L., Dougan, G., Weekes, M.P., and Lehner, P.J. (2013). Haploid genetic screens identify an essential role for PLP2 in the downregulation of novel plasma membrane targets by viral E3 ubiquitin ligases. PLoS Pathog. *9*, e1003772.

Traylen, C., Ramasubramanyan, S., Zuo, J., Rowe, M., Almohammad, R., Heesom, K., Sweet, S.M., Matthews, D.A., and Sinclair, A.J. (2015). Identification of Epstein-Barr virus replication proteins in Burkitt's lymphoma cells. Pathogens *4*, 739–751.

Tsai, M.H., Raykova, A., Klinke, O., Bernhardt, K., Gärtner, K., Leung, C.S., Geletneky, K., Sertel, S., Münz, C., Feederle, R., and Delecluse, H.J. (2013). Spontaneous lytic replication and epitheliotropism define an Epstein-Barr virus strain found in carcinomas. Cell Rep. *5*, 458–470.

Tugizov, S.M., Herrera, R., and Palefsky, J.M. (2013). Epstein-Barr virus transcytosis through polarized oral epithelial cells. J. Virol. *87*, 8179–8194.

Verma, D., Ling, C., Johannsen, E., Nagaraja, T., and Swaminathan, S. (2009). Negative autoregulation of Epstein-Barr virus (EBV) replicative gene expression by EBV SM protein. J. Virol. *83*, 8041–8050.

Vizcaíno, J.A., Csordas, A., del-Toro, N., Dianes, J.A., Griss, J., Lavidas, I., Mayer, G., Perez-Riverol, Y., Reisinger, F., Ternent, T., et al. (2016). 2016 update of the PRIDE database and its related tools. Nucleic Acids Res. *44* (*D1*), D447–D456.

Weekes, M.P., Tomasec, P., Huttlin, E.L., Fielding, C.A., Nusinow, D., Stanton, R.J., Wang, E.C., Aicheler, R., Murrell, I., Wilkinson, G.W., et al. (2014). Quantitative temporal viromics: An approach to investigate host-pathogen interaction. Cell *157*, 1460–1472.

Williams, S.A., Maecker, H.L., French, D.M., Liu, J., Gregg, A., Silverstein, L.B., Cao, T.C., Carano, R.A., and Dixit, V.M. (2011). USP1 deubiquitinates ID proteins to preserve a mesenchymal stem cell program in osteosarcoma. Cell *146*, 918–930.

Williams, L.R., Quinn, L.L., Rowe, M., and Zuo, J. (2015). Induction of the lytic cycle sensitizes epstein-barr virus-infected B cells to NK cell killing that is counteracted by virus-mediated NK cell evasion mechanisms in the late lytic cycle. J. Virol. *90*, 947–958.

Wiśniewski, J.R., Hein, M.Y., Cox, J., and Mann, M. (2014). A ''proteomic ruler'' for protein copy number and concentration estimation without spike-in standards. Mol. Cell. Proteomics *13*, 3497–3506.

Yang, H.S., Jansen, A.P., Komar, A.A., Zheng, X., Merrick, W.C., Costes, S., Lockett, S.J., Sonenberg, N., and Colburn, N.H. (2003). The transformation suppressor Pdcd4 is a novel eukaryotic translation initiation factor 4A binding protein that inhibits translation. Mol. Cell. Biol. *23*, 26–37.

Yuan, J., Cahir-McFarland, E., Zhao, B., and Kieff, E. (2006). Virus and cell RNAs expressed during Epstein-Barr virus replication. J. Virol. *80*, 2548–2565.

Cell Reports, Volume *19*

Supplemental Information

A Temporal Proteomic Map of Epstein-Barr Virus Lytic Replication in B Cells

Ina Ersing, Luis Nobre, Liang Wei Wang, Lior Soday, Yijie Ma, Joao A. Paulo, Yohei Narita, Camille W. Ashbaugh, Chang Jiang, Nicholas E. Grayson, Elliott Kieff, Steven P. Gygi, Michael P. Weekes, and Benjamin E. Gewurz

Figure S1

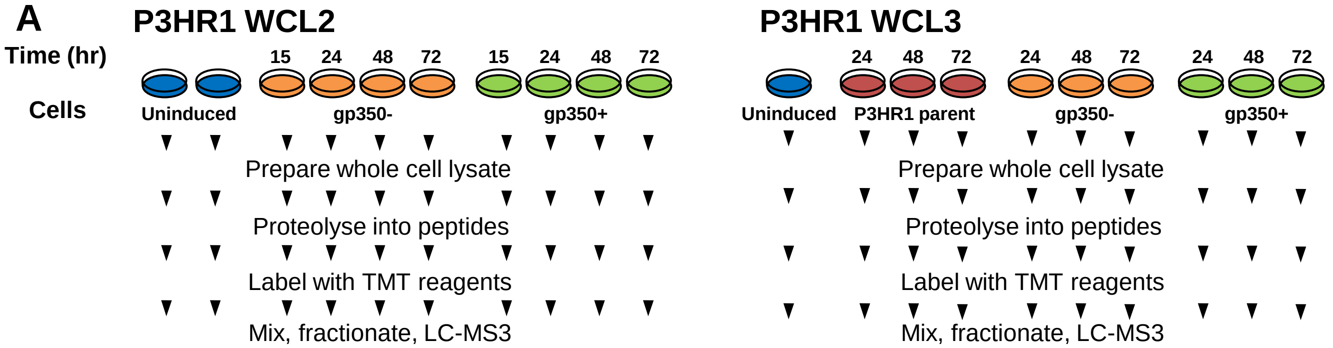

B

	P3HR1					Akata	P3HR1
	WCL1	WCL2	WCL3	Any WCL	All WCL	WCL	PM1
**All peptides**	89031	109497	95648			70311	5244
**Human proteins**	7493	7045	7382	8249	6307	6982	1012
**EBV proteins**	67	68	65	69	63	59	18

C

	1_P3HR1 parent Un	1_P3HR1 parent 24h	1_P3HR1 parent 72h	3_P3HR1 parent 24h	3_P3HR1 parent 48h	3_P3HR1 parent 72h	1_gp350- 24h	1_gp350- 48h	1_gp350- 72h	2_gp350- 15h	2_gp350- 24h	2_gp350- 48h	2_gp350- 72h	3_gp350- 24h	3_gp350- 48h	3_gp350- 72h	1_gp350+ 24h	1_gp350+ 48h	1_gp350+ 72h	2_gp350+ 15h	2_gp350+ 24h	2_gp350+ 48h	2_gp350+ 72h	3_gp350+ 24h	3_gp350+ 48h	3_gp350+ 72h
1_P3HR1 parent Un		0.85	0.83	0.50	0.44	0.49	0.35	0.17	0.15	0.05	0.07	0.04	-0.02	0.08	0.08	0.09	0.23	0.28	0.16	0.13	0.13	0.13	0.10	0.10	0.14	0.13
1_P3HR1 parent 24h	0.85		0.79	0.53	0.41	0.46	0.23	0.15	-0.02	0.12	0.09	0.04	0.04	0.02	0.00	0.07	0.15	0.15	0.05	0.12	0.11	0.12	0.12	0.00	0.07	0.07
1_P3HR1 parent 72h	0.83	0.79		0.50	0.51	0.50	0.19	0.01	0.09	0.05	0.02	0.00	0.01	0.00	-0.01	-0.03	0.08	0.14	0.05	0.04	0.04	0.06	0.07	0.01	0.05	0.04
3_P3HR1 parent 24h	0.50	0.53	0.50		0.78	0.90	-0.01	0.03	-0.11	0.05	0.01	-0.08	0.11	0.02	0.04	-0.08	-0.04	-0.03	-0.09	-0.01	-0.03	-0.03	0.05	0.00	0.01	0.04
3_P3HR1 parent 48h	0.44	0.41	0.51	0.78		0.85	0.11	0.09	0.09	0.07	0.08	0.13	0.27	0.24	0.31	0.13	0.17	0.19	0.20	0.19	0.18	0.20	0.24	0.30	0.31	0.27
3_P3HR1 parent 72h	0.49	0.46	0.50	0.90	0.85		0.10	0.12	0.04	0.07	0.05	-0.05	0.14	0.15	0.23	0.03	0.14	0.15	0.13	0.12	0.10	0.07	0.11	0.23	0.21	0.23
1_gp350- 24h	0.35	0.23	0.19	-0.01	0.11	0.10		0.66	0.67	0.37	0.34	0.27	0.21	0.39	0.52	0.41	0.75	0.75	0.73	0.55	0.56	0.46	0.35	0.48	0.47	0.55
1_gp350- 48h	0.17	0.15	0.01	0.03	0.09	0.12	0.66		0.54	0.16	0.20	0.15	0.25	0.33	0.45	0.37	0.59	0.65	0.61	0.35	0.35	0.31	0.28	0.41	0.41	0.48
1_gp350- 72h	0.15	-0.02	0.09	-0.11	0.09	0.04	0.67	0.54		0.00	0.11	0.20	0.09	0.40	0.52	0.48	0.51	0.57	0.75	0.23	0.26	0.23	0.17	0.39	0.39	0.41
2_gp350- 15h	0.05	0.12	0.05	0.05	0.07	0.07	0.37	0.16	0.00		0.67	0.52	0.53	0.24	0.26	0.15	0.37	0.26	0.20	0.73	0.70	0.60	0.51	0.27	0.26	0.29
2_gp350- 24h	0.07	0.09	0.02	0.01	0.08	0.05	0.34	0.20	0.11	0.67		0.48	0.48	0.30	0.32	0.25	0.37	0.31	0.26	0.64	0.60	0.59	0.44	0.30	0.32	0.30
2_gp350- 48h	0.04	0.04	0.00	-0.08	0.13	-0.05	0.27	0.15	0.20	0.52	0.48		0.54	0.61	0.56	0.62	0.29	0.28	0.26	0.58	0.66	0.80	0.68	0.34	0.45	0.25
2_gp350- 72h	-0.02	0.04	0.01	0.11	0.27	0.14	0.21	0.25	0.09	0.53	0.48	0.54		0.23	0.33	0.18	0.26	0.28	0.21	0.53	0.53	0.60	0.72	0.30	0.34	0.33
3_gp350- 24h	0.08	0.02	0.00	0.02	0.24	0.15	0.39	0.33	0.40	0.24	0.30	0.61	0.23		0.90	0.79	0.46	0.49	0.51	0.50	0.55	0.59	0.47	0.68	0.71	0.51
3_gp350- 48h	0.08	0.00	-0.01	0.04	0.31	0.23	0.52	0.45	0.52	0.26	0.32	0.56	0.33	0.90		0.78	0.63	0.64	0.69	0.60	0.63	0.64	0.54	0.83	0.85	0.71
3_gp350- 72h	0.09	0.07	-0.03	-0.08	0.13	0.03	0.41	0.37	0.48	0.15	0.25	0.62	0.18	0.79	0.78		0.35	0.38	0.44	0.35	0.41	0.49	0.38	0.48	0.56	0.40
1_gp350+ 24h	0.23	0.15	0.08	-0.04	0.17	0.14	0.75	0.59	0.51	0.37	0.37	0.29	0.26	0.46	0.63	0.35		0.92	0.89	0.76	0.75	0.65	0.47	0.80	0.74	0.82
1_gp350+ 48h	0.28	0.15	0.14	-0.03	0.19	0.15	0.75	0.65	0.57	0.26	0.31	0.28	0.28	0.49	0.64	0.38	0.92		0.90	0.69	0.69	0.63	0.51	0.79	0.77	0.83
1_gp350+ 72h	0.16	0.05	0.05	-0.09	0.20	0.13	0.73	0.61	0.75	0.20	0.26	0.26	0.21	0.51	0.69	0.44	0.89	0.90		0.62	0.63	0.54	0.41	0.78	0.73	0.80
2_gp350+ 15h	0.13	0.12	0.04	-0.01	0.19	0.12	0.55	0.35	0.23	0.73	0.64	0.58	0.53	0.50	0.60	0.35	0.76	0.69	0.62		0.96	0.88	0.72	0.72	0.70	0.70
2_gp350+ 24h	0.13	0.11	0.04	-0.03	0.18	0.10	0.56	0.35	0.26	0.70	0.60	0.66	0.53	0.55	0.63	0.41	0.75	0.69	0.63	0.96		0.91	0.73	0.71	0.70	0.68
2_gp350+ 48h	0.13	0.12	0.06	-0.03	0.20	0.07	0.46	0.31	0.23	0.60	0.59	0.80	0.60	0.59	0.64	0.49	0.65	0.63	0.54	0.88	0.91		0.83	0.66	0.73	0.62
2_gp350+ 72h	0.10	0.12	0.07	0.05	0.24	0.11	0.35	0.28	0.17	0.51	0.44	0.68	0.72	0.47	0.54	0.38	0.47	0.51	0.41	0.72	0.73	0.83		0.53	0.68	0.50
3_gp350+ 24h	0.10	0.00	0.01	0.00	0.30	0.23	0.48	0.41	0.39	0.27	0.30	0.34	0.30	0.68	0.83	0.48	0.80	0.79	0.78	0.72	0.71	0.66	0.53		0.93	0.90
3_gp350+ 48h	0.14	0.07	0.05	0.01	0.31	0.21	0.47	0.41	0.39	0.26	0.32	0.45	0.34	0.71	0.85	0.56	0.74	0.77	0.73	0.70	0.70	0.73	0.68	0.93		0.85
3_gp350+ 72h	0.13	0.07	0.04	0.04	0.27	0.23	0.55	0.48	0.41	0.29	0.30	0.25	0.33	0.51	0.71	0.40	0.82	0.83	0.80	0.70	0.68	0.62	0.50	0.90	0.85	

Pearson correlation
-0.11
0.96

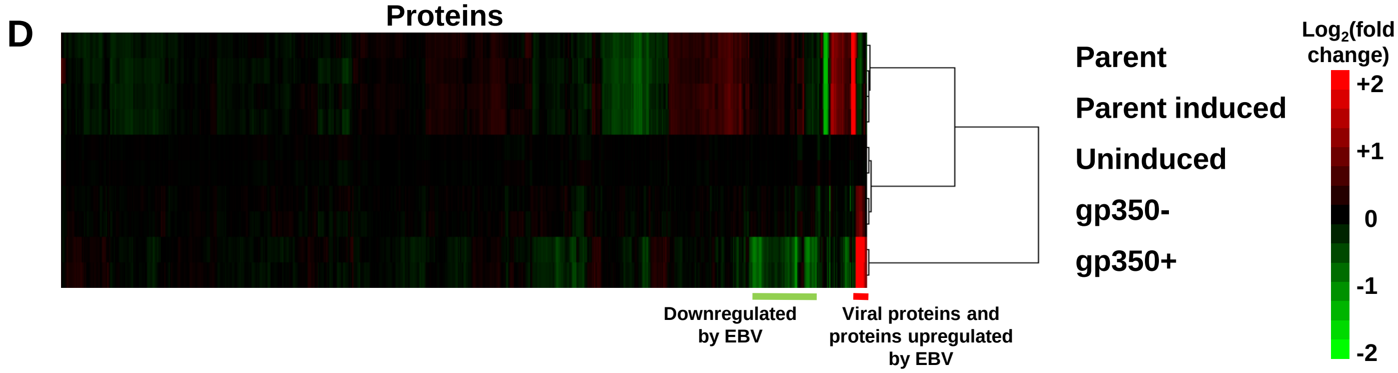

Figure S1. Related to **Figure 1**: Temporal proteomic profiling of EBV infection in B-cells.

A. Workflow of P3HR1 experiments WCL2 and WCL3. ZHT/RHT or parental control P3HR1 cells were treated with 4-HT for the indicated time.

B. Peptides and proteins quantified in all experiments in this manuscript. Of 1012 proteins quantified in P3HR1 experiment PM1, 550 were annotated by Gene Ontology 'plasma membrane', 'cell surface', 'extracellular' or 'short GO' (see Supplemental Experimental Procedures).

C. Matrix of Pearson correlations for P3HR1 data. For every sample in each of the P3HR1 WCL1-3 series, fold change was calculated compared to the unstimulated P3HR1-ZHT/RHT control (indicated in dark blue in Figures 1A, S1A) and then correlated to the fold change of every other sample. For the WCL2 series, the average of the two unstimulated controls was used in the calculation. Particularly strong correlations were seen between replicated gp350+ samples (lower right box) and replicate parental controls (upper left box).

D. Hierarchical cluster analysis of proteins quantified in the Akata WCL experiment (EBV-negative parent untreated or anti-IgG cross-linked for 48 hours, and EBV+ uninduced or gp350- versus gp350+ cells following 48 hour of anti-IgG crosslinking) demonstrated that biological duplicates clustered together. Fold change was calculated for each value compared to the average of the two uninduced samples. Proteins were filtered to be quantified by a minimum of 2 peptides.

Figure S2

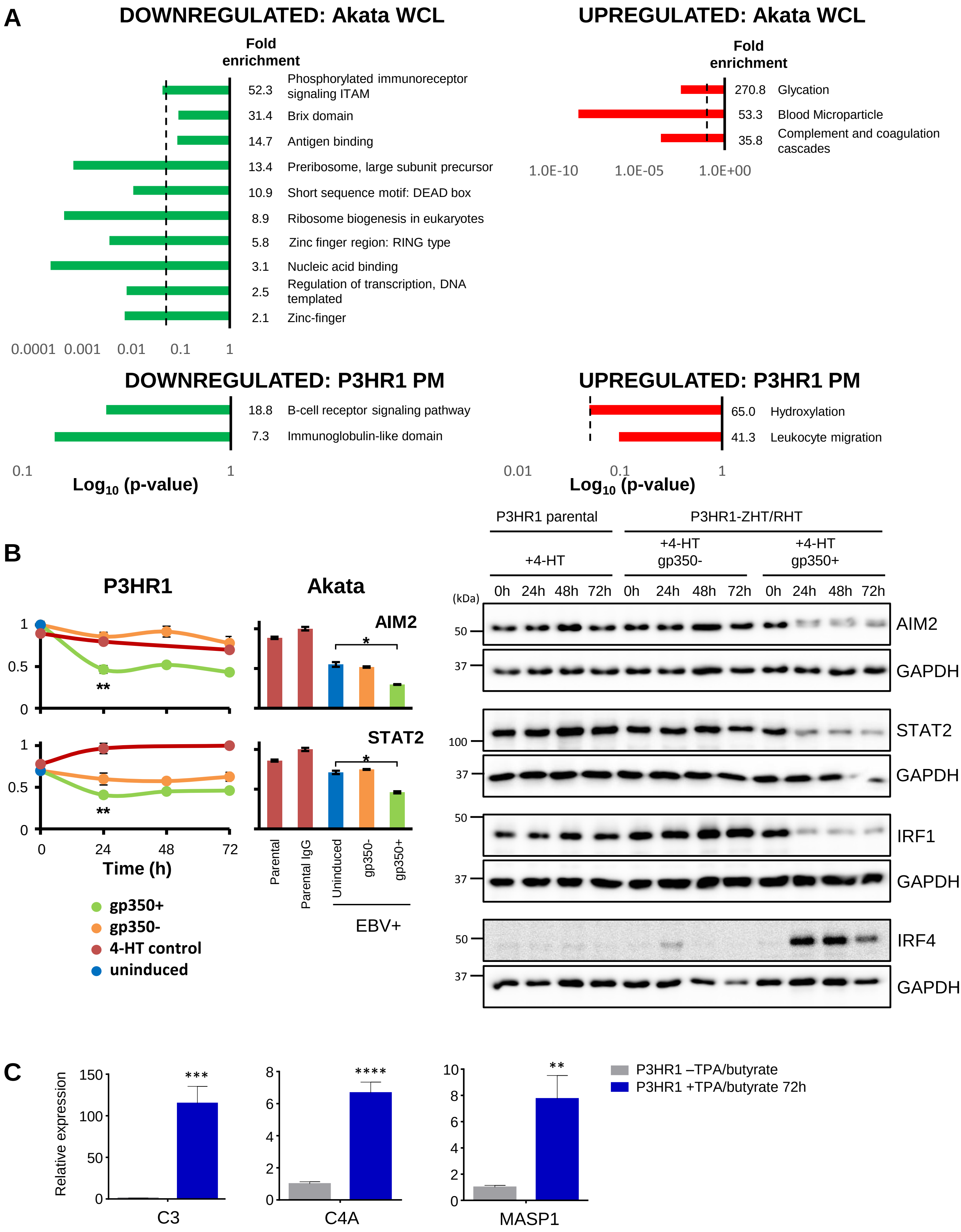
A
DOWNREGULATED: Akata WCL
Fold enrichment
52.3 Phosphorylated immunoreceptor signaling ITAM
31.4 Brix domain
14.7 Antigen binding
13.4 Preribosome, large subunit precursor
10.9 Short sequence motif: DEAD box
8.9 Ribosome biogenesis in eukaryotes
5.8 Zinc finger region: RING type
3.1 Nucleic acid binding
2.5 Regulation of transcription, DNA templated
2.1 Zinc-finger
0.0001 0.001 0.01 0.1 1
UPREGULATED: Akata WCL
Fold enrichment
270.8 Glycation
53.3 Blood Microparticle
35.8 Complement and coagulation cascades
1.0E-10 1.0E-05 1.0E+00
DOWNREGULATED: P3HR1 PM
18.8 B-cell receptor signaling pathway
7.3 Immunoglobulin-like domain
0.1 1
Log10 (p-value)
UPREGULATED: P3HR1 PM
65.0 Hydroxylation
41.3 Leukocyte migration
0.01 0.1 1
Log10 (p-value)
B
P3HR1
Akata
AIM2
STAT2
Time (h)
gp350+
gp350-
4-HT control
uninduced
Parental
Parental IgG
Uninduced
gp350-
gp350+
EBV+
P3HR1 parental
P3HR1-ZHT/RHT
+4-HT
+4-HT gp350-
+4-HT gp350+
0h 24h 48h 72h 0h 24h 48h 72h 0h 24h 48h 72h
(kDa)
AIM2
GAPDH
STAT2
GAPDH
IRF1
GAPDH
IRF4
GAPDH
C
Relative expression
C3
C4A
MASP1
P3HR1 –TPA/butyrate
P3HR1 +TPA/butyrate 72h

Figure S2. Related to **Figure 2**: EBV lytic replication induces complement pathway component expression in B-cells, and regulates components of the interferon pathway.

A. Functional enrichment within all proteins that were significantly ($p<0.075$) down or up-regulated an average of >2-fold by lytic EBV replication in Akata cells (red and orange dots in Figure 1B, right panel). A background of all quantified proteins was used. A similar analysis was performed for proteins down- or upregulated >2-fold in P3HR1 experiment PM1, with $p<0.1$ (red and orange dots in Figure 1C). Dotted lines: $p=0.05$.

B. Downregulation of AIM2 and STAT2 by EBV lytic replication, with validation by immunoblot. Also shown are immunoblots of P3HR1 whole cell extracts for IRF1 down-modulation and up-regulation of IRF4 (Table S1). STAT2 immunoblot was performed on the same membrane as CD79A in Figure 3A.

C. qRT-PCR identification of complement transcript upregulation in induced P3HR1 cells induced by TPA and sodium butyrate treatment. Shown are mean and SEM of three independent experiments. **$p<0.001$, ***$p<0.0001$, ****$p<0.00001$.

Figure S3

A

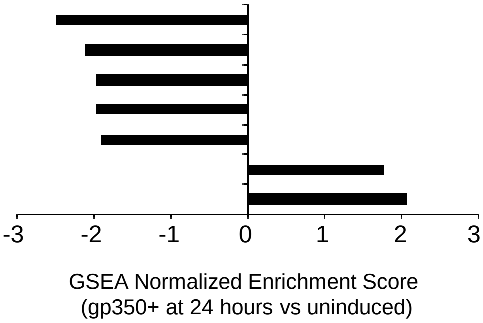

p-value	Direction of Gene Set Expression	
0.001	Naïve B-cell ↑	Plasma Cell ↓
0.002	Memory B-cell ↑	Blood Plasma Cell ↓
0.002	GC B cell ↑	Plasma Cell ↓
0.006	Naïve B-cell ↑	Plasma Cell ↓
0.01	Memory B cell ↑	BM Plasma Cell ↓
0.02	Plasmablast ↑	Naïve B-cell ↓
0.001	Blood Plasma Cell ↑	Naïve B-cell ↓

B

Upregulated in gp350+ cells vs uninduced and also in Plasma Cell vs Naïve B-cell

Naïve B cell ↑ Blood Plasma Cell ↓

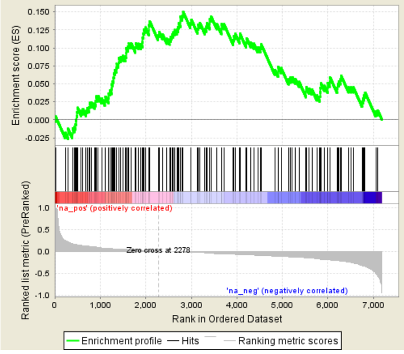

Naïve B cell ↑ Plasmablast ↓

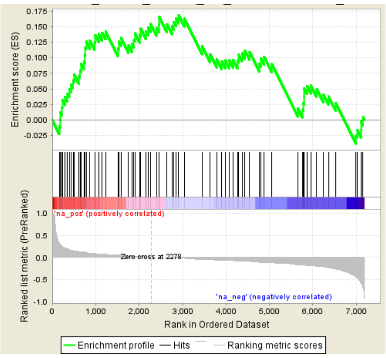

Downregulated in gp350+ cells vs uninduced and also in Plasma Cell vs Naïve B-cell

Memory B cell ↑ BM Plasma Cell ↓

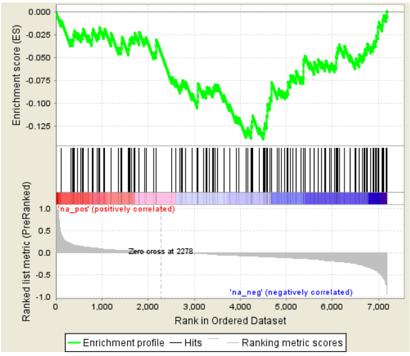

Naïve B cell ↑ Plasma Cell ↓

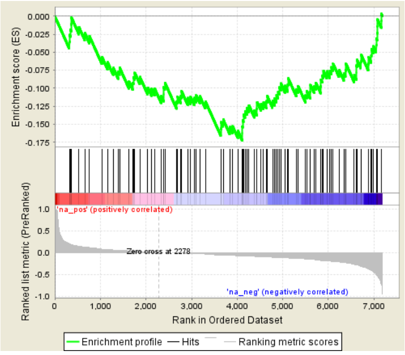

GC B cell ↑ Plasma Cell ↓

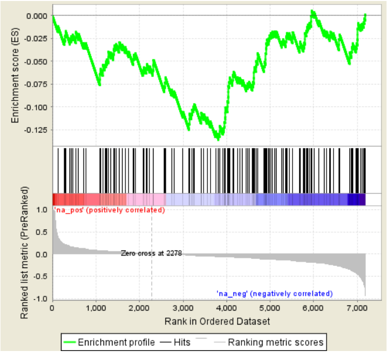

Memory B cell ↑ Plasma Cell ↓

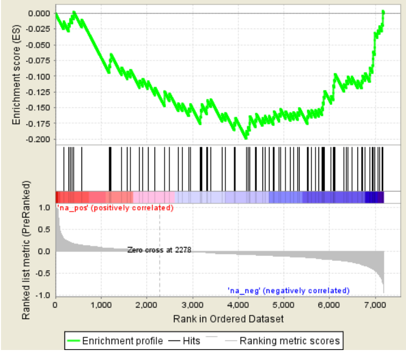

Naïve B cell ↑ BM Plasma Cell ↓

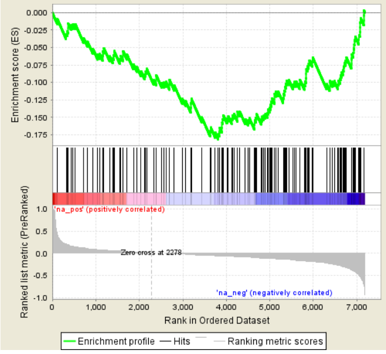

Figure S3. Related to **Figure 5**: Gene set enrichment analysis identified enrichment of plasmablast and plasma cell markers in gp350+ B-cells.

A. Changes in WCL composition between uninduced versus gp350+ P3HR1 cells (from the 24 hour timepoint) were analyzed by GSEA Preranked analysis. The differences in WCL3 proteome-wide abundances between uninduced and gp350+ WCL were cross-compared with published microarray B-cell differentiation datasets (Subramanian et al., 2005). Significantly enriched gene sets involving plasma cells were selected and visualized. Enrichment levels were expressed as Normal Enrichment Score (NES), calculated by the GSEA analysis. Significantly enriched gene were selected (p-values shown). GSEA demonstrated gp350+ cell enrichment for factors more highly expressed in plasmablasts and plasma cells, and depletion for factors more highly expressed in naïve or memory B-cells.

B. Data underlying the GSEA enrichment scores shown in (A).

Figure S4

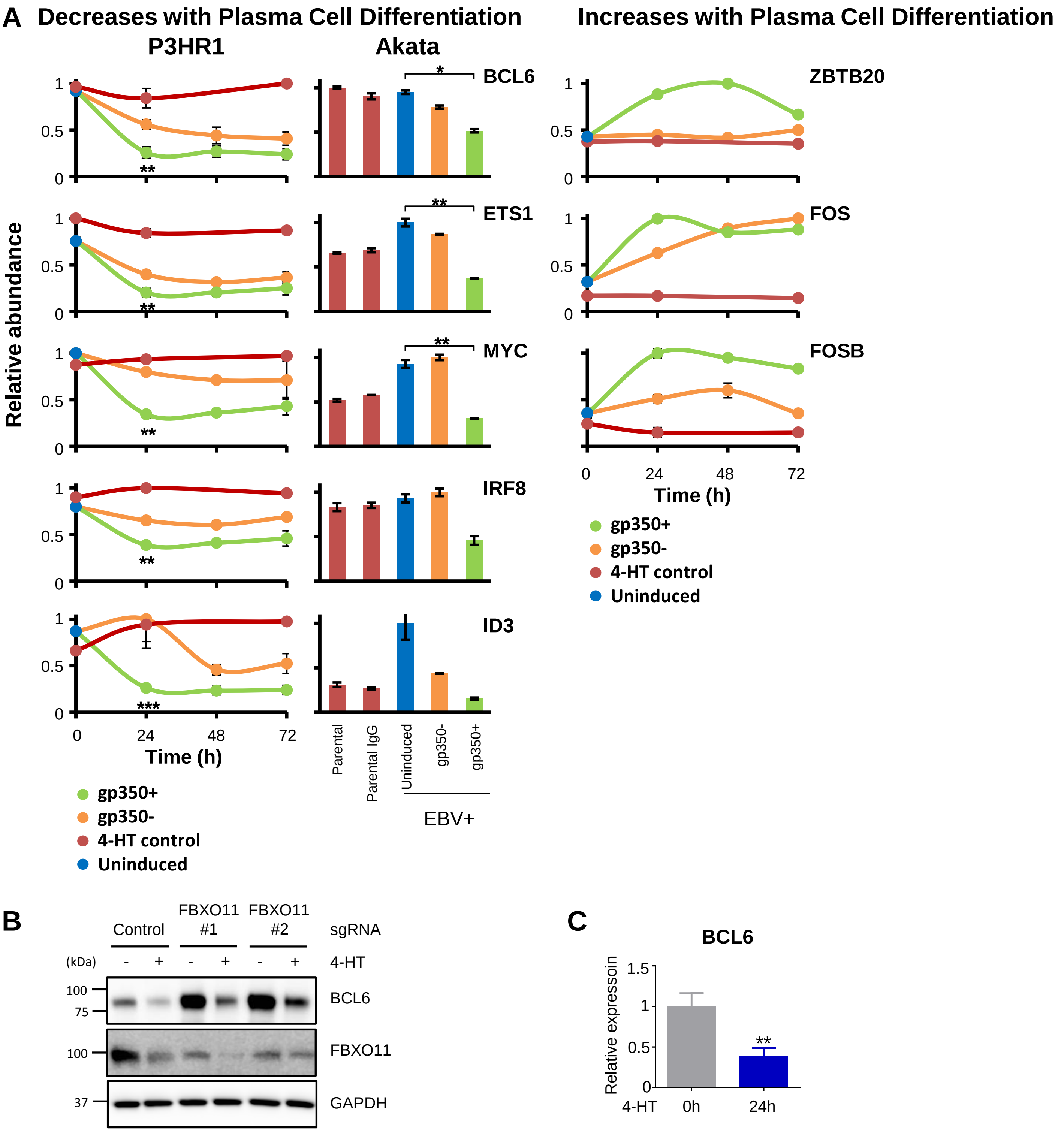
A
Decreases with Plasma Cell Differentiation
Increases with Plasma Cell Differentiation
P3HR1
Akata
Relative abundance
BCL6
ETS1
MYC
IRF8
ID3
ZBTB20
FOS
FOSB
*
**
***
1
0.5
0
0
24
48
72
Time (h)
Parental
Parental IgG
Uninduced
gp350-
gp350+
EBV+
gp350+
gp350-
4-HT control
Uninduced
B
Control
FBXO11 #1
FBXO11 #2
sgRNA
(kDa)
4-HT
- + - + - +
100
75
37
BCL6
FBXO11
GAPDH
C
BCL6
Relative expressoin
1.5
1
0.5
0
4-HT
0h
24h
**

Figure S4. Related to **Figure 5**: EBV lytic replication remodels multiple B-cell transcription factors that regulate plasma cell differentiation.

A. Temporal profiles of representative transcription factors whose abundance decreases or increases both with EBV lytic replication and plasma cell differentiation. p-values were calculated as described in Figure 1B: *** $p<0.005$, ** $p<0.05$, *$p<0.075$. ZBTB20 and FOS were only quantified in P3HR1 replicate WCL1. FOSB was only quantified in P3HR1 cells, and not Akata cells.

B. EBV downregulates BCL6 in an FBXO11-independent manner. P3HR1-ZHT/RHT Cas9+ cells expressing control versus two independent single guide RNAs (sgRNAs) against the ubiquitin ligase FBXO11 were treated with 4-HT for 72 hours then lysates immunoblotted, as shown.

C. Downregulation of BCL6 transcripts after induction in P3HR1-ZHT/RHT cells by qRT-PCR. Shown are mean and SEM of three independent experiments. *** $P < 0.01$.

Figure S5

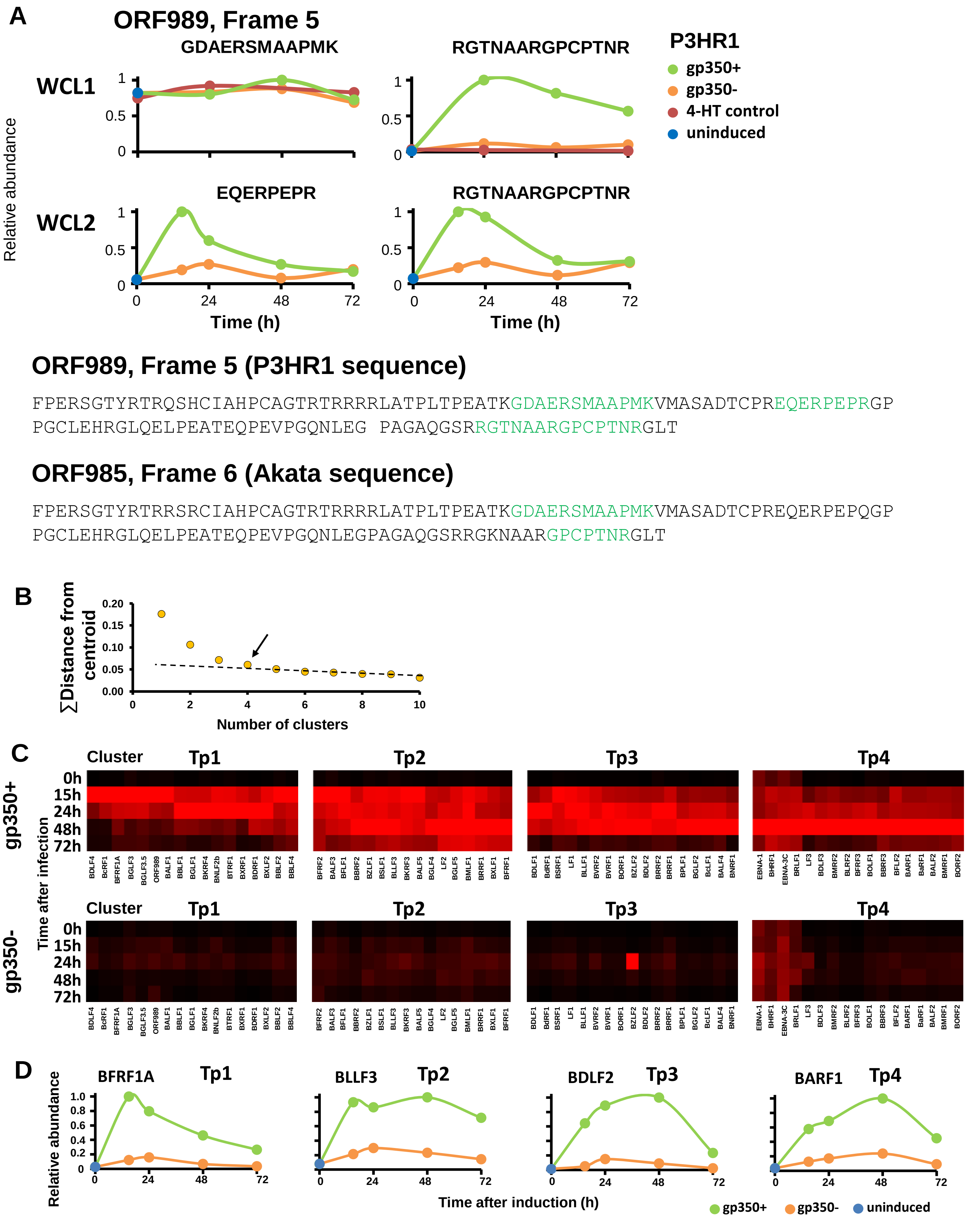
A
ORF989, Frame 5
GDAERSMAAPMK
RGTNAARGPCPTNR
P3HR1
gp350+
gp350-
4-HT control
uninduced
WCL1
WCL2
EQERPEPR
RGTNAARGPCPTNR
Relative abundance
0
0.5
1
0
24
48
72
Time (h)
ORF989, Frame 5 (P3HR1 sequence)
FPERSGTYRTRQSHCIAHPCAGTRTRRRRLATPLTPEATKGDAERSMAAPMKVMASADTCPREQERPEPRGP
PGCLEHRGLQELPEATEQPEVPGQNLEG PAGAQGSRRGTNAARGPCPTNRGLT
ORF985, Frame 6 (Akata sequence)
FPERSGTYRTRRSRCIAHPCAGTRTRRRRLATPLTPEATKGDAERSMAAPMKVMASADTCPREQERPEPQGP
PGCLEHRGLQELPEATEQPEVPGQNLEGPAGAQGSRRGKNAARGPCPTNRGLT
B
∑Distance from centroid
0.20
0.15
0.10
0.05
0.00
0
2
4
6
8
10
Number of clusters
C
Cluster
Tp1
Tp2
Tp3
Tp4
gp350+
gp350-
Time after infection
0h
15h
24h
48h
72h
BDLF4 BcRF1 BFRF1A BGLF3 BGLF3.5 ORF989 BALF1 BBLF1 BGLF1 BKRF4 BNLF2b BTRF1 BXRF1 BDRF1 BXLF2 BBLF2 BBLF4
BFRF2 BALF3 BFLF1 BBRF2 BZLF1 BSLF1 BLLF3 BKRF3 BALF5 BGLF4 LF2 BGLF5 BMLF1 BRRF1 BXLF1 BFRF1
BDLF1 BdRF1 BSRF1 LF1 BLLF1 BVRF2 BVRF1 BORF1 BZLF2 BDLF2 BRRF2 BRRF1 BPLF1 BGLF2 BcLF1 BALF4 BNRF1
EBNA-1 BHRF1 EBNA-3C BRLF1 LF3 BDLF3 BMRF2 BLRF2 BFRF3 BOLF1 BBRF3 BFLF2 BARF1 BaRF1 BALF2 BMRF1 BORF2
D
BFRF1A
Tp1
BLLF3
Tp2
BDLF2
Tp3
BARF1
Tp4
Relative abundance
1.0
0.8
0.6
0.4
0.2
0
0
24
48
72
Time after induction (h)
gp350+
gp350-
uninduced

Figure S5, related to **Figure 7**. Peptides identified from two novel EBV ORFs.

A. Peptides quantified from novel ORF989, P3HR1 experiments WCL1 (upper panels) and WCL2 (lower panels). Note GDAERSMAAPMK may have been misidentified, and did not exhibit the expected temporal profile of a viral protein. Peptide GPCPTNR was identified in experiment WCL3, although insufficient ions were present for quantitation. Protein sequences of ORF989 are shown, indicating the position of the identified peptides both in the P3HR1 and corresponding Akata ORF sequences, although no peptides were quantified in our Akata experiment.

B. Number of temporal classes of EBV gene expression. We used the k-means approach with 1 to 10 classes to cluster viral proteins measured in experiment WCL2 (since this experiment included 4 rather than 3 time points of EBV replication) and assessed the summed distance of each protein from its cluster centroid. Although this summed distance necessarily becomes smaller as more clusters are added, the rate of decline decreases with each added group, eventually settling at a fairly constant rate of decline that reflects overfitting; clusters added prior to this point reflect underlying structure in the temporal protein data, whereas clusters subsequently added through overfitting are not informative. The point of inflexion fell between four and six classes, suggesting that even within sorted cells marked by gp350 late antigen expression, there are at least four distinct temporal protein profiles of viral protein expression. Profiles of proteins in each k-means cluster are shown in **Figure S5C-D**.

C. Temporal profiles of proteins in each k-means class were subjected to hierarchical clustering by Euclidian distance.

D. Temporal profiles of typical proteins from each cluster.

SUPPLEMENTAL EXPERIMENTAL PROCEDURES

Resources Table

REAGENT or RESOURCE	SOURCE	IDENTIFIER
Antibodies		
Anti-EBV gp350 (72A1)	BioXCell	N/A
Anti-IgM goat F(ab')$_2$	Southern Biotech	Cat# 2022-01
Anti-IgM-FITC goat	Southern Biotech	Cat# 2020-02
Anti-IgM-biotin goat	Southern Biotech	Cat# 2020-08
Anti-IgG goat F(ab')$_2$	Southern Biotech	Cat# 2042-01
Anti-EBV early antigens	Novus Biologicals	Cat# NBP2-34516
Anti-AIM2 rabbit mAb (D5X7K)	Cell Signaling Technologies	Cat# 12948
Anti-STAT2	Bethyl	Cat# A303-512A-T
Anti-IRF1 rabbit mAb (D5E4)	Cell Signaling Technologies	Cat# 8478
Anti-IRF4 rabbit Ab	Cell Signaling Technologies	Cat# 4964
Anti-CD79A rabbit mAb (D1X5C)	Cell Signaling Technologies	Cat# 13333
Anti-ubiquitin mouse mAb (P4D1)	Cell Signaling Technologies	Cat# 3936
Anti-PDCD4 rabbit mAb (D29C6)	Cell Signaling Technologies	Cat# 9535
Anti-BCL6 rabbit mAb (D412V)	Cell Signaling Technologies	Cat# 14895
Anti-α-Tubulin	Sigma	Cat# T5168
Anti-GAPDH mouse mAb	Proteintech	Cat# 60004-1-1g
Anti-HA.11	Covance	Cat# MMS-101P
Anti-FBXO11	Bethyl	Cat# A301-177A-T
AF488 goat anti-mouse	Molecular Probes	Cat# A-11029
AF568 goat anti-mouse	Molecular Probes	Cat# A-11031
HRP-conjugated anti-mouse	Cell Signaling Technologies	Cat# 7076
HRP-conjugated anti-rabbit	Cell Signaling Technologies	Cat# 7074
Anti-goat bovine IgG	Jackson Immunolabs	Cat# 805-035-180
Polyclonal rabbit anti-human IgG	Dako	Cat # A 0423
Biological Samples		
Chemicals, Peptides, and Recombinant Proteins		
Tandem mass tag (TMT) 10-plex isobaric reagents	Thermo Fisher	Cat# 90110
HPLC water	VWR	Cat# 23595.328
LC-MS grade Acetonitrile	Merck	Cat# 1.00029.2500
Formic acid	Thermo Fisher	Cat# 85178
Aminooxy-biotin	Biotium	Cat# 90113
Sodium meta-periodate	Thermo Fisher	Cat# 20504
Streptavidin agarose beads	Pierce	Cat# 20359

NeutrAvidin agarose beads	Thermo Fisher	Cat# 29200
Cy-5 fluorophore for 72A1 labeling	Abcam	Cat# ab188288
Bortezomib	Apex Bio	Cat# A2614
Idelalisib	Apex Bio	Cat# A3005
Fostamatinib	Apex Bio	Cat# A8332
Pepstatin A	Calbiochem	Cat# 516481
E-64 Protease Inhibitor	Calbiochem	Cat# 324890
Cathepsin Inhibitor I	Calbiochem	Cat# 219415
MLN4924	Active Biochem	Cat# A-1139
16% formaldehyde	Ted Pella Inc.	Cat# 9658705
Hoechst	Molecular Probes	Cat# 33258
Unless otherwise noted, all other chemicals	Sigma	
Complete Protease Inhibitor Cocktail	Roche	Cat# 11836153001
Critical Commercial Assays		
Dual Glo Luciferase Assay Kit	Promega	Cat# E2920
Power SYBR Green RNA-to-CT 1-Step Kit	Applied Biosystems	Cat# 4389986
BCA Protein Assay Kit	ThermoFisher Scientific	Cat# 23225
Deposited Data		
Raw Mass Spectrometry Data Files	This paper; Proteome Xchange	
Experimental Models: Cell Lines		
P3HR1 cl.16	(Rabson et al., 1983)	N/A
P3HR1-ZHT/RHT	(Calderwood et al., 2008)	N/A
Experimental Models: Organisms/Strains		
Recombinant DNA		
pFR_CrPV_xb	Addgene	#11509
lentiGuide-puro	Addgene	#52963
pHAGE-LMP2A	This paper	N/A
MSCV-GFP	Addgene	#41034
Sequence-Based Reagents		
Primer for sgFBXO11 #1 cloning 5'-CACCGATATCTTCTCCACAGCACCT-3'	IDT	N/A
Primer for sgFBXO11 #1 cloning 5'-AAACAGGTGCTGTGGAGAAGATATC-3'	IDT	N/A
Primer for sgFBXO11 #2 cloning 5'-CACCGTTTCACATCGAACCACTGTA-3'	IDT	N/A
Primer or sgFBXO11 #2 cloning 5'-AAACTACAGTGGTTCGATGTGAAAC-3'	IDT	N/A
Primer GAPDH for 5'-TGCACCACCAACTGCTTAGC-3'	IDT	N/A
Primer GAPDH rev 5'-GGCATGGACTGTGGTCATGAG-3'	IDT	N/A
Primer C3 for 5'-CCTGGACTGCTGCAACTACA-3'	IDT	N/A

Primer C3 rev 5'-GCAATGATGTCCTCATCCAG-3'	IDT	N/A
Primer C4A for 5'-GGAGAAGCTGAATATGGGCA-3'	IDT	N/A
Primer C4A rev 5'-GATGTGAGCTCTGCCTCCTC-3'	IDT	N/A
Primer MASP1 for 5'-CACTGTCCCAGATGGGTTTC-3'	IDT	N/A
Primer MASP1 rev 5'-GCACCTGGTCCTCAGTTTCT-3'	IDT	N/A
Primer BCL6 for 5'-GTTTCCGGCACCTTCAGACT-3'	IDT	N/A
Primer BCL6 rev 5'-CTGGCTTTTGTGACGGAAAT-3'	IDT	N/A
Software and Algorithms		
In-house mass spectrometry data analysis software	(Huttlin et al., 2010)	N/A
SEQUEST	(Eng et al., 1994)	N/A
XLStat	Addinsoft	N/A
STRING database	http://string-db.org/	N/A
DAVID software	https://david.ncifcrf.gov/	N/A
Cluster 3.0	Stanford University	N/A
Java Treeview	SourceForge.net	N/A
Other		
Orbitrap Fusion Mass Spectrometer	ThermoFisher Scientific	Cat# IQLAAEGAAP FADBMBCX
Orbitrap Fusion Lumos Mass Spectrometer	ThermoFisher Scientific	Cat# IQLAAEGAAP FADBMBHQ
Easy-nLC 1000	ThermoFisher Scientific	Cat# LC120

Cell lines
P3HR1, P3HR1-ZHT/RHT and EBV-positive Akata cells were cultured in RPMI (Thermo Fisher) supplemented with fetal bovine serum (10% v/v) and penicillin/streptomycin (Gibco) at 37°C in 5% CO_2. For selection, G418 (Gemini Bio-Products, P3HR1-ZHT/RHT, EBV-positive Akata cells) and hygromycin (Calbiochem, P3HR1-ZHT/RHT) were used. 293T cells were grown in DMEM, supplemented with fetal bovine serum (10% v/v) and penicillin/streptomycin (Gibco) at 37°C in 5% CO_2. All cells were confirmed to be mycoplasma negative.

Epstein-Barr virus reactivation
Epstein-Barr virus lytic replication cycle was reactivated in P3HR1-ZHT/RHT cells or mock-induced in parental P3HR1 cells by addition of 400 nM 4-HT (Sigma) to P3HR1-ZHT/RHT at a density of 3 x 10^5 cells/ml or by addition of 20 ng/ml phorbol 12-myristate 13-acetate (TPA) (Sigma). For later time points of induction, TPA/sodium butyrate was removed after 24h. For inductions in the presence of inhibitors, the following concentrations were used: bortezomib (100 nM), idelalisib (200 nM), fostamatinib (100 nM), pepstatin A (1 µg/ml), E-64 protease inhibitor (1 µM), cathepsin inhibitor (12.5 µM), MLN4924 (10 µM), acyclovir (50 µg/ml). EBV lytic replication was induced in Akata-EBV+ cells or mock-induced in parental Akata EBV-negative cells by addition of anti-human IgG (1:2000at a density of 5 x 10^5 cells/ml, as previously described (Takada and Ono, 1989). Where indicated, Akata cells were induced by addition of 3 mM sodium butyrate (Sigma).

Flow cytometry
For cell sorting, P3HR1-ZHT/RHT were washed once in PBS and incubated with the anti-gp350 primary mouse monoclonal antibody 72A1 on ice, followed by three washes in PBS/0.5% BSA and incubation with the secondary goat anti-mouse 488 antibody. For ZHT/RHT cells, flow sorting was performed on live cells on a BD FACSAria sorter, using cell surface gp350 expression. The parental clone P3HR1 cl.16 was mock induced with 4-hydroxytamoxifen or left uninduced, stained with PI (Molecular Probes) and FACS sorted, based on PI negativity. For intracellular staining of IgM and gp350, cells were fixed with 4% formaldehyde (Ted Pella, Inc.) and

permeabilized with 90% ice-cold methanol for 30 min. After one wash step in PBS/0.5% BSA, cells were incubated with anti-IgM-FITC, followed by incubation with anti-EBV-gp350 (72A1) on ice, as described above. Cells were washed in PBS twice before being analyzed on a FACSCalibur instrument. Data was processed using FlowJo software. For intracellular flow cytometry analysis, cells were fixed in 4% formaldehyde (Ted Pella, Inc.) and permeabilized with 90% ice-cold methanol for 30 min. Cells were incubated with primary antibody in PBS/0.5% BSA and secondary antibody on ice and analysed using a FACSCalibur instrument.

Immunoblot Analysis
Cell pellets were lysed SDS/Tris buffer and denatured at 95°C for 5 min. Samples were sonicated for 15 seconds and equal amounts of proteins were separated by SDS-PAGE using 10% or 12% Bis-Tris polyacrylamide gels. Samples were transferred to nitrocellulose membranes. Membranes were blocked in TBS/5% non-fat dried milk/0.2% Tween and incubated with indicated primary antibodies at 4°C overnight, washed in TBST three times for 10 min, followed by a 45 min incubation with HRP-conjugated secondary anti-mouse, anti-rabbit (Cell Signaling Technologies) or anti-goat (Jackson Immunolab) antibody, respectively. Blots were washed in TBST for 10 min, three cycles. Bands were visualized, using enhanced chemoluminescent substrate (PerkinElmer). Band intensities were measured using a Carestream Molecular Imaging workstation.

Immunoprecipitation
For immunoprecipitation, 3 x 10^7 cells were washed twice with PBS and lysed in 1.5 ml NP40 lysis buffer, supplemented with Complete Protease Inhibitor Cocktail (Roche), 10mM glycerophosphate, 2mM sodium pyrophosphate, 1mM PMSF and 10 mM N-Ethylmaleimide (NEM) for 15 min. All steps were performed on ice or at 4°C. Nuclear debris was removed by centrifugation at 13,000 x g for 15 min. Lysates were incubated with 10 µg anti-IgM-biotin antibody (Southern Biotech) for 1h, followed by incubation with 20 µl NeutrAvidin beads (Thermo Fisher) for 1h. After four washes in NP40 lysis buffer, supplemented with Complete Protease Inhibitor Cocktail (Roche), 10mM glycerophosphate, 2mM sodium pyrophosphate, 1mM PMSF and 2mM NEM, samples were eluted in SDS sample buffer. SDS-PAGE and immunoblot were performed as described above.

Immunofluoresence Microscopy
P3HR1 cells were dried onto slides and fixed with 4% formaldehyde (Ted Pella, Inc.). Cells were then permeabilized with 0.5% triton X-100 for 10 min and blocked with PBS/1% BSA. Cells were stained with primary antibodies, anti-mouse AF568 sequentially for 1h in PBS/1% BSA and Hoechst for 10 min in PBS. Washes were performed with PBS/0.1% Tween-20. For analysis by confocal microscopy, cover glasses were mounted onto slides with ProLong Gold Antifade Mountant (Invitrogen) and images were taken using a Olympus Fluoview FV1000 confocal microscope.

Lentiviral transductions in 293T cells
293T cells were transfected in 6 well plates with 500ng of lentiviral plasmid, together with 500ng gag-pol and 50ng VSV-G for packaging. P3HR1 cells were transduced with 2ml lenti-/retrovirus, produced in 293T cells, two times and put under selection for 48h. For stable GFP expression, we used MSCV-N-Flag-HA-GFP, a gift from Wade Harper (Addgene plasmid # 41034) (Sowa et al., 2009).

Lentiviral vectors for sgRNA expression
For CRISPR/Cas9-mediated knockout of FBXO11 in P3HR1 cells, we used the FBXO11-specific sgRNAs, cloned into pLentiguide-puro, a gift from Feng Zhang (Addgene plasmid # 52963) (Sanjana et al., 2014) and sequence verified (Eton Bioscience).

Luciferase-based cap-dependent translation assay
5 x 10^6 P3HR1-ZHT/RHT cells were electroporated with 25 µg pFR_CrPv_xb (Petersen et al., 2006), a gift from Phil Sharp (Addgene plasmid # 11509) using a BioRad GenePulser (200V, 0.95 µF). After resting the cells for 24h, viral replication was induced by addition of 4-HT to the media. Subsequent luciferase assays were performed using a Dual Glo Luciferase Assay kit (Promega), according to the manifacturer's instructions. After 48h, 5 x 10^5 cells were lysed in 100 µl lysis buffer. Cap-dependent firefly (FF) luciferase and cap-independent renilla luciferase activity was quantified on a Molecular Devices plate reader. Firefly and Renilla luciferase were normalized to levels in uninduced cells and fold changes of normalized firefly luciferase activity, relative to normalized Renilla activity were calculated.

Quantitative reverse transcription PCR
RT-qPCR was performed on a BioRad CFX Connect Real-time PCR detection system, using Power SYBR Green RNA-to-CT 1-Step Kit (Applied Biosystems) for 40 cycles. Expression values and SEM relative to GAPDH expression were calculated using CFX Manager Software.

TMT-based proteomics
Plasma membrane profiling was performed as described previously, (Weekes et al., 2012; Weekes et al., 2014). Briefly, FACS sorted B-cells were washed twice with ice-cold PBS. Sialic acid residues were oxidized with sodium meta-periodate (Thermo Fisher) then biotinylated with aminooxy-biotin (Biotium). The reaction was quenched, cell numbers for each condition were normalized to 2×10^6 using a BioRad TC20 automated cell counter and the biotinylated cells were lysed in 1.6% Triton X-100 lysis buffer. Biotinylated glycoproteins were enriched with high affinity streptavidin agarose beads (Pierce) and washed extensively. Captured protein was denatured with DTT, alkylated with iodoacetamide (IAA, Sigma) and digested on-bead with trypsin (Promega) in 200 mM HEPES pH 8.5 for 3h. Tryptic peptides were collected.

For whole proteome samples, cells were washed twice with PBS, and 150 ul of 6M Guanidine/50 mM HEPES pH8.5 lysis buffer added. Samples were vortexed extensively then sonicated. Cell debris was removed by centrifuging at 13,000 g for 10 min twice. Dithiothreitol (DTT) was added to a final concentration of 5mM and samples were incubated for 20 min. Cysteines were alkylated with 15mM iodoacetamide and incubated 20 min at room temperature in the dark. Excess iodoacetamide was quenched with DTT for 15 min. Samples were diluted with 200 mM HEPES pH 8.5 to 1.5 M Guanidine followed by digestion at room temperature for 3 hr with LysC protease at a 1:100 protease-to protein ratio. Trypsin was then added at a 1:100 protease-to-protein ratio followed by overnight incubation at 37oC. The reaction was quenched with 1% formic acid, samples were spun at 21,000g for 10 min to remove debris and undigested protein, then subjected to C18 solid-phase extraction (Sep-Pak, Waters) and vacuum centrifuged to near-dryness.

In preparation for TMT labelling, desalted peptides were dissolved in 200 mM HEPES pH 8.5. For whole proteome samples, peptide concentration was measured by micro BCA (Pierce), and 50 mg of peptide labelled with TMT reagent. For plasma membrane samples, 100% of each peptide sample was labelled. TMT reagents (0.8 mg) were dissolved in 43 μL anhydrous acetonitrile and 5 μl added to peptide sample at a final acetonitrile concentration of 30% (v/v). Samples were labelled as follows: Experiment WCL1 and PM1: P3HR1-ZHT/RHT uninduced (TMT 126); P3HR1-ZHT/RHT induced 24h gp350- (TMT 127N); P3HR1-ZHT/RHT induced 48h gp350- (TMT 127C); P3HR1-ZHT/RHT induced 72h gp350- (TMT 128N); P3HR1-ZHT/RHT induced 24h gp350+ (TMT 128C); P3HR1-ZHT/RHT induced 48h gp350+ (TMT 129N); P3HR1-ZHT/RHT induced 72h gp350+ (TMT 129C); P3HR1 (TMT 130N); P3HR1+4-HT 24h (TMT 130C); P3HR1+4-HT 72h (TMT 131). For Experiment WCL2: P3HR1-ZHT/RHT uninduced (TMT 126); P3HR1-ZHT/RHT induced 15h gp350+ (TMT 127N); P3HR1-ZHT/RHT induced 15h gp350- (TMT 127C); P3HR1-ZHT/RHT induced 24h gp350+ (TMT 128N); P3HR1-ZHT/RHT induced 15h gp350- (TMT 128C); P3HR1-ZHT/RHT induced 48h gp350+ (TMT 129N); P3HR1-ZHT/RHT induced 48h gp350- (TMT 129C); P3HR1-ZHT/RHT induced 72h gp350+ (TMT 130N); P3HR1-ZHT/RHT induced 72h gp350- (TMT 130C); P3HR1-ZHT/RHT uninduced, biological replicate (TMT 131). Experiment WCL3: P3HR1-ZHT/RHT uninduced (TMT 126); P3HR1+4-HT 24h (TMT 127N); P3HR1-ZHT/RHT induced 24h gp350- (TMT 127C); P3HR1-ZHT/RHT induced 24h gp350+ (TMT 128N); P3HR1+4-HT 48h (TMT 128C); P3HR1-ZHT/RHT induced 48h gp350- (TMT 129N); P3HR1-ZHT/RHT induced 48h gp350+ (TMT 129C); P3HR1+4-HT 72h (TMT 130N); P3HR1-ZHT/RHT induced 72h gp350- (TMT 130C); P3HR1-ZHT/RHT induced 72h gp350+ (TMT 131). Akata WCL experiment: EBV negative untreated duplicates (TMT 126 and 127N); EBV positive uninduced (TMT 127C and 128N); EBV positive mock induced (TMT 128C and 129N); EBV positive gp350 negative (TMT 129C and 130N); EBV positive gp350 positive (TMT 130C and 131).
Following incubation at room temperature for 1 hr, the reaction was quenched with hydroxylamine to a final concentration of 0.5% (v/v). TMT-labeled samples were combined at a 1:1:1:1:1:1:1:1:1:1 ratio. The sample was vacuum-centrifuged to near dryness and subjected to C18 solid-phase extraction (SPE) (Sep-Pak, Waters).
Offline high pH reversed-phase fractionation of peptides from all WCL experiments and offline tip-based strong cation exchange fractionation of the PM sample were performed, and WCL peptide fractions combined as described previously (Weekes et al., 2014).

Liquid Chromatography and Tandem Mass Spectrometry

Mass spectrometry data were acquired using an Orbitrap Lumos (experiments WCL1, PM1, Akata WCL) coupled with a Proxeon EASY-nLC 1000 LC pump (Thermo Fisher Scientific, San Jose, CA), or an Orbitrap Fusion (experiments WCL2-3). We quantified TMT reporter ions from the MS3 scan (McAlister et al., 2012; Ting et al., 2011).

For Orbitrap Lumos experiments, peptides were separated on a 75 mm inner diameter microcapillary column packed with 0.5 cm of Magic C4 resin (5 mm, 100 A°,Michrom Bioresources) followed by approximately 20 cm of GP118 resin (1.8 mm, 120 A°, Sepax Technologies). Peptides were separated using a 3 hr gradient of 6 to 30% acetonitrile in 0.125% formic acid at a flow rate of 300 nl/min. Each analysis used an MS3-based TMT method (McAlister et al., 2012; Ting et al., 2011). The scan sequence began with an MS1 spectrum (Orbitrap analysis, resolution 120,000, 350-1400 Th, AGC target 5 x 10^5, maximum injection time 100 ms). 'Rapid' was selected for MS2 analysis, which consisted of CID (quadrupole ion trap analysis, AGC 1.8 x 10^4, NCE 35, maximum injection time 120 ms). For MS3 analysis, precursors were fragmented by HCD prior to Orbitrap analysis (NCE 55, max AGC 3 x 10^5, maximum injection time 120 ms, isolation specificity 0.5 Th, resolution 60,000).

For Orbitrap Fusion experiments, peptides were separated as above. The scan sequence began with an MS1 spectrum (Orbitrap analysis, resolution 120,000, 400-1400 Th, AGC target 2 x 10^5, maximum injection time 100 ms). 'Rapid' was selected for MS2 analysis, which consisted of CID (quadrupole ion trap analysis, AGC 4 x 10^3, NCE 35, maximum injection time 150 ms). The top ten precursors were selected for MS3 analysis, in which precursors were fragmented by HCD prior to Orbitrap analysis (NCE 55, max AGC 5 x 10^4, maximum injection time 150 ms, isolation specificity 0.5 Th, resolution 60,000) (McAlister et al., 2012).

Data Analysis

Mass spectra were processed using a Sequest-based in-house software pipeline as described previously (Weekes et al., 2014). Briefly, MS spectra were converted to mzXML using a modified version of ReAdW.exe. A combined database was constructed from (a) a combined human Uniprot and Trembl database (February 4th, 2014), (b) P3HR1 strain EBV, (c) Akata strain EBV (d) all open reading frames from a six-frame translation of P3HR1 strain EBV, (e) all open reading frames from a six-frame translation of Akata strain EBV and (f) common contaminants such as porcine trypsin and endoproteinase LysC. The combined database was concatenated with a reverse database composed of all protein sequences in reversed order. Searches were performed using a 20 ppm precursor ion tolerance. Product ion tolerance was set to 0.03 Th. TMT tags on lysine residues and peptide N termini (229.162932 Da) and carbamidomethylation of cysteine residues (57.02146 Da) were set as static modifications, while oxidation of methionine residues (15.99492 Da) was set as a variable modification. To control the fraction of erroneous protein identifications, we used a target-decoy strategy (Elias and Gygi, 2007, 2010). Peptide spectral matches (PSMs) were filtered to an initial peptide-level false discovery rate (FDR) of 1% with subsequent filtering to attain a final protein-level FDR of 1%. PSM filtering was performed using a linear discriminant analysis, as described previously (Huttlin et al., 2010), considering the following parameters: XCorr, DCn, missed cleavages, peptide length, charge state, and precursor mass accuracy. Protein assembly was guided by principles of parsimony to produce the smallest set of proteins necessary to account for all observed peptides. Where all PSMs from a given EBV protein could be explained either by a canonical gene or an ORF from the six-frame translation, the canonical gene was picked in preference. For Figure 6A-F, to identify host proteins co-regulated by EBV and HCMV, raw files comprising EBV P3HR1 experiments WCL1-3 and PM1 were searched in combination with HCMV files from experiments WCL1-2 and PM1-2 (Weekes et al., 2014). For Figure 6G, raw files comprising EBV experiment PM1 were re-searched in combination with KHSV K5 files from the experiment described in this paper (Timms et al., 2013), against the human Uniprot database (February 4th, 2014).

Proteins were quantified by summing TMT reporter ion counts across all matching peptide-spectral matches using in-house software, as described previously (Pease et al., 2013). Briefly, a 0.003 Th window around the theoretical m/z of each reporter ion (126, 127N, 127C, 128N, 128C, 129N, 129C, 130N, 130C, 131) was scanned for ions, and the maximum intensity nearest to the theoretical m/z was used. We required every individual peptide used for quantitation to contribute sufficient TMT reporter ions (minimum of 1,250 per spectrum) so that each on its own provided a representative picture of relative protein abundance (McAlister et al., 2012). We additionally employed an isolation specificity filter to minimize peptide coisolation (Ting et al., 2011). Peptide-spectral matches with poor quality MS3 spectra (more than 9 TMT channels missing and/or a combined signal:noise ratio of less than 250 across all TMT reporter ions) or no MS3 spectra at all were excluded from quantitation. Protein quantitation values were exported for further analysis in Excel. Reverse and contaminant proteins were removed, then two

normalisation steps were employed. (A) each reporter ion channel was summed across all quantified proteins and normalized assuming equal protein loading across all 10 samples. (B) for calculation of p-values for P3HR1 experiments WCL1-3, the resulting values needed first to be normalised *between* experiments to enable their direct comparison. For each of experiments WCL1-3, each reporter ion value for each protein was therefore normalised to the sum of the uninduced and 24h gp350+ samples for that protein. For the WCL2 series, the average of the two unstimulated controls was used in the calculation. Normalised values were imported into Perseus version 1.5.1.6 (Tyanova et al., 2016), and 2-tailed t-test values calculated. For calculation of p-values for the Akata WCL experiment, as biological duplicates were quantified in the same experiment, only normalisation (A) described above was needed. One-way ANOVA values were added using Perseus. For t-test and ANOVA p-values, correction for multiple hypothesis testing was further applied using the Benjamini-Hochberg method. As Perseus only indicates p-values that are significant after correction but does not display or export corrected values, the method was employed via a macro written in Excel by one of the authors. For Figure S1C, the matrix of Pearson correlations was generated using Perseus. For Figures 1D and 7B, r^2 values were calculated in Excel. Gene Ontology terms were downloaded from www.uniprot.org. Hierarchical centroid clustering based on uncentered Pearson correlation for Figure S1D, or Euclidian distance for Figure S5C was performed using Cluster 3.0 (Stanford University) and visualized using Java Treeview (http://jtreeview.sourceforge.net). Statistical analyses including k-means clustering were performed using XLStat (Addinsoft). The cellular concentrations of viral proteins in whole cell lysates (Figure 7A) were calculated using a 'proteomic ruler' approach implemented in Perseus plugin (http://www.coxdocs.org/doku.php?id=perseus:user:plugins:proteomicruler:estimatecopynumbers). Briefly, intensity values that had been normalised assuming equal protein loading across all 10 samples were imported into Perseus. For P3HR1 samples, intensities were further corrected to normalise between experiments WCL1-3. Protein sequence lengths and molecular weights were downloaded for human, P3HR1 and Akata from Uniprot and additionally imported. Averaging was performed using the 'same normalisation for all columns' option. Scaling was using the histone proteomic ruler, assuming a ploidy of 2.

Pathway Analysis was performed using the Database for Annotation, Visualization and Integrated Discovery (DAVID) (Huang da et al., 2009) version 6.8 with default settings. A given cluster was always searched against a background of all proteins quantified within the relevant experiment. To generate lists of proteins for DAVID enrichment analysis, for P3HR1 WCL data, proteins were included if they were quantified in all of experiments WCL1-3. All proteins quantified in the Akata WCL and P3HR1 PM experiment were included. Lists of down- or up-regulated proteins were generated by employing a minimum 2-fold change, and p-value as described in Figure 1. Fold enrichment and Benjamini-Hochberg corrected p-values were downloaded from the DAVID website.
For Figure 6B we used the STRING database to identify known protein interactions (http://string-db.org/). For prediction of EBV viral proteins present at the PM (Figure 7C), we used a strategy described in our recent publication (Weekes et al., 2014). We initially used human protein data to delineate a cutoff that predicted PM location. For or every GO-annotated human protein quantified in experiment PM1, we calculated the ratio of peptides (experiments PM1)/(average (experiments WCL1, WCL2, WCL3)). 87% of proteins that were GO-defined non-PM had a ratio of <0.35; 84% of human proteins scoring above 0.35 were annotated as PM proteins, demonstrating the predictive value of this metric (Figure 7C). Applying this filter, we defined 9 high-confidence viral PM proteins, which included the majority of viral proteins previously identified at the surface of cells induced to replicate EBV, and excluded all proteins unlikely to be present at the cell surface based on their known function.

Database generation

A P3HR1 Uniprot database was generated from published sequence data (www.uniprot.org, accession number LN827548) (Palser et al., 2015). An Akata Uniprot database was downloaded (http://www.uniprot.org/citations/23152513). A678 and B95.8 strain EBV proteins have high sequence homology to P3HR1, and were included in addition to the P3HR1 protein in regions in which the P3HR1 genome was incompletely sequenced (A678 in preference to B95.1 where both were available). Specific additional proteins included for A678 were (Uniprot or Trembl where indicated): EBNAL-LP (Q1HVI8), EBNA3 (Q69138), EBNA6 (Q69140), BLLF1 (P68343), EBNA1 (Q1HVF7), BPLF1 (Q1HVH9). Specific additional proteins included for B95.8 were: LF3 (P0C727), BHLF1 (P03181), EC-RF4 (P03235), BCRF2 (Trembl Q8AZK8), BSLF2 (Trembl Q9QCF1). A 6-frame translation of the full P3HR1 and Akata nucleotide sequences were additionally generated, requiring each ORF to contain a minimum of >= 7 amino acids. For each of the 6 reading frames, each potential polypeptide was sequentially numbered starting from 1.

In a small number of cases, data searches identified viral proteins with the same gene name, but belonging to more than one EBV strain. In each case, the relevant Uniprot or Trembl terms, strains and protein names have been indicated, separated by '/' in Table S1. Signal : noise values and peptide numbers were summed to generate a single entry for each EBV protein.

Data and software availability
Mass spectrometry data reported in this paper will be made available through the PRIDE Archive, a member of the ProteomeXchange (PX) consortium.

Gene Set Enrichment Analysis (GSEA) Analysis.
The difference in the proteomics data at 24 hour post induction compared to uninduced sample was used to generate a ranked list for GSEA Preranked anlaysis using the Molecular Signatures Database v5.2 (C7:immunologic signatures) (Subramanian et al., 2005). Gene sets with nominal p value < 0.05 and false discovery rate (FDR) <0.25 were defined as significantly enriched gene sets, which were selected for visualization. GSEA first calculates the enrichment score (ES), which reflects the degree to which a gene set is overrepresented at the top or bottom of a ranked list of genes. GSEA calculates the ES by walking down the ranked list of genes, increasing a running-sum statistic when a gene is in the gene set and decreasing it when it is not. The magnitude of the increment depends on the correlation of the gene with the phenotype. The ES is the maximum deviation from zero encountered in walking the list. A positive ES indicates gene set enrichment at the top of the ranked list; a negative ES indicates gene set enrichment at the bottom of the ranked list. The normalized enrichment score (NES) is the primary statistic for examining gene set enrichment results. By normalizing the ES, GSEA accounts for differences in gene set size and in correlations between gene sets and the expression dataset; therefore, the normalized enrichment scores (NES) can be used to compare analysis results across gene sets. GSEA determines NES as follows: NES is equal to the actual ES, divided by the mean of ESs against all permutations of the dataset.

Supplemental References:

Calderwood, M.A., Holthaus, A.M., and Johannsen, E. (2008). The Epstein-Barr virus LF2 protein inhibits viral replication. J Virol *82*, 8509-8519.

Elias, J.E., and Gygi, S.P. (2007). Target-decoy search strategy for increased confidence in large-scale protein identifications by mass spectrometry. Nat Methods *4*, 207-214.

Elias, J.E., and Gygi, S.P. (2010). Target-decoy search strategy for mass spectrometry-based proteomics. Methods Mol Biol *604*, 55-71.

Eng, J.K., McCormack, A.L., and Yates, J.R. (1994). An approach to correlate tandem mass spectral data of peptides with amino acid sequences in a protein database. J Am Soc Mass Spectrom *5*, 976-989.

Huang da, W., Sherman, B.T., and Lempicki, R.A. (2009). Systematic and integrative analysis of large gene lists using DAVID bioinformatics resources. Nat Protoc *4*, 44-57.

Huttlin, E.L., Jedrychowski, M.P., Elias, J.E., Goswami, T., Rad, R., Beausoleil, S.A., Villen, J., Haas, W., Sowa, M.E., and Gygi, S.P. (2010). A tissue-specific atlas of mouse protein phosphorylation and expression. Cell *143*, 1174-1189.

McAlister, G.C., Huttlin, E.L., Haas, W., Ting, L., Jedrychowski, M.P., Rogers, J.C., Kuhn, K., Pike, I., Grothe, R.A., Blethrow, J.D., *et al.* (2012). Increasing the multiplexing capacity of TMTs using reporter ion isotopologues with isobaric masses. Anal Chem *84*, 7469-7478.

Palser, A.L., Grayson, N.E., White, R.E., Corton, C., Correia, S., Ba Abdullah, M.M., Watson, S.J., Cotten, M., Arrand, J.R., Murray, P.G., *et al.* (2015). Genome diversity of Epstein-Barr virus from multiple tumor types and normal infection. J Virol *89*, 5222-5237.

Pease, B.N., Huttlin, E.L., Jedrychowski, M.P., Talevich, E., Harmon, J., Dillman, T., Kannan, N., Doerig, C., Chakrabarti, R., Gygi, S.P., *et al.* (2013). Global analysis of protein expression and phosphorylation of three stages of Plasmodium falciparum intraerythrocytic development. J Proteome Res *12*, 4028-4045.

Petersen, C.P., Bordeleau, M.E., Pelletier, J., and Sharp, P.A. (2006). Short RNAs repress translation after initiation in mammalian cells. Mol Cell *21*, 533-542.

Rabson, M., Heston, L., and Miller, G. (1983). Identification of a rare Epstein-Barr virus variant that enhances early antigen expression in Raji cells. Proc Natl Acad Sci U S A *80*, 2762-2766.

Sanjana, N.E., Shalem, O., and Zhang, F. (2014). Improved vectors and genome-wide libraries for CRISPR screening. Nat Methods *11*, 783-784.

Sowa, M.E., Bennett, E.J., Gygi, S.P., and Harper, J.W. (2009). Defining the human deubiquitinating enzyme interaction landscape. Cell *138,* 389-403.
Subramanian, A., Tamayo, P., Mootha, V.K., Mukherjee, S., Ebert, B.L., Gillette, M.A., Paulovich, A., Pomeroy, S.L., Golub, T.R., Lander, E.S., *et al.* (2005). Gene set enrichment analysis: a knowledge-based approach for interpreting genome-wide expression profiles. Proc Natl Acad Sci U S A *102*, 15545-15550.
Takada, K., and Ono, Y. (1989). Synchronous and sequential activation of latently infected Epstein-Barr virus genomes. J Virol *63*, 445-449.
Timms, R.T., Duncan, L.M., Tchasovnikarova, I.A., Antrobus, R., Smith, D.L., Dougan, G., Weekes, M.P., and Lehner, P.J. (2013). Haploid genetic screens identify an essential role for PLP2 in the downregulation of novel plasma membrane targets by viral E3 ubiquitin ligases. PLoS Pathog *9*, e1003772.
Ting, Y.S., Shaffer, S.A., Jones, J.W., Ng, W.V., Ernst, R.K., and Goodlett, D.R. (2011). Automated lipid A structure assignment from hierarchical tandem mass spectrometry data. J Am Soc Mass Spectrom *22*, 856-866.
Tyanova, S., Temu, T., Sinitcyn, P., Carlson, A., Hein, M.Y., Geiger, T., Mann, M., and Cox, J. (2016). The Perseus computational platform for comprehensive analysis of (prote)omics data. Nat Methods *13*, 731-740.
Weekes, M.P., Antrobus, R., Talbot, S., Hor, S., Simecek, N., Smith, D.L., Bloor, S., Randow, F., and Lehner, P.J. (2012). Proteomic plasma membrane profiling reveals an essential role for gp96 in the cell surface expression of LDLR family members, including the LDL receptor and LRP6. J Proteome Res *11*, 1475-1484.
Weekes, M.P., Tomasec, P., Huttlin, E.L., Fielding, C.A., Nusinow, D., Stanton, R.J., Wang, E.C., Aicheler, R., Murrell, I., Wilkinson, G.W., *et al.* (2014). Quantitative temporal viromics: an approach to investigate host-pathogen interaction. Cell *157*, 1460-1472.
